# Characterization of self-incompatibility genes in *Brassica rapa* var. toria and yellow sarson

**DOI:** 10.3389/fpls.2026.1857745

**Published:** 2026-07-02

**Authors:** Hemal Bhalla, Kumari Ankita, Aman Ahlawat, Surabhi S. Rode, Kunwar Harendra Singh, Subramanian Sankaranarayanan

**Affiliations:** 1 Department of Biological Sciences and Engineering, Indian Institute of Technology Gandhinagar, Palaj, Gujarat, India; 2 ICAR-Directorate of Rapeseed-Mustard Research, Bharatpur, Rajasthan, India; 3 ICAR-National Soybean Research Institute, Indore, Madhya Pradesh, India

**Keywords:** *Brassica*, crop improvement, self-incompatibility, toria, yellow sarson

## Abstract

Self-incompatibility (SI), a reproductive mechanism that prevents self-pollen from fertilizing the ovule, is widespread in flowering plants, including the Brassicaceae family, where it promotes outcrossing, genetic diversity, and hybrid vigor. Although prevalent in *Brassica rapa*, an economically vital crop, it remains poorly characterized in widely grown varieties, such as toria and yellow sarson, with prior studies primarily focused on *Brassica napus*. Given its potential for hybrid breeding and crop improvement in *Brassica rapa*, we characterized key SI-regulatory genes, analyzing their phylogenetic relationships, structure-function dynamics, and expression patterns. Our results indicate sequence, structural, and functional homology as well as conservation with previously known candidates. This study identified the integral role of the key players in regulating self-incompatibility and ROS dynamics. We identified SRK, FER, and ARC1 as essential for SI, while SRK, FER, and MLPK activated the ROS response. Our findings establish a foundation for harnessing this natural system to integrate agriculturally important traits and sustain them across generations via outcrossing.

## 1 Introduction

Self-incompatibility (SI) is a genetic mechanism in various families of flowering plants that promotes outcrossing, increases genetic diversity and hybrid vigor, and helps avoid inbreeding depression. In the Brassicaceae family, which includes economically important oilseed, vegetable, and leafy green crops, SI is of the sporophytic type. This system is governed by the polymorphic S-locus in a haplotype-dependent manner, such that matching haplotypes between stigma and pollen trigger an incompatible response (Nasrallah, 1997; Abhinandan et al., 2022; Bhalla et al., 2025a).

At the molecular level, the multi-allelic pollen ligand SP11/SCR (S-locus protein 11/S-cysteine-rich) interacts with the stigmatic receptor kinase SRK (S-locus receptor kinase) to orchestrate an incompatible response when haplotypes are identical (Kusaba et al., 2000; Takayama et al., 2001; Abhinandan et al., 2022; Huang et al., 2023; Bhalla et al., 2025a). This interaction activates downstream components including MLPK (M-locus protein kinase) (Kakita et al., 2007), which, together with SRK, phosphorylates and activates ARC1 (Armadillo repeat-containing 1), leading to the ubiquitin-mediated proteasomal degradation of compatibility factors such as Exo70A1 (Exocyst 70A1), GLO1 (Glyoxalase 1), and PLDα1 (Phospholipase D alpha 1) (Samuel et al., 2009; Sankaranarayanan et al., 2015; Scandola and Samuel, 2019; Abhinandan et al., 2022; Bhalla et al., 2025a).

Concurrently, activated SRK also activates the receptor-like kinase FERONIA (FER), which triggers a respiratory burst oxidase homolog (RBOHD/F)- mediated reactive oxygen species (ROS) burst that further restricts pollen acceptance (Zhang et al., 2021; Huang et al., 2023). In contrast, during a compatible response, interaction between completely dissimilar haplotypes, these signaling cascades are attenuated, thereby preventing degradation of compatibility factors, limiting ROS accumulation, and permitting pollen acceptance and germination.

Brassicaceae encompasses a wide range of food, oilseed, research, and condiment crops of major economic importance. *Brassica*, a genus, includes six major crop species: three diploid and three allotetraploid (Thakur et al., 2017; Sharma et al., 2025; Zhang et al., 2025). *Brassica rapa* L., a widely cultivated rapeseed is known for its oil content, favorable fatty acid profile, quality protein composition, preservative properties, and its use as a vegetable, flavoring agent, and condiment (Abul-Fadl et al., 2011; Cartea et al., 2019; Grygier, 2023). Despite this economic significance, the species often lacks high yield, robust disease resistance, and other agronomically important traits that could be introgressed through hybrid breeding and the exploitation of genetic diversity (Wang et al., 2022).

Previous work on self-incompatibility (SI) systems and their application in hybrid seed production and crop improvement has largely focused on *Brassica napus* (canola) (Abhinandan et al., 2022; Bhalla et al., 2025a). In contrast, research on SI in *Brassica rapa* L. has remained relatively limited, despite the species’ morphological and genetic diversity and its substantial economic importance. This has provided strong impetus to investigate its incompatibility systems and to harness the naturally occurring SI pathways in this species for breeding and crop improvement strategies. Our study has characterized the SI system in *Brassica rapa* L., providing insights into its similarities to previously known molecular players, their protein structures, and their functions. Our results suggest that the two-variety system, comprising self-incompatible toria and self-compatible yellow sarson lines of *B. rapa*, holds considerable potential for further functional studies and for deployment in breeding applications.

## 2 Materials and methods

### 2.1 Plant material and growth conditions

*Brassica rapa* varieties toria (TOR, self-incompatible) and yellow sarson (YS, self-compatible) seeds were obtained from the Indian Council of Agricultural Research - Directorate of Rapeseed-Mustard Research (ICAR-DRMR), Bharatpur, Rajasthan, India. Seeds were surface-sterilized using 0.05% SDS in 70% ethanol and plated on half-strength Murashige and Skoog (MS) medium, stratified at 4 °C for 3-7 days, then grown for 10 days at 22 °C with a 16 h:8 h light: dark cycle and 70% relative humidity. Plantlets were subsequently transferred to pots under the same conditions for acclimatization before experiments.

### 2.2 *In-silico* analysis and structure prediction

Sequences of the genes: *SRK* (**Supplementary Table 3**), *FER1* (**Supplementary Table 4**), *MLPK* (**Supplementary Table 5**), and *ARC1* (**Supplementary Table 6**) were retrieved from the NCBI database (Sayers et al., 2022) by performing nucleotide BLAST searches against species of the *Brassica* U’s triangle and *Arabidopsis*, using a 75% identity cutoff relative to the respective query sequences. Multiple sequence alignments of these gene sequences were performed using CLUSTAL W (Thompson et al., 1994). The representation and visualization of the multiple sequence alignment was conducted using N.ESPript (Gouet et al., 1999). Phylogenetic trees were constructed with MEGA11 (Tamura et al., 2021) using the maximum likelihood method with 1000 bootstrap replications, the Tamura-Nei nucleotide substitution model, and default parameters. The resulting trees were visualized using iTOL (Letunic and Bork, 2024).

Secondary structures of the protein sequences SRK, FER1, MLPK, and ARC1 were predicted using the PSIPRED server (McGuffin et al., 2000). The three-dimensional (3D) structural models were predicted using AlphaFold3, an AI model by Google DeepMind and Isomorphic Labs (Abramson et al., 2024). For each query sequence, the template-based modeling mode was selected, with all other parameters set to their defaults. Evolutionarily conserved functional domains in these proteins were identified using the NCBI Conserved Domain Database (Marchler-Bauer et al., 2009). Ramachandran plots were made using the PROCHECK online web server (Laskowski et al., 1993).

### 2.3 Cross-compatibility assay

Flowers of *Brassica rapa* var. toria and yellow sarson were emasculated on the day before crosses were conducted. *In-planta* manual pollination was conducted in both directions. The plants were grown under optimal conditions, and seeds were harvested to determine the number per pod.

Stigmas were pollinated in both directions for 12 h. These stigmas were subjected to an aniline blue assay to determine pollen attachment and pollen tube count.

### 2.4 Cloning and sequencing of genes

Primers were designed from previously known sequences in NCBI GenBank (**Supplementary Table 1**) (Sayers et al., 2022) using Primer3Plus (Untergasser et al., 2012). Total RNA was extracted using the RNeasy plant mini kit (Qiagen, Catalog: 74904), and first-strand cDNA was synthesized using the Thermo Scientific First Strand cDNA synthesis Kit (Catalog: K1612). The resulting cDNA was used as a template in PCR to optimize amplification of the product (**Supplementary Table 2**). The amplified products were cloned into the pCR4 -TOPO TA vector using the TOPO TA Cloning Kit for Sequencing (Catalog: 450030). The recombinant constructs were introduced into *E. coli* DB3.1 cells and selected on LB plates containing either ampicillin or kanamycin. Successful cloning and transformation were confirmed by colony PCR and restriction digestion. At least two independent plasmid clones were subjected to Sanger sequencing to verify the insert sequence.

### 2.5 Designing of oligonucleotides (S-ODNs and AS-ODNs)

Sense and antisense oligodeoxyribonucleotide (S-ODNs and AS-ODNs) pairs were designed using Sfold (**Supplementary Table 8**) (Ding, 2001, 2003; Ding et al., 2004). Potential off-target effects were assessed using NCBI BLAST (Camacho et al., 2023). ODNs were synthesized by Sigma-Aldrich with phosphorothioate modifications at the three terminal bases on both the 5’- and 3’-ends to enhance stability and prevent nuclease degradation.

### 2.6 *In-vitro* treatment of stigma and pollination assay

For the *in vitro* oligonucleotide treatment, the top-dripping method previously described was used (Zhang et al., 2024). Briefly, in this method, flowers were placed on Pollen Germination Media (PGM) with agar (5 mM CaCl_2_, 5 mM KCl, 0.01% H_3_BO_3_, 1 mM Mg_2_SO4·7H_2_O, 10% sucrose, and 0.8% agarose at pH 7.5). Further, 2 µL of 30 µM S-ODN, AS-ODN (with 0.0125% Tween 20), or mock (nuclease-free water with 0.0125% Tween 20) was pipetted onto each stigma. Following treatment, stigmas were placed at 22 °C with 70% relative humidity for 1.5 h.

These treated stigmas were manually pollinated with self- or cross-pollen and incubated for an additional 12 h under the same conditions as previously described, followed by aniline blue staining to assess pollen attachment and pollen tube count.

### 2.7 Aniline blue staining

Stigmas were kept in Carnoy’s Fixative (acetic acid: ethanol = 1:3) for decolorization for 2 h. Samples were then transferred to 1 M NaOH at 55 °C for 25 min to soften the tissue. Next, the stigmas were stained with aniline blue (0.1% in 108 mM Potassium phosphate tribasic buffer - K_3_PO_4_, pH 11) for 2 h, and pollen grains and tubes were visualized with a Leica epifluorescence microscope and captured with a KI3C digital camera.

### 2.8 Nitro blue tetrazolium assay for ROS estimation

For NBT staining, Mock-, Sense-, or Antisense ODN-treated stigmas were stained as previously described (Sankaranarayanan et al., 2025). Briefly, stigmas were collected at unpollinated or 15, 30, and 60 minutes after self-incompatible pollination, and were incubated in NBT staining solution (1mg/ml in 200 mM phosphate buffer, pH 7, and 0.02% Silwet) for 2.5 hours. Samples were then transferred to an ethanol: glycerol: acetic acid solution (3:1:1) and heated at 90 °C for 10 minutes to clear the tissues. Finally, the stigmas were mounted in chloral hydrate: glycerol: water (8:1:2) for further clearing and visualized under a Leica microscope with a brightfield setup.

### 2.9 Reverse-transcription PCR

For RT-PCR, primers were designed using Primer3Plus (Untergasser et al., 2012) with default settings (**Supplementary Table 9**). Primer specificity was assessed using BLAST against *Brassica rapa* Chiifu V 4.0 genome in the BRAD database (Chen et al., 2022). For expression studies, stigmas were either left unpollinated or pollinated for 30 or 60 minutes. The tissue was then used for RNA isolation with the RNeasy Plant Mini Kit (Qiagen, Catalog: 74904) according to the manufacturer’s protocol. An equal amount of RNA was used to synthesize cDNA using the Thermo Scientific First Strand cDNA synthesis kit (Catalog: K1612). The cDNA was used in an RT-PCR reaction with 2X Takara Master Mix (Catalog: RR310A). All RT-PCR reactions were repeated 3 times, and *BrActin* 7 (NCBI Accession: KU851921) was used as a control. For RT-PCR of ODN-treated stigmas, stigmas were treated with oligonucleotides as previously described for 1.5 h. After treatment, stigmas were used for RT-PCR as previously described.

### 2.10 Data analysis

For the cross-compatibility assay, seeds were counted from 5 pods each, with three biological replicates (n=15) (**Supplementary Data 2**), and pollen attachment and tube penetration were measured in 5 independent replicates (n=5) (**Supplementary Data 2**). To determine seed diameter and area, measurements were performed on n=5 replicates using the Straight Line and Freehand Selection tools in ImageJ (Schneider et al., 2012) (**Supplementary Data 2**). For the AS-ODN-based assay, 3 stigmas from each of 3 biological replicates (n = 9) were analyzed per treatment for pollen attachment and pollen tube penetration (**Supplementary Data 2**). To quantify the intensities of the Nitro blue tetrazolium (NBT) assay, fluorescent intensities were randomly measured across the stigmatic papillary cells using a 120x120 μm region of interest (ROI) in ImageJ (**Supplementary Data 2**) (Schneider et al., 2012). Further, these gray values were reverse-normalized, as greater NBT staining yields lower gray values. For RT-PCR analysis, the band intensities were calculated using ImageJ (Schneider et al., 2012) (**Supplementary Data 2**).

Graphs were plotted using GraphPad Prism 8.0.2 (www.graphpad.com). One-way ANOVA (P < 0.05) was used to calculate statistics, p < 0.05, * indicates a significant difference, and ns indicates no significant difference.

## 3 Results

### 3.1 Phenotypic and cross-compatibility characterization of *Brassica rapa* self-incompatible toria and self-compatible yellow sarson

To investigate self-incompatibility in *Brassica rapa* L., we grew two commercially available varieties, toria (self-incompatible) and yellow sarson (compatible). The plants were optimized for growth, and germination occurred within 7 days post-plating, while toria flowered around 30 days, whereas yellow sarson flowered approximately 35–40 days post-germination (**Supplementary Figure 1A**). The flowers were cruciform and yellow in color, comprising all the four whorls - sepals (four, narrow, spreading, yellow green and about 5–8 mm long), petals (four, obovate, bright yellow, arranged in a cross with a slender claw and about 6–11 mm long), stamens (tetradynamous arrangement - 4 long and 2 short), and pistil (superior elongated ovary, with a short style and capitate stigma) (**Supplementary Figure 1B**). Floral development from the bud stage to flowering took about 48 hours, with stage 1 as the bud stage, stage 2 as a slightly opened flower, and stage 3 as a fully opened flower, with the highest receptivity to pollination at stage 2, as observed in various crosses conducted in this study (**Supplementary Figure 1C**).

**Figure 1:**
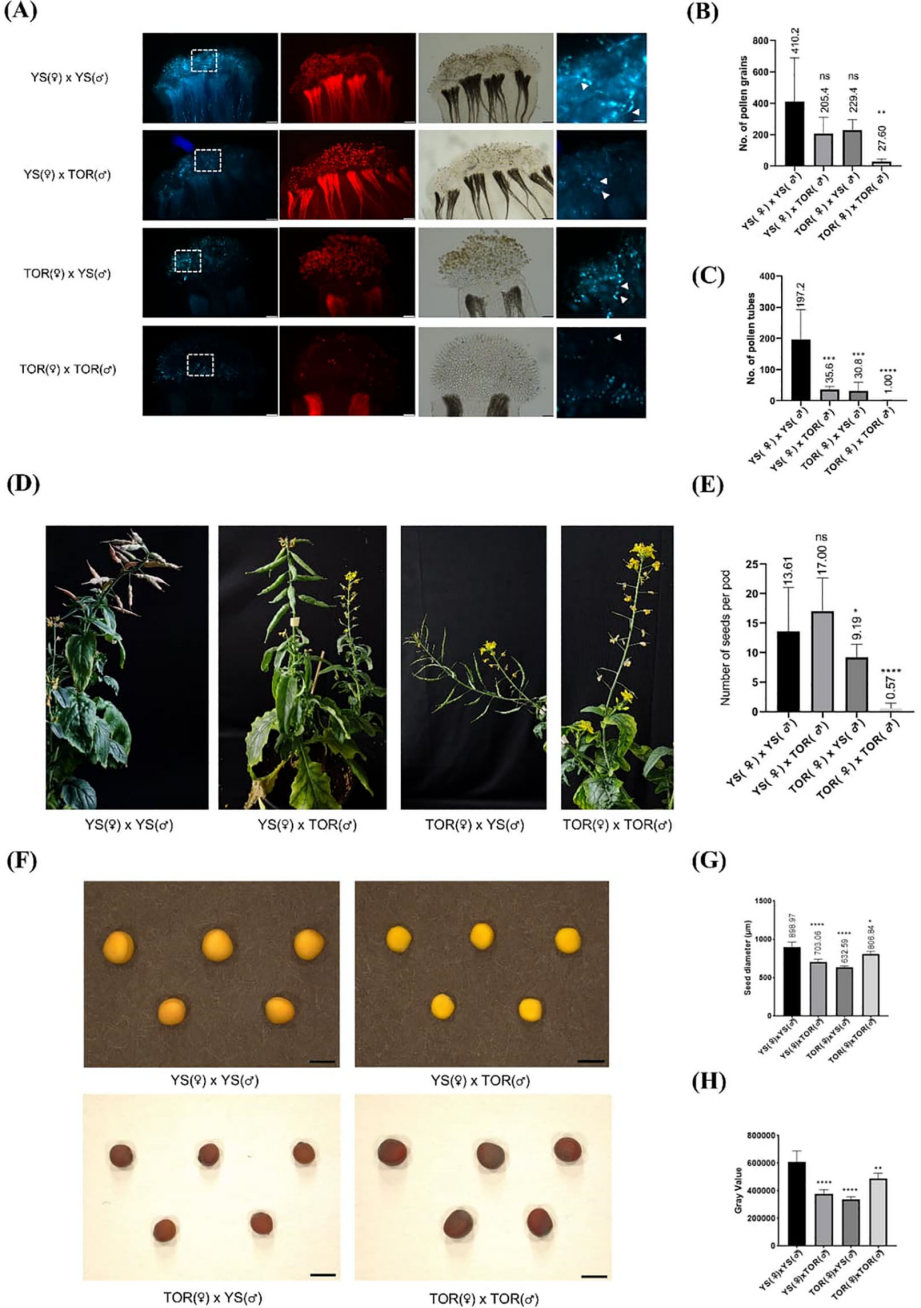
Cross compatibility between *B.rapa* var. toria and yellow sarson. **(A)** Representative images of aniline blue assay indicating the cross-compatibility between *B.rapa* var. toria and yellow sarson. Note: Aniline blue (first), Red channel (second), Bright field (third), Zoomed (fourth). Note: Scale bars: 200 μm & 50 μm (zoomed), white arrows indicate pollen grains with emerging pollen tubes. **(B)** Graph representing the number of pollen grains in various crosses between *B.rapa* var. toria and yellow sarson. Note: One way ANOVA, *P < 0.05, n = 5, ns = not significant. **(C)** Graph representing the number of pollen tubes in various crosses between *B.rapa* var. toria and yellow sarson. Note: One way ANOVA, *P < 0.05, n = 5, ns = not significant. **(D)** Mature plants showing pods from various *in-planta* crosses (seed-set assay) between *B.rapa* var. toria and yellow sarson. **(E)** Graph representing the number of seeds per pod in various crosses between *B.rapa* var. toria and yellow sarson. Note: the values over the scale bar indicate average seeds per pod, One way ANOVA, *P < 0.05, n = 15, ns = not significant. **(F)** Morphology of seeds collected from various crosses between *B.rapa* var. toria and yellow sarson. Scale bar: 750 µm. **(G)** Graph representing seed diameter from various crosses between *B.rapa* var. toria and yellow sarson. Average seed diameter is indicated above the bars, One way ANOVA, *P < 0.05, n = 5. **(H)** Graph representing gray values (seed area) from various crosses between *B.rapa* var. toria and yellow sarson. Note: One way ANOVA, *P < 0.05, n = 5.

Next, we examined cross-compatibility between the two varieties, which is crucial for any system used to dissect and manipulate self-incompatibility. Flowers were emasculated at stage 1 (**Supplementary Figure 1C**) and subsequently pollinated with pollen from both self- and cross- compatible donors (**Figures 1A–C**). Aniline blue assay revealed that pollen attachment was comparable in both cross-pollinations with compatible pollination, while self-pollinated toria showed negligible pollen attachment. Furthermore, the number of pollen tubes penetrating the style was markedly reduced in both cross-pollinations relative to the compatible control (**Figures 1A–C**). Seed set assays showed that the cross with yellow sarson as the female parent and toria as the pollen donor produced a seed number similar to the compatible control, whereas the reciprocal combination yielded slightly fewer seeds (**Figures 1D, E**). Seeds from compatible and yellow sarson (♀) × toria (♂) crosses were yellow, whereas seeds from incompatible and toria (♀) × yellow sarson (♂) crosses were brown (**Figure 1F**). Quantitative analysis of seed diameter and area indicated smaller sizes for both crosses compared to self-pollinated seeds (**Figures 1F–H**) (**Supplementary Data 2**). Furthermore, the F1 generation seeds showed approximately 100% germination, further supplementing the cross-compatibility trait (**Supplementary Figure 2**, **Supplementary Data 2**).

### 3.2 Conservation and phylogenetic characterization of self-incompatibility regulators in *Brassica rapa*

To investigate the role of key self-incompatibility associated genes *SRK*, *FER1*, *MLPK*, and *ARC1* in Brassicaceae (**Supplementary Table 3-6**) (Abhinandan et al., 2023; Bhalla et al., 2025a), we amplified them using previously known sequences. The bands obtained were of expected sizes with no non-specific amplification except *MLPK* (**Supplementary Figure 3A**). These bands showed positive colonies and expected band sizes in the colony PCR and restriction digestion-based analysis (**Supplementary Figures 3B, C**). Sanger sequencing provided the requisite sequences for these genes, which were further analyzed for sequential analysis with previously known candidates and submitted to NCBI (**Supplementary Data 1**).

**Figure 2:**
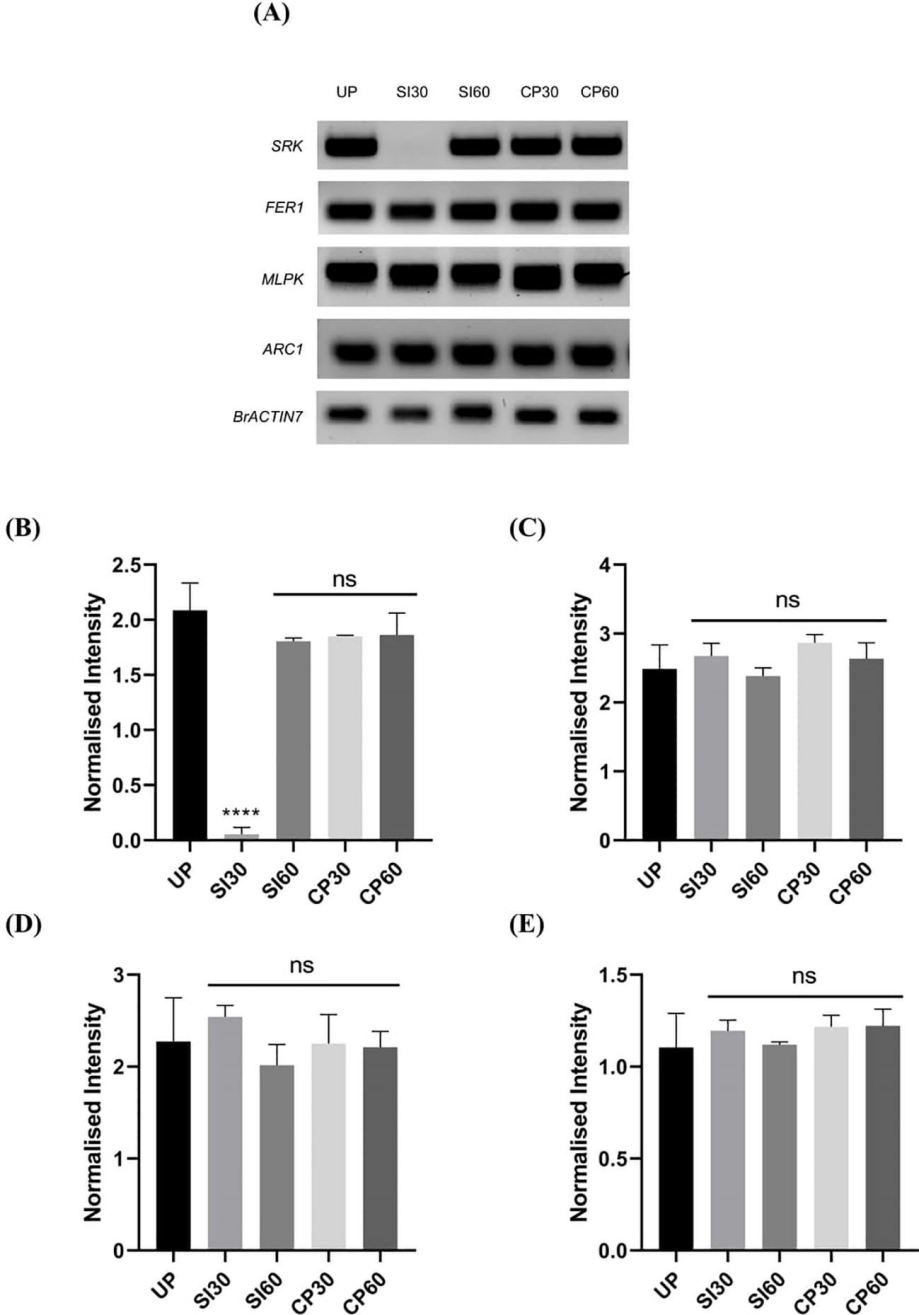
Expression analysis of candidate genes in stigmas. **(A)** Representative gel image for expression of *SRK*, *FER1*, *MLPK*, and *ARC1* in unpollinated (UP), self-incompatible (SI30 and SI60–30 and 60 MAP), and compatible (CP30 and CP60–30 and 60 MAP) pollinated stigmas. Note: *BrActin 7* (NCBI Accession: KU851921) was used as an internal control. Control images are reused for illustrative purposes. **(B–E)** Graph representing the normalized intensity for expression of *SRK* **(B)**, *FER1* **(C)**, *MLPK* **(D)**, and *ARC1* **(E)** in unpollinated (UP), self-incompatible (SI30 and SI60–30 and 60 MAP), and compatible (CP30 and CP60–30 and 60 MAP) pollinated stigmas. *BrActin 7* (NCBI Accession: KU851921) was used for normalization and as an internal control, one-way ANOVA, *P < 0.05, n = 3, ns, not significant.

To analyze their expression dynamics across various time points after an incompatible or compatible response, we performed RT-PCR analysis on stigmatic tissue. For *SRK*, we identified a decrease at 30 minutes after pollination (MAP) during the incompatible response (SI30), followed by an increase at 60 MAP (SI60) to levels equivalent to those in the unpollinated state (**Figures 2A, B**). For *FER1*, *MLPK*, and *ARC1*, the expression pattern was stable and didn’t show any differential expression across various pollination time-points (**Figure 2**, **Supplementary Figure 4**).

**Figure 3:**
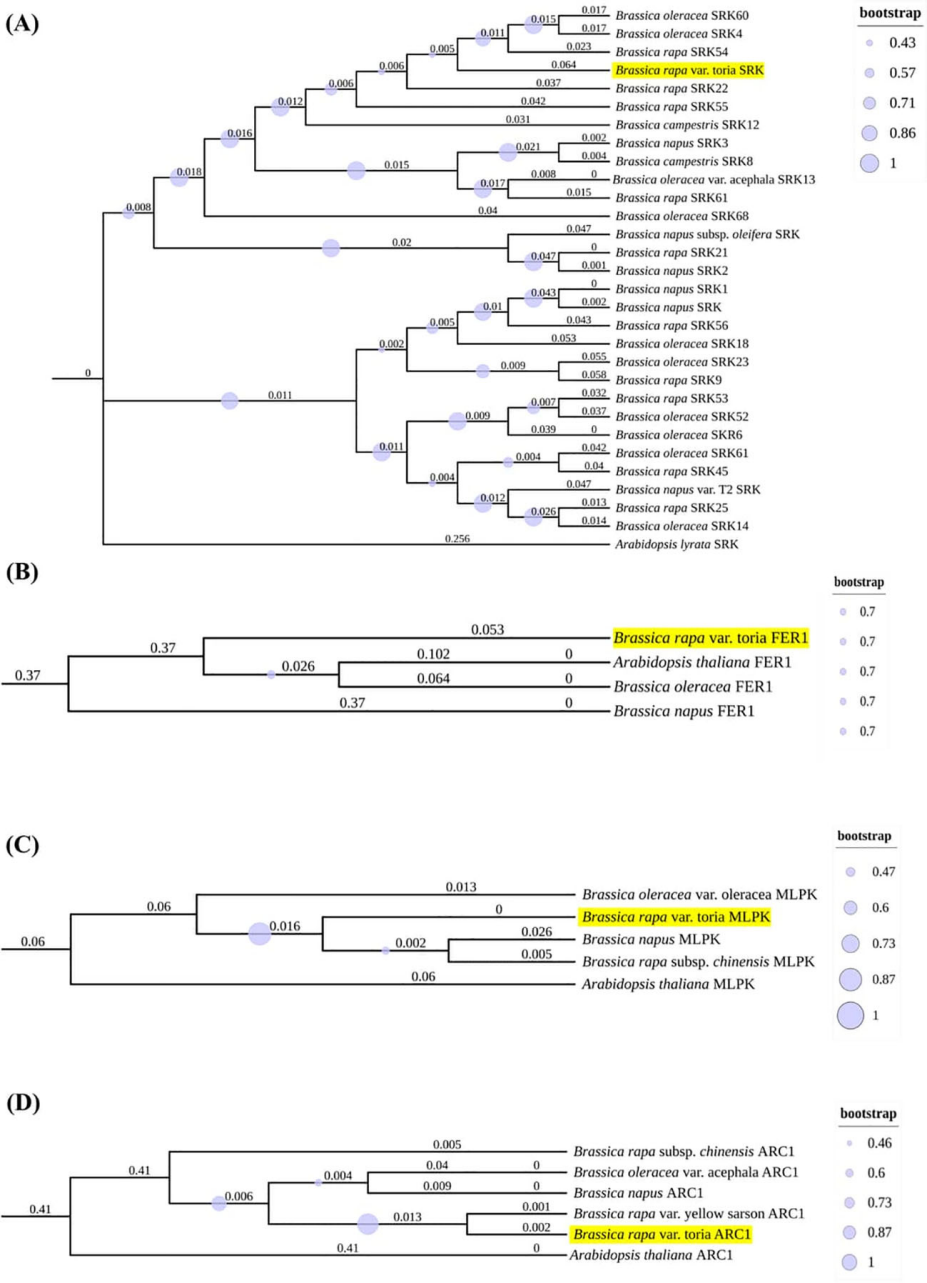
Phylogenetic analysis of genes under study. Phylogenetic tree of **(A)** *SRK*, **(B)** *FER1*, **(C)** *MLPK*, and **(D)** *ARC1* genes from *Brassica* spp. and *Arabidopsis* (**Supplementary Table 3-6**) inferred from multiple sequence alignment (Supplementary Data 3), using the maximum likelihood method. Note: Bootstraps: 1000 replicates.

To better understand their evolutionary relationships with previously known candidates, a comparison with homologous sequences was performed (**Supplementary Table 3-6**). Multiple sequence alignments (**Supplementary Data 3**) and phylogenetic trees (**Figure 3**) for *SRK*, *FER1*, *MLPK*, and *ARC1* indicated broad conservation across species within the U’s triangle, with *Arabidopsis thaliana* and *Arabidopsis lyrata* as outgroups. The largest number of homologs was obtained for *SRK*. In general, two distinct clades were evident, with *Brassica rapa* var. toria *SRK* clustering closely with *BrSRK22* and showing strong bootstrap support (**Figure 3A**). *FER1* yielded only four sequences above the identity cutoff, yet it grouped more closely with *AtFER1* (**Figure 3B**). *MLPK* showed greater relatedness to *BrMLPK* from *B. rapa* subsp. *chinensis* and *Brassica napus* (**Figure 3C**), whereas *ARC1* exhibited higher similarity to *BrARC1* from yellow sarson (**Figure 3D**). Together, these analyses indicated that their functional importance is strongly reinforced in the self-incompatibility response.

### 3.3 Structure-function, and *in-silico* characterization of self-incompatibility regulators in *Brassica rapa*

To understand the functional properties of SRK, FER1, MLPK, and ARC1, it is essential to examine their structural organization, which provides key insights into their structure-function relationships and functional integrity in this two-variety system. We used various *in silico* tools for secondary and tertiary structure prediction, analysis of prediction accuracy, and domain identification.

**Figure 4:**
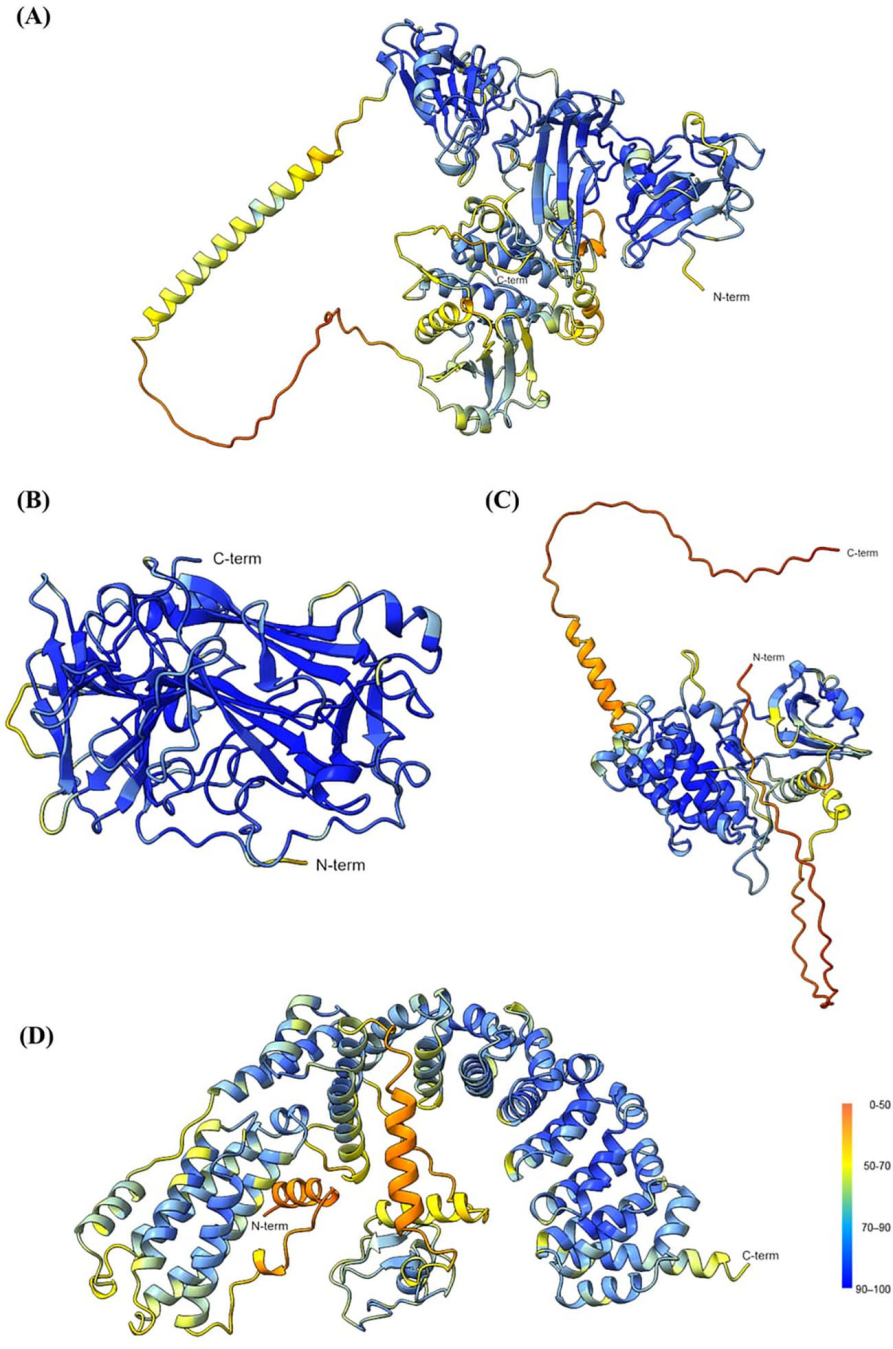
Structure prediction of various proteins regulating self-incompatibility in *Brassica rapa*. Three-dimensional (3-D) structure predictions of **(A)** SRK, **(B)** FER1, **(C)** MLPK, and **(D)** ARC1 proteins using AlphaFold3. The structures are colored as per pLDDT scores. Dark blue color indicates very high accuracy (pLDDT >90), light blue indicates high confidence (90 > pLDDT > 70), yellow orange indicates low confidence (70 > pLDDT > 50), and red represents very low confidence (pLDDT < 50).

SRK, 829 amino acids long (**Supplementary Data 1**), revealed helical and coiled structures at the C-terminal serine/threonine kinase domain, while the N-terminal showed β-strands (**Supplementary Figure 6A**, **Supplementary Table 7**). The 3D structure reciprocated the 2D structure prediction with a high pTM (0.6) and pLDDT score (**Figure 4A**). The predicted 3D structure revealed 89.8% residues in the favored region of the Ramachandran plot, indicating high confidence (**Supplementary Figure 7A**). The 3D structure and CDD analysis revealed two N-terminal mannose-binding lectin-type domains, an S-locus glycoprotein domain, and a C-terminal PAN domain (**Figure 4A** and **Supplementary Figure 5A**).

Next, for FER1, 896 amino acids long (**Supplementary Data 1**), indicated a C-terminal helix and β-strands for the extracellular malectin-like domain, while the N-terminal had predominance of helix and coils representing the cytosolic kinase domain (**Supplementary Figure 5B**, **Supplementary Table 7**). The 3D structure showed a high pTM (0.96) and pLDDT score. Further, 92.4% of residues were found to be in the favored region, indicating the quality of the predicted structure (**Supplementary Figure 7B**). 3D structure and CDD analysis revealed the presence of an extracellular malectin-like domain, a transmembrane domain, and a serine/threonine kinase domain (**Figure 4B**; **Supplementary Figure 5B**; **Supplementary Table 7**).

For MLPK, a 418-residue-long protein (**Supplementary Data 1**), higher helical content was predicted, with few β-strands in both the N-terminal catalytic domain and the C-terminal kinase domain (**Supplementary Figure 6C**, **Supplementary Table 7**). The 3D structure, with a pTM score of 0.77 and a high pLDDT score, and CDD analysis revealed the N-terminal catalytic domain and the C-terminal kinase domain (**Figure 4C**; **Supplementary Figure 5C**; **Supplementary Table 7**). The 3D structure showed 91.4% of residues in the favored region, further supporting the predicted structure (**Supplementary Figure 7C**).

At last, ARC1, 661 amino acids long (**Supplementary Data 1**), showed predominantly random coils and helices at the N-terminal (**Supplementary Figure 6D**, **Supplementary Table 7**). The 3D structure, with a pTM score of 0.72, a high pLDDT score, and 95.7% residues in the favored region, indicated good-quality prediction (**Figure 4D**, **Supplementary Figure 7D**). The CDD analysis and 3D prediction revealed the presence of the N-terminal U-Box domain and C-terminal armadillo (ARM) repeat-containing domain (**Figure 4D**; **Supplementary Figure 5D**; **Supplementary Table 7**).

### 3.4 Functional characterization of genes responsible for SI response

**Figure 5:**
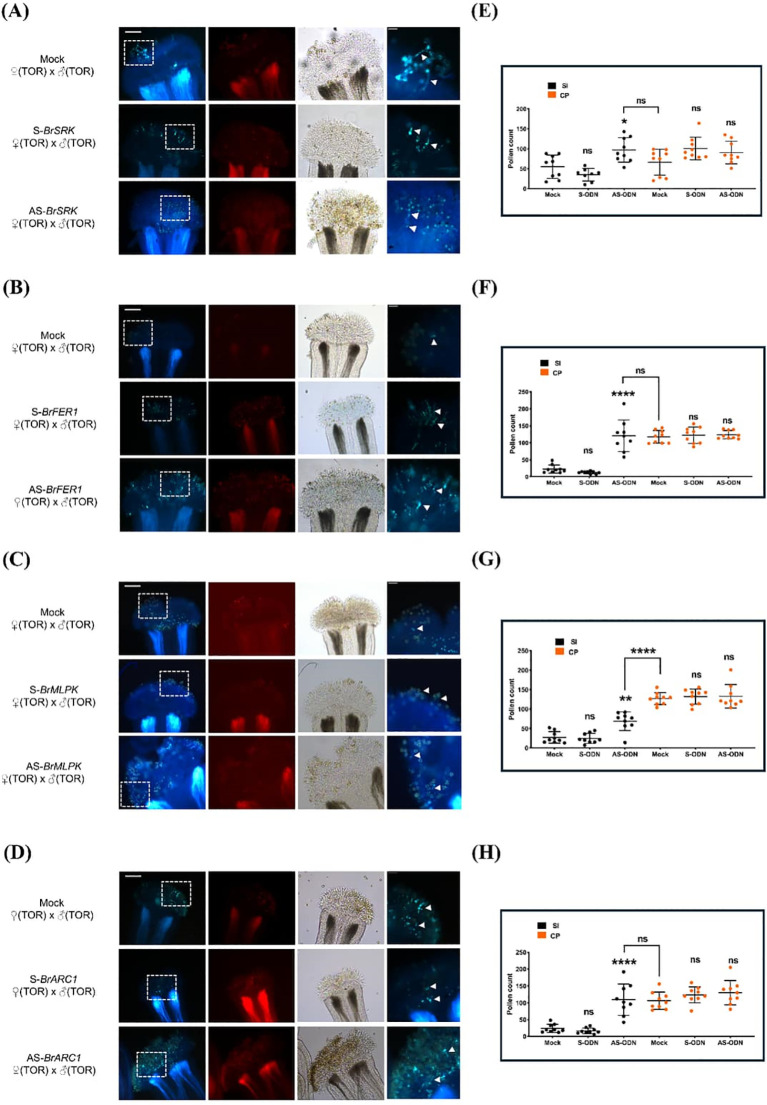
Functional characterization of genes during self-incompatibility response. **(A–D)** Representative aniline blue images of pollinated stigmas treated with Mock, Sense, and Anti-Sense ODNs of **(A)** *SRK*, **(B)** *FER1*, **(C)** *MLPK*, and **(D)** *ARC1*, showing pollen attachment and tube formation. Note: Aniline blue (first), Red channel (second), Bright field (third), Zoomed (fourth). Note: Scale bars: 300 μm & 50 μm (zoomed), white arrows indicate pollen grains with emerging pollen tubes. **(E–H)** Graph representing the number of pollen grains per stigma observed in (**Figures 5A–D**); (**Supplementary Figures 9A–D**) treated with Mock, Sense, and Anti-Sense ODNs of **(E)** *SRK*, **(F)** *FER1*, **(G)** *MLPK*, and **(H)** *ARC1*. Note: Dots represent individual data points. One way ANOVA, *P < 0.05, n = 9, ns, not significant.

To functionally validate the roles of *SRK*, *FER1*, *MLPK*, and *ARC1* in the self-incompatibility (SI) response of *Brassica rapa* var. toria, we suppressed these genes using antisense oligonucleotides (AS-ODNs). Based on previous studies, we adopted the top-dripping method (**Supplementary Figure 8**, **Supplementary Table 8**). Following treatment, stigmas were pollinated with either self- or cross-compatible pollen. In self-pollinated toria stigmas, AS-ODN treatment significantly increased pollen attachment for all four genes compared with mock and sense controls (**Figure 5**, **Supplementary Figure 9**). In contrast, under cross-pollination conditions, where pollen acceptance and tube growth occur naturally in the absence of SI, AS-ODN treatments had no significant effect on pollen attachment or tube penetration (**Figure 5**, **Supplementary Figure 9**). The number of pollen tubes penetrating the style likewise increased in AS-ODN-treated-self-incompatible stigmas relative to mock and sense treatments for *SRK*, *FER1*, and *ARC1*, whereas AS-*MLPK*-treated stigmas showed no significant increase in pollen tube penetration (**Figure 5**, **Supplementary Figure 9**).

**Figure 6:**
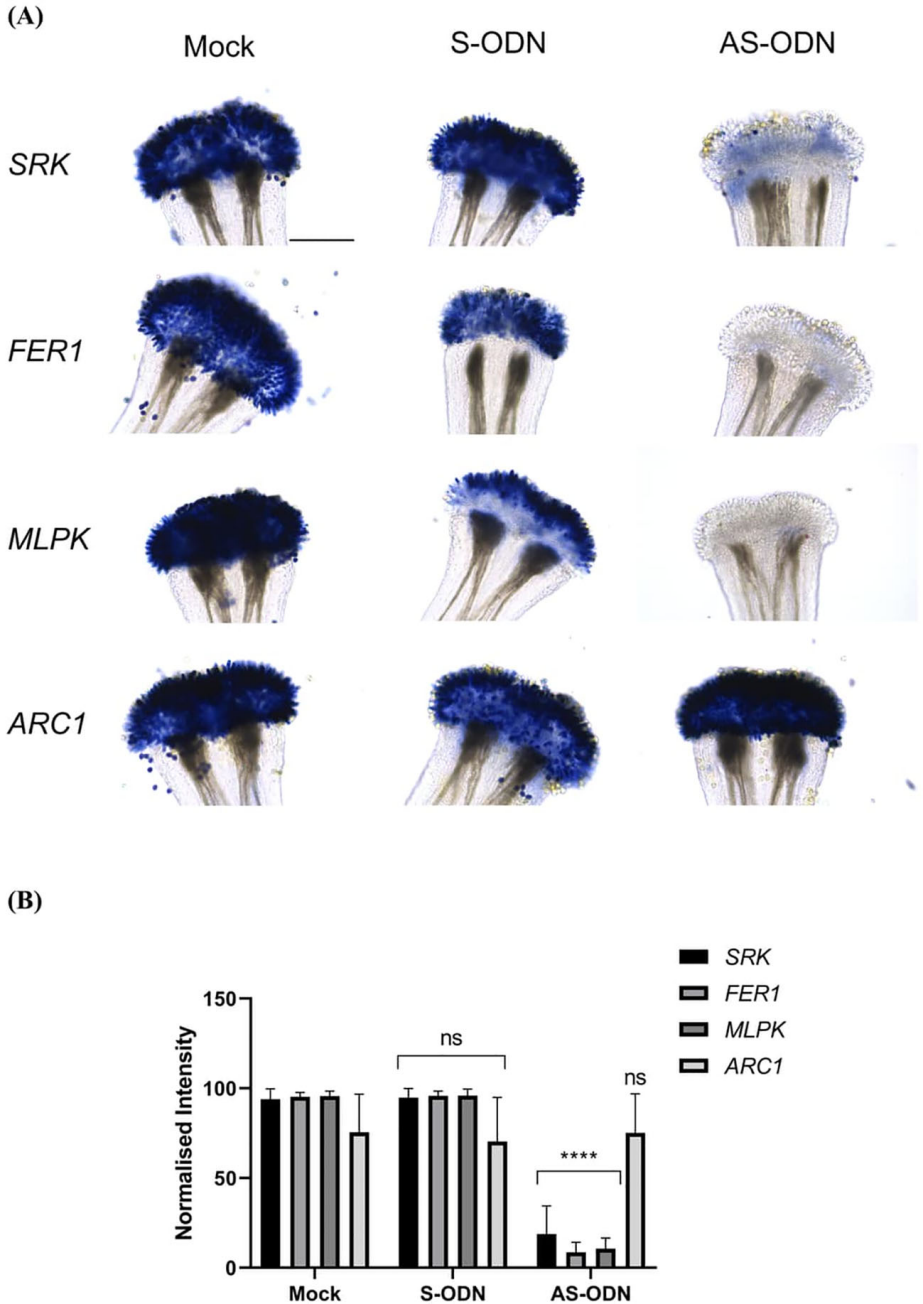
NBT staining assay for genes responsible for regulating self-incompatibility. **(A)** Representative images for various stigmas after 30 minutes of pollination, treated with Mock, Sense, and Anti-Sense ODNs for various genes under study, and stained using NBT. Scale bar: 300 µm **(B)** Graph representing the reverse normalized intensity for various stigmas, pollinated for 30 minutes, treated with Mock, Sense, and Anti-Sense ODNs for various genes under study, and stained with NBT. Note: Higher intensity indicates more NBT staining and ROS levels, One-way ANOVA, *p<0.05, n=15.

Next, to evaluate the impact of ODN-mediated gene suppression on reactive oxygen species (ROS) levels, which are highly dynamic during the SI response, particularly superoxide, we performed NBT staining on pollinated stigmas. We first optimized the time window for ROS analysis by monitoring ROS dynamics in unpollinated stigmas and at 15, 30, and 60 minutes after pollination (MAP) (**Supplementary Figure 10**). ROS levels rose at 15 and 30 MAP and then declined at 60 MAP, indicating a peak at 30 MAP (**Supplementary Figure 10**). Using this optimized time point, we assessed the effects of mock, S-ODN, and AS-ODN treatments on ROS accumulation in stigmas at 30 MAP (**Figure 6**). ROS levels were significantly reduced after AS-ODN treatment for *SRK*, *FER1*, and *MLPK*, whereas *ARC1* knockdown had no detectable effect on ROS (**Figure 6**).

**Figure 7:**
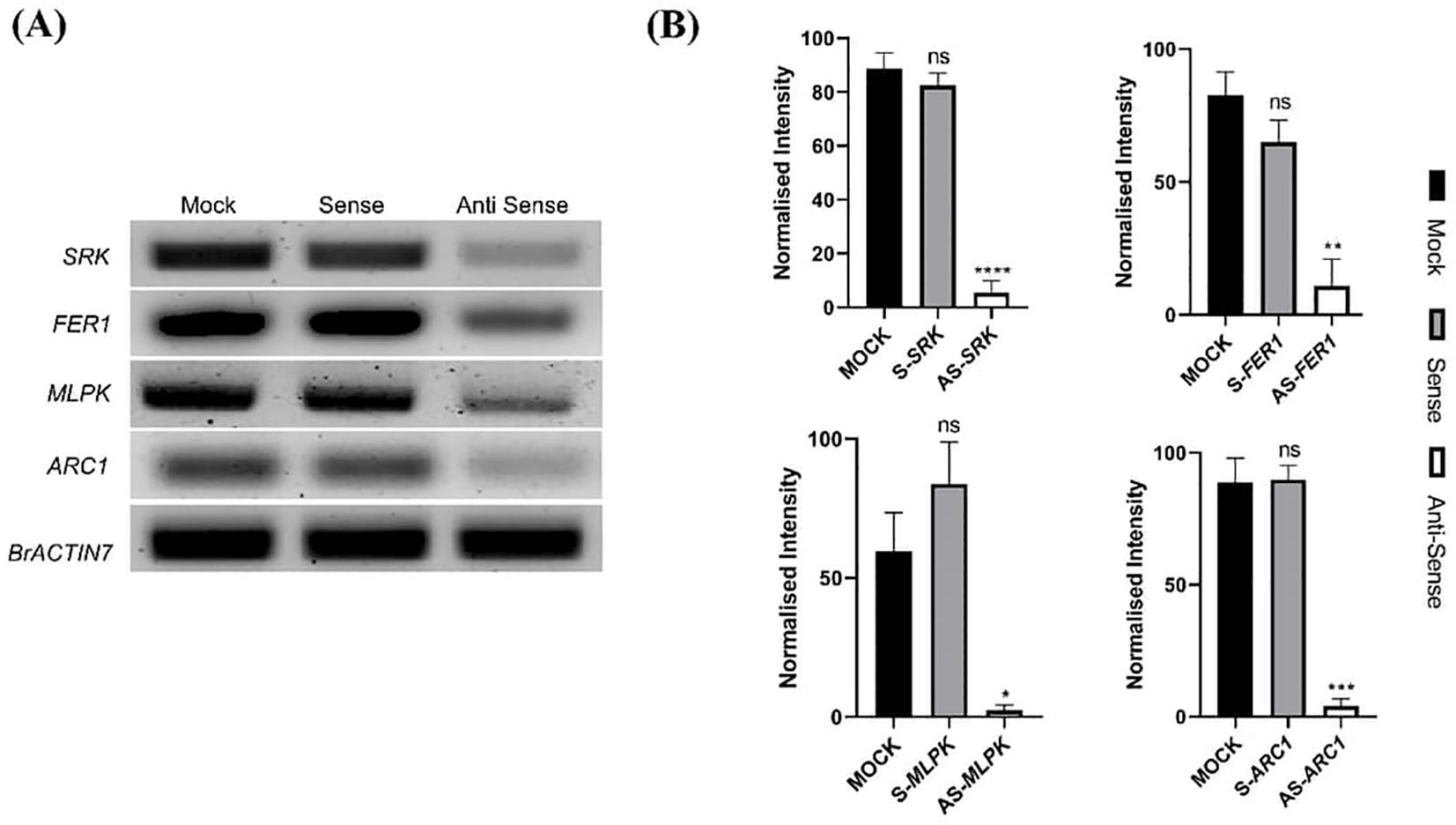
Expression analysis of ODN-treated stigmas. **(A)** Representative gel image for the *SRK*, *FER1*, *MLPK*, and *ARC1* treated with ODNs - Mock, Sense, and Anti-Sense. *BrActin 7* (NCBI Accession: KU851921) was used as an internal control. Control images are reused for illustrative purposes. **(B)** Graph representing the normalized intensity for various bands of *SRK*, *FER1*, *MLPK*, and *ARC1* treated with ODNs - Mock, Sense, and Anti-Sense. *BrActin 7* (NCBI Accession: KU851921) was used for normalization and as an internal control, one-way ANOVA, *P < 0.05, n = 3, ns, not significant.

To confirm that ODN treatment indeed downregulated target gene expression, we performed RT-PCR on stigma tissue following mock, sense, and anti-sense ODN treatments. Gene-specific primers together with the reference gene *BrActin7* (NCBI Accession: KU851921) (**Supplementary Table 9**) were used for amplification. The RT-PCR analysis revealed a clear reduction in transcript levels of all four target genes in AS-ODN-treated stigmas, while expression levels remained comparable between mock and sense-treated controls (**Figure 7**; **Supplementary Figures 11, 12**).

## 4 Discussion

Understanding self-incompatibility (SI) systems and exploiting them for crop improvement, hybrid breeding, and enhancement of genetic diversity is of central importance and has been successfully applied in several crops worldwide. The Brassicaceae family, encompassing economically vital vegetable, oilseed, and condiment species, has become a key model for dissecting incompatibility mechanisms and developing strategies to introduce traits such as high yield, disease resistance, and shatter tolerance. Despite this, the application of SI-based approaches in *Brassica rapa* L. remains limited, even though the species is well adapted to both irrigated and rainfed environments, amenable to sole or mixed cropping, and profitable at relatively low input and water requirements. Under these conditions, characterizing and harnessing the SI system for integrating major agronomic traits becomes particularly imperative.

Our study investigates the SI trait in *Brassica rapa* L., focusing on two commercially available and widely cultivated varieties: toria and yellow sarson. Toria is a self-incompatible, early-maturing form selected under breeding pressure, whereas yellow sarson represents a quality-type mutant selected for seed and oil characteristics (Thakur et al., 2017). Building on prior work, we optimized growth conditions for these varieties and report detailed observations on their morphology and reproductive development, within the context of elucidating self-incompatibility in *Brassica rapa*. We further evaluated cross-compatibility between the two varieties, a prerequisite for utilizing SI in breeding schemes. Although the self-incompatible variety shows partial resistance to pollen tube penetration and seed set, it still permits sufficient fertilization and seed production to be integrated into hybrid-breeding programs.

The seed color and size differences observed in these crosses provide simple but informative phenotypic signatures of the underlying genetic behavior and physiological interactions. Uniformly yellow seeds from compatible crosses and from the yellow sarson (♀) × toria (♂) cross, in contrast to brown seeds from incompatible and toria (♀) × yellow sarson (♂) crosses, suggest a non-Mendelian, parent-of-origin effect more consistent with maternal or cytoplasmic control than with a simple nuclear dominant/recessive system. The reduced seed size in both yellow sarson (♀) × toria (♂) and toria (♀) × yellow sarson (♂) crosses, compared with fully compatible combinations, further indicates that the incompatibility status and the direction of the cross modulate resource allocation and seed development, likely reflecting disturbed pollen tube guidance, fertilization efficiency, or early post-fertilization interactions.

Furthermore, in this study, we sequenced all the major genes previously known to regulate self-incompatibility in these two varieties, which were submitted to NCBI (**Supplementary Table 1**) (Sayers et al., 2022) and provided insights into their conservation and relatedness to other species in the *Brassica* genus. Expression studies showed no significant differential expression of *FER1*, *MLPK*, and *ARC1* between incompatible and compatible responses, indicating proteomic-level regulation of the incompatibility response, as previously reported (Zhang et al., 2017). Further, for *SRK*, the decrease at 30 minutes after incompatible pollination indicates a probable gradual feedback decline after activation and a shift to post-translational regulation of the SI response, consistent with previous reports (Ivanov and Gaude, 2009). Further, the increase at 60 minutes after incompatible pollination can be attributed to the reinstatement of new SRK molecules at the plasma membrane, thereby ensuring incompatibility with the upcoming self-pollen. Previous reports suggest that SRK is degraded after activation and that *de novo* protein synthesis compensates around 60 minutes after pollination (Ivanov and Gaude, 2009), a timing that coincides with the reported transcriptional data. This stable expression, along with the absence of a post-pollination spike in both incompatible and compatible responses, is consistent with previous reports (Ivanov and Gaude, 2009; Zhang et al., 2017). Phylogenetic analysis of the newly characterized *Brassica rapa* var. toria sequences for *SRK*, *FER1*, *MLPK*, and *ARC1*, together with homologues from species of the U’s triangle and *Arabidopsis* as an outgroup, revealed close evolutionary relationships and shared ancestral origins. The largest number of homologs obtained for *SRK* indicates consistency with its known allelic and haplotype diversity in *SP11*/*SCR*-mediated SI (Abhinandan et al., 2022). Furthermore, the proximity of *BrARC1* from varieties toria and yellow sarson underscores conservation between varieties. By examining these genes in *B. rapa* var. toria, we highlight their conservation both within the U’s triangle species and across closely related Brassicaceae, underscoring their functional relevance in SI.

Understanding a protein’s structural details, three-dimensional conformation, and catalytic domains is essential for interpreting its functional role in the biological system. *In-silico* analyses using PSIPRED and AlphaFold3 predicted the secondary and tertiary structures of SRK, FER1, MLPK, and ARC1, while conserved-domain searches identified motifs characteristic of their roles in *Brassica* SI. The structural similarity between our SRK model and the experimentally resolved SRK9:SCR9 complex, particularly the presence of N-terminal α-helices, lectin-type domains, an S locus glycoprotein domain, and a PAN-like domain, also highlights the mechanistic basis of SRK-mediated self-pollen rejection in our variety under study. Given the structural organization and the homology of domains in SRK, especially the S-glycoprotein domain implicated in SI previously, and conserved cysteine residues, which contribute to structural stability and functional relevance, indicate functionally active SRK in toria SI effect (Naithani et al., 2007; Ma et al., 2016). Likewise, *in-silico* analysis of FER1 identified an extracellular malectin-like receptor domain involved in pollen selection, a transmembrane region with a hydrophobic segment, and a cytosolic serine/threonine kinase domain that mediates downstream ROS signaling (Cheng et al., 2025). The predicted extracellular domain structure closely resembles the topology of the *At*FER1 crystal structure, supporting functional conservation across Brassicaceae (Xiao et al., 2019; Cheung, 2024; Bhalla et al., 2025b). Detailed structural studies of the cytosolic domain of FER1 in this family will further clarify its role in the signaling cascade. Given the presence and homology of these domains, and their structural alignment with the previous literature and known structures, we suspect that FER is functionally relevant to toria incompatibility. MLPK has previously been shown to bind SRK and phosphorylate downstream effectors to reinforce the rejection of self-pollen (Kakita et al., 2007), and functional analyses in *Brassica napus*, a functional analogue of *Brassica rapa* MLPK, have demonstrated its positive regulation of SI by modulating expression of *SRK* and *ARC1* (Chen et al., 2019). Our *in-silico* model of MLPK revealed conserved serine/threonine kinase, ATP-binding, and substrate-binding sites, consistent with a membrane-anchored kinase function. Although the crystal structure has not been resolved previously, homology based on conserved domains and the confidence in the prediction provide insights into its functional relevance. Although no crystal structure has been previously elucidated, the ARC1 structural model shows a conserved N-terminal ubiquitin ligase domain responsible for ubiquitin ligase activity, and a C-terminal armadillo (ARM) repeat-containing domain regulating substrate specificity, supporting earlier reports of its E3 ligase activity and a positive role in SI for the variety under study (Stone et al., 2003; Andersen et al., 2004). Given this *in silico* analysis, it highlights homology with previously known candidates, a conserved domain architecture, and functional relevance for contributions to SI and reproductive responses. Furthermore, these structures, determined by crystallography or other methods, would deepen understanding of their mechanistic roles in the Brassicaceae SI pathway.

Further, ODN-based transient suppression has previously been utilized and characterized in Brassicaceae, which bind to target mRNAs and thereby inhibit gene expression (Huang et al., 2020; Zhang et al., 2024). Using the previously optimized technique, we suppressed major self-incompatibility genes, including *SRK*, *FER1*, *MLPK*, and *ARC1*. AS-ODN treatment increased pollen attachment and tube penetration, resulting in complete breakdown of SI for three genes, except *MLPK*, whose suppression showed modest pollen attachment relative to the compatible control. This breakdown underscores the functional importance of these genes in regulating the SI response in *Brassica rapa*. MLPK, although being shown as essential in certain studies (Murase et al., 2004; Chen et al., 2019), has also been identified as non-essential in others (Murase et al., 2004; Kakita et al., 2008; Kitashiba et al., 2011; Ohata et al., 2023). Interestingly, MLPK’s mutation has previously been reported as the cause of compatibility in yellow sarson, among other players (Murase et al., 2004; Fujimoto et al., 2006). Furthermore, AS-ODN treatment did not alter compatible pollination, indicating that the previously optimized concentration and time point are also suitable for *Brassica rapa* L (Zhang et al., 2024). In alignment with previous studies, AS-ODN treatment of genes *SRK*, *FER1*, and *MLPK* inhibited ROS bursts at 30 MAP, whereas *ARC1* AS-ODN did not affect ROS levels (Huang et al., 2023), indicating the parallel nature of the ARC1- and FER-mediated pathways as well as the interactions between SRK, MLPK, and FER1 directly or indirectly with the ROS machinery. Previous studies indicating that ARC1 functions as an independent pathway for SI regulation, distinct from the FER-mediated ROS pathway, are supported by our results in *Brassica rapa* (Huang et al., 2023). AS-*ARC1* treatment has previously been shown not to affect ROS levels during the SI response, which holds true for the varieties under study (Huang et al., 2023). These results suggest the initiation of two separate pathways upon SI pollen reception: the proteasomal ARC1 pathway for the degradation of compatibility factors, and a ROS response mediated by FERONIA, working in parallel to ARC1. Interestingly, although *MLPK* AS-ODNs reduced ROS, we did not observe a corresponding complete breakdown of SI, suggesting a more intricate, layered regulatory network underlying self-incompatibility in Brassicaceae (Bhalla et al., 2025a). RT-PCR analysis confirmed downregulation of all four target genes after AS-ODN treatment; however, *MLPK*’s inability to break SI despite transcriptional suppression further supports the interpretation that it has a secondary or redundant role in the SI pathway.

Overall, our results establish a robust platform for harnessing SI to improve major economic traits, enable hybrid seed production, and accelerate crop improvement in *Brassica rapa*. The study reveals a well-developed, naturally occurring system in this species that can be exploited to enhance oil and seed yield, develop hybrids, build resistance to both biotic and abiotic stresses, and adapt varieties to non-traditional environments. The characterized system could be deployed in self-compatible *Brassica rapa* lines, as previously validated in *Arabidopsis thaliana* (Nasrallah et al., 2002; Zhang et al., 2019; Fujii et al., 2020). To our knowledge, no prior work has comprehensively characterized this system in *Brassica rapa*, despite its traditional use in agriculture. Although this study has characterized the SI counterpart of this two-variety system, future studies elucidating bidirectional functional validation using complete overexpression and suppression systems remain an open avenue. Furthermore, functional validation through tissue-culture-based transgenics would further enhance the understanding of these candidates and their functional relevance. A deeper understanding of the molecular players and their manipulation through breeding or emerging biotechnology approaches will provide valuable tools for breeders and farmers seeking to maximize the genetic potential of this important oilseed crop.

## Data Availability

The datasets presented in this study can be found in online repositories. The names of the repository/repositories and accession number(s) can be found in the article/**Supplementary Material**.
