# Supplementary material for: Characterization of self-incompatibility genes in Brassica rapa var. toria and yellow sarson
Source: Front Plant Sci. 2026 Jul 2;17:1857745. doi: 10.3389/fpls.2026.1857745 (PMC13372785; doi:10.3389/fpls.2026.1857745)
Supplement: Supplementary file 1 [file DataSheet1.docx]

**Supplemental Material - 1**

**Gene sequences of molecular players regulating SI:**

***SRK***

ATGAAAGGTGTACGAAACATCTATCACCATTCTTACACCTCCTTTTTGCTCGTCTTCGTTATCATGATTCTATTTCATCCTACTCTTTCGATCTATATTAACGCTTTGTCGTCTACAGAATCTCTTACCATCTCAGGCAACAGAACACTTGTATCTCCCGGTGATGTCTTCGAGCTCGGTTTCTTCAAGACCACTTCAAGTTCTCGTTGGTATCTCGGTATATGGTACAAGAAACTCTCCGAAAGAACCTACGTATGGGTTGCCAACAGAGATAGCCCTCTCTCAAATGCCGTTGGAACCCTTAAAATCTCTAACATGAACCTGGTCCTCCTTGATCACTCTAATAAATCTGTTTGGTCGACGAATGCAACTAAAGGAAATGATAGATCTCCGGTGGTGGCAGAGCTTCTCGCTAACGGAAACTTCGTGATACGATACTCCAATAACAACGACGCAAGTAAATTTTTGTGGCAAAGTTTCGATTACCCTACAGATACTTTGCTTCCAGAGATGAAACTAGGTTACGACCTCAAAAAAGGGCTGAACAGATACCTTACATCATGGAGAAATTCAGATGATCCGTCAAGCGGGGAAATCTCATACCAAATAGACAATCAAACGGGAATCCCTGAGTTCTATCTATTGCAAACCGGCATACGAGTGCATCGGAGCGGTCCATGGAATGGAGTCCGATTTAGTGGCATACCAGGGGACCAAGAGTTAAGTTACATGGTGTACAATTTCACAGAGAATAGTGAAGAAGTCGCTTATACATTCCGAGTGACCGACAACAGCATATACTCGATATTGAAAACAAGTTCCGAAGGGTTTTTGGAGCGACAGACGTGGACCCTGAACTCAATTACATGGACCTTGTTCTGGTATTTACCATTGGAAAACCAGTGCGATATGTACATGATTTGTGGGCGTTATGCTTACTGTGATGTGAACACATCACCGTTGTGTAACTGTATCCATGGGTTCATACCCTGGAATAAGCAGCAGTGGGAGATGATAAATCCGGCAGGTGGGTGTATAAGGAGGACGCGGCTGAGCTGCAGTGGTGATGGTTTTACCAGGATGAAGAAGATGAAGTTGCCAGAGACGAAGATGGCGATTGTCGACAGGAGTATTGGTGTGAAAGAATGTGAGAAAAGGTGCCTTAGCAATTGTAATTGTACAGCTTTTGCAAATGCGGATATCCGGAATGGCGGGACGGGTTGTGTGATTTGGACAGGAGACCTCGAGGATCTTCGAAATTACTATGCTGACGGTCAAGATCTTTATGTCAGATTGGCTGCCGCTGATCTTGTTAAAAAGAAAAACGCGAATTGGAAAATCATAAGTTTGATTGTTGGAGTTAGTGTTGTTCTGCTTCTGCTTCTTCTGATCATGTTCTGCCTTTGGAAAAGGAAACAAAATCGAGCAAAAGCAATGGCAACATCTATTGTCAATCAACAGAGAAACCAAAATGTACTTATGAACACGATGACACAATCAGACAAGAGACAGTTGTCTAGAGAGAACAAAGCTGATGAAGTCCAACTTCCATTGATACAGGTGGAAGCTGTTGCCAAAGCCACCGCAAATTACTCCCATTGTAACGAACTTGGACGAGGTGGTTTCGGTATTGTTTACAAGGGGATGCTTGATGGGCCAAATGTTGCGGTAAAAAGGCTATCAAAGACATCCCTTCAAGGCATTGATGAGTTTATGAATGAGGTGAGATTGATCGCAAGGCTTCAGCATATAAACCTTGTCCGAAGTCTTGGCTGTTGCAATGAAGCGGACGAGAAGATTCTGATATATGAGTATTTGGAAAAATCAAGCCTGGATTATTTTCTCTTTGGAACAAAACAAAGCTCTAACTTAAGTTGGAAGGACAGATTCGCCATTACCAATGGTGTTGCTCGAGGGCTTTTATATCTACATCATGACTCACGGTTTAGGATAATCCACCGGGATTTGAAACCAGGTAACATTACGCTTGATAAATATAGGATCCCACGGATCTCGGATTTTGGGATGGCCAGAAACATAGCCAGGGATGAAACTCAAGTTAGGACAGACAATGCGGTCGGAACCAACGGCTACTGGTCTCCGGAAAACGCAATGTATGGGGTAATCTCGGGAAAAACAGATGTTTTCAGTTTTGGAGTCATAGTTCTTGAAATTGTTATTGGAAAAAGAAATAGAGGATTCTACCAGGTGAACCCTGAAAACAATCTTCCAAGCTATGCATGGACTCATTGGGCGGAGGGAAGAGCGCTAGAAATCGTAGATCCAGTCATCTTAGATTCATTGTCATCTCTGCCATCAACATTTAAACCAAAAGAAGTCCTAAAATGCATACAAATTGGTCTATTGTGTATTCAAGAACGTGCGGAGCACAGACCAACGATGTCGTCGGTGGTTTGGATGCTTGGAAGTGAAGCAACAGAGATTCCTCAGCCTAAACCGCCAGTTTATTGTCTCATAGCAAGTTATTATGCAAATAATCCTTCCTCAAGTAAGCAATTCGATGACGATGAATCCTGGACAGTGAACAAGTACACCTGCTCAGTCATCGATGCCCGGTAA

***FER1***

ATGAAGATAACTGAGGGACAATCACGTCTCTCCCTCCTCCTCCTCCTCCTTCTCTTATCCTTATCTTCATCAACCTCAGCTGCTGACTACACTCCCACTGACAAGATCCTCTTAAACTGTGGTGGCTCCTCTGATCGTACCGACACAGATAACCGGACATGGATCCCCGATGTCAAATCCAAGTTCTTGTCTTCCTCCGGAGACTCCAAAACCTCTCCCGCCGCCACACAAGACCCTTCCGTCCCCGAGGTTCCTTACATGACAGCTAGAATCTTCCGGTCTCCCTTCACTTACTCTTTCCCCGTCGCTTCAGGTCGTAAGTTCGTGCGTCTCTACTTCCACCCCAACTCATACGACGGCCTCAACGCCACGACCTCCCTCTTCTCCGTCACCTTAGGCTCCTCCTACACTCTCCTCAAGAACTTCAGCGCTGCTCAAACAGCTCAGGCCTTGTCTTACTCCTCCATCGTTAAAGAGTTTATCGTGAACGTGGAAGGTGGAGCTTCTTTGAACATAACGTTCACACCTGAATCAACACCAAAGGCTTATGCCTTTGTGAACGGTATTGAGGTGACTTCAATGCCTGATCTATACAGCAACACTGATGGGACTTTGTCCATCGTGGGATCTTCTACTGCGGTCGATATCGATAACAGCACTGCTCTTGAGAATGTTTACAGGCTTAACGTTGGAGGGAATGATATCTCTCCTTCTGAAGATACAGGTCTTTACAGGTCATGGTACGACGACTCGCCTTACATTTTCACTGCGGGGATTGGAGTCGTTGAGACTGTTGATCCCAACATGACCATTAAGTATCCCACGGACACACCTACATACATTGCTCCTGTTGATGTTTACTCAACTGCTAGGTCTATGACTCCCACAGCTCAAATCAACCTCAACTTCAACCTGACTTGGGTTTTCAGCATTGACTCTGGCTTCACTTATCTTGTTAGGCTTCATTTCTGCGAGGTTCTTCCCGACATCACTAAGATTAACCAGCGTGTGTTTACAATCTACCTCAACAACCAAACAGCTGAGTCTGAAGCTGATGTTGCTGGCTGGACGGGTGGTAATGGGATTCCTATATATAAAGACTACGTTGTGAATCCTCCTGATGGTAAGGGACAGCAAGATCTTTGGCTTGCTCTTCATCCAAACACGAGGGGCAAGCCGGAGTACTACGATGCTATTCTTAATGGAGTTGAGATTTTCAAGATGAATGGTTCTGATGGTAATCTTGCTGGTCCTAATCCTATACCTGGTCCGCAAGTGACTGCAGATCCATCCAGAGTGTTACGCCCTCGCACTGGTTCATCTAAGAGCCATACAGCTATTGTTGCAGGTGTAATCAGTGGTGCAGTTGTTTTAGGTCTTATTGTTGGTTTATGTGTAATGGTTGCTTACCGTAGACGTAAGGCTGGTGAATACCAGCCTGCAAGTGATGCAACATCAGGGTGGCTTCCACTGTCTTTGTATGGAAACTCACATTCTGGTGGCTCGGGTAAGACAAACACTACAGGAAGCTACGCCTCGTCCCTTCCTTCAAACCTGTGTCGTCACTTCTCCTTTGCTGAGATCAAAGCAGCTACTAAGAACTTTGATGAGTCTCGAGTGCTCGGTGTTGGAGGTTTTGGTAAGGTGTACAGAGGAGAGATTGATGGTGGAACTACAAAGGTAGCCATCAAGAGAGGCAACCCTATGTCTGAGCAAGGTGTGCACGAGTTTCAGACAGAGATTGAGATGCTTTCGAAGCTTAGACACCGTCACCTTGTGTCTTTGATTGGATACTGTGAAGAGAACTGCGAGATGATACTTGTGTATGATTACATGGCTCATGGGACAATGAGAGAGCATCTCTACAAGACTCAGAACTCTCCTCTTCCTTGGAAGCAACGTCTTGAGATATGCATTGGGGCAGCAAGAGGGTTGCATTATCTACACACCGGTGCGAAACACACGATCATCCACAGAGATGTGAAGACGACAAACATTCTGTTGGATGAGAAGTGGGTGGCTAAGGTCTCTGACTTCGGTCTGTCAAAGACTGGTCCTACACTTGACCACACACATGTTAGCACGGTGGTGAAAGGAAGCTTCGGTTATCTCGACCCAGAGTACTTCAGACGTCAGCAACTGACTGATAAATCAGATGTCTACTCCTTTGGTGTCGTTCTTTTCGAAGCTCTATGCGCACGGCCTGCCTTGAACCCGACGCTAGCAAAAGAACAAGTGAGCTTAGCTGAGTGGGCACCATACTGCTACAAGAAAGGCATGCTTGACCAGATCGTTGATCCGCATCTCAAGGGCAAGATCACACCGGAATGCTTCAAGAAGTTTGCTGAAACCGCGATGAAGTGTGTACTAGACCAGGGCATTGAGAGACCGTCGATGGGAGATGTTCTCTGGAACTTAGAGTTCGCGTTGCAGCTTCAGGAAAGCGCTGAGGAGAGCGGGAAAGGGATATGCAGTGAGATGGACATGGGTGAGATTAAGTACGATGATGATAACTGTAAAGGGAAGAGCAACAACGACAAGGGCTCTGATGTGTATGAAGGGAATGTTAGTGACTCGAGGAGCAGTGGTATAGACATGAGTATTGGTGGTAGGAGTTTGGTCAGTGAAGATTCAGATGGACTCACTCCAAGTGCTGTGTTTTCTCAGATCATGAATCCTAAGGGACGTTAG

***MLPK***

ATGGGGATTTGCTTGAGTGCTCAGATTAAAGCTGAGAGTCCAAGTAACACAGGTGCGAGTCCGAAGTATATGAGCTCAGAGGCAAATGATACACAGAGCATGGGAAGCAAAGGCTCTTCTGTGTCGATCAGAACAAACCCTCGAACCGAAGGAGAGATCTTGCAATCTCCAAACCTCAAAAGTTTTAGCTTCGCTGAGGTCAAATCAGCAACTAGGAATTTCAGACCAGACAGTGTTCTTGGTGAAGGTGGATTCGGTTGTGTGTTTAAAGGATGGATTGATGAGCAATCTCTCACTGCGTCTAAACCGGGAACCGGTATGGTTATTGCTGTCAAAAGACTTAACCAAGATGGTTGGCAAGGTCATCAAGAATGGCTGGCGGAAGTGGATTACTTGGGGAAGTTCTCTCATCCTAATCTTGTGAAACTCATCGGTTATTGTTTAGAGGATGAGCAACGTCTTCTTGTGTATGAGTTCATGCCACGTGGAAGCTTAGAGAATCATTTATTCAGAAGAGGTTCTTACTTTGAACCATTATCTTGGACTCTCAGATTGAAAGTTGCACTTGGCGCTGCAAAAGGCCTAGCTTTTCTTCACAACGCGGAGACTCAAGTCATATACCGGGACTTCAAAACTTCTAACATACTTATTGATTCGGACTACAACCCCAAGCTTTCTGATTTTGGGTTGGCTAAAGACGGTCCAACAGGTGATAAAAGCCATGTCTCCACAAGAATCATGGGTACTTATGGATACGCAGCTCCTGAGTATCTTATGACAGGTCATTTAACAACCAAGAGTGATGTCTATAGCTACGGTGTTGTGCTTTTGGAGATACTCTCTGGACGTAGAGTTGTAGACAAGAACCGTCCACCGGGAGAGCAAAAACTGGTGGATTGGGCAAAACCGTTGCTTGCAAACAAGAGGAAGATCTTTAGAGTTATCGATAACCGTCTACAAGATCAGTACTCAATGGAAGAAGCGTGTAAAGTAGCTACTCTAGCGCTGAGATGCCTGACGACAGAGATAAAGCTGAGACCAAACATGACTGAGGTTGTTGCTCACCTCGAACACATACAAACTTTGCATGAAACAGGAGGAGGAAGAAACATTGATAAGTTGGAGAGGAGAACGCGTAGGAGAAGTGATAGTGTTGTGGTGAGCCAAAAACCAAATGCTGGTTTTGCTAGACAAAGTGCTGTGGGTGGAATAGCAGCTGCGTATCCACGTCCCTCTGCTTCGCCTCTGTTTGTCTGA

***ARC1***

ATGGCCACTGATTCAGCAATGTTCGCATCCTCACGTCGGAGGCAATCTCCGTCGCTTGAGGCGTTTCTATCACCCGTTGATCTCTCCGACGTCCCTCTCCTCCAAACACTATCTTCCATCTCATCAGAGATCGTCTCCTGCTTCAGCAACGCACGTTTCTCCTTCCAACGTAGAAACACCCGTTCCCTGATACGTAAAGTCCAAGTCTTCGCCGTCTTACTCCAACACCTCGCACCCGAGTCAAGCTTGGATCCCACGGCGGTGCTCTGCTTCAAGGAGCTCTATCTCCTCCTCCACCACTCCAAGTTCCTCCTCCGCTACTGCGCTCACTCCTCCAAGCTATGGCTCTTGCTTCAAAGCCCCTCGCTCTCGAGCTTCTTCCATGATCTGAGTAAAGACTATTCCACCCTCTTAGATGTCCTCCTCCCTGCTGAGAGTCTCTGCCTAAACGACGACGTTAGAGAGCAAGTCCAGCTCTTGCACATGCAGCACTACATTGACGATAACAGCGACGAGACGCTGCGTAACAAACTCTATTCGTTTCTAGACGAGTTCGAGAACGGGAGTGTACCAAACTCTGAAGAGCTACGCTTCTTCTTCTTTGAGAAACTCGCTATTAAAGATCCAACAAGTTACAGAGAAGAGATCGAGTTTCTTGAAGAGCAGATCAAAAGCCACGGGTGTGACTTAGACCCTACGAGGTCAGTGATCAACGGGTTTATAGATATCACACGGTACGTTATGTTTCTCTTATTCAAGATTGAAGATGGTAACGAGATTAACAAACAGAAGAAACGTTTGATCTCTGAGGAGATTGAGAACACGTTTACAACAACGCTTCCAAAGGATTTCATCTGCTCCATCTCTCTCAACCTCATGAACGATCCTGTGATCATCTCCACGGGACAGACTTACGATAGAACCTCCATCGCTAGGTGGATTCATCAAGAAGGTCGCTCTACTTGTCCCAAAACAGGACAGAAGCTCGTGGACTTGAGTTTCGTTCCCAACCTAGCTTTGAGACACTTGACAAAGCTTTGGTGCCAAGTCACTGGTCTGTCTCATGACTCGCCTAAAGAGTCTCTCCCAAAGGTGTTTCAAACAAGAGCTTCCACGGAAGCAAACAAAGCAACGTTATCGATTCTTGTACAGAACCTAGCACACGGCTCAGAGTTGGCTGCAGGAGAGATCCGTGTTCTCACTAGAACAGTAACGGAAACGCGTACGTTGATCGTGGAAGCAGGTGCGATCCCGTATCTGCGTAGTCTTCTCAAATCCCAAAACGCTGTTGCGCAGGAGAACGCAGTTGCATCGATCTTTAACTTATCTATAGACGAAGAAAACAGGAGTCTGATCATGGAGGAACACTCTTGTCTCGAGCCGATGATGAGCGTTCTCGTCTCTGGTCTTACGATGAGAGCTAAGGAGATAGCAGCAGCCACGTTGCACACTCTTTCCAGCGTACATGATTACAAGAAAACGATCGCTAACGCTGATGGATGCATCGAGGCGCTTGCACTGGTGCTGCGAAACGGAACCGTGAGAGGGAAGAAAGATGCTGTCTACGCTTTGCATAGCTTATGGCTGCATCCGGATAACTACAGCTTGATGGTAAAAAGGGGAGGAGTGTCTGCTCTCGTTGGAGCTTTAGGGGAAGAGGCTGTGGCGGAGAAAGTTGCGTGGGTGTTGGGTGTGATGGCTACTGAGACTTTAGGAGCTGAGAGTATAGGGAGAGAGGAATCAGTTGTGACGGGGCTCATGGAACTAATGAGATGTGGAAGACCTAGAGGCAAAGAAAAAGCTATTGCGACTTTGTTACAACTCTGCACAGCAGGTGGAGCGGTTGTGACGGAGAAGGTTGTGAAAACACCCGCTCTTGCGGTCTTGACGCGTAAGCTTTTGCTCACGGGTACAGACCGAGCTAAGAGGAAAGCGGTTTCACTCTCTAAGGTATGTAAGGGGTGCGACCAGAAAACACAGAGATAA

**Supplementary Figure legends**

**Supplementary Figure S1.** Morphological overview of *Brassica rapa* var. toria (TOR) and yellow sarson (YS).

(A) Mature plants from the self-incompatible variety toria (Left) and the compatible variety yellow sarson (Right).

(B) Close-up of flowers from each variety, highlighting key floral parts and transverse section. Note: Scale bars 5 mm

(C) Sequential floral developmental stages from complete flower and transverse section: stage 1: unopened bud, stage 2: slightly opened flower, and stage 3: opened flower from each variety showing stamen and pistil. Note: Scale bar 1 mm

**Supplementary Figure S2.** Germination assay of F1 seeds obtained from various crosses between *B.rapa* var. toria and yellow sarson.

**Supplementary Figure S3.** PCR optimization, Colony PCR, and Restriction Digestion-based analysis of various genes regulating self-incompatibility in *B.rapa* var. toria.

(A) Gel images showing PCR products of various genes under study. Note: The size of the PCR products is indicated.

(B) Gel images showing colony PCR of various DB3.1 colonies transformed by TOPO cloning-based products of various genes under study. Note: The size of the PCR band is indicated, and red asterisks indicate positive colonies.

(C) Gel images showing Restriction digestion-based products of various TOPO-gene constructs using EcoR1. Note: The size of the Restriction digestion products is indicated.

**Supplementary Figure S4.** Full scan of gel images for the *SRK*, *FER1*, *MLPK*, and *ARC1* in unpollinated (UP), self-incompatible (SI30 and SI60 – 30 and 60 MAP), and compatible (CP30 and CP60 – 30 and 60 MAP) pollinated stigmas. Note: *BrActin 7* (NCBI accession: KU851921) was used as an internal control. Band sizes are provided in the figure.

**Supplementary Figure S5.** Graphical representation of proteins (A) SRK, (B**)** FER1, (C) MLPK, and (D) ARC1 and their conserved domains.

**Supplementary Figure S6.** Secondary structure prediction of (A) SRK, (B) FER1, (C) MLPK, and (D) ARC1 proteins from *Brassica rapa* var. toria predicted through PSIPRED.

**Supplementary Figure S7.** Ramachandran plots of AlphaFold3 predicted 3-D structures (A) SRK, (B) FER1, (C) MLPK, and (D) ARC1, indicating the accuracy and reliability of the predicted structures.

**Supplementary Figure S8.** Illustration of the top-dripping method for *in vitro* ODN treatment.

**Supplementary Figure S9.** Functional characterization of genes during compatibility response.

(A-D) Representative aniline blue images of pollinated stigmas treated with Mock, Sense, and Anti-Sense ODNs of (A) *SRK*, (B) *FER1*, (C) *MLPK*, and (D) *ARC1*, showing pollen attachment and tube formation. Note: Aniline blue (first), Red channel (second), Bright field (third), Zoomed (fourth). Note: Scale bars: 300 μm & 50 μm (zoomed), white arrows indicate pollen grains with emerging pollen tubes

(E-H) Graph representing the number of pollen tubes per stigma observed in (Figure 5A-D) & (Supplementary Figure S10A-D) treated with Mock, Sense, and Anti-Sense ODNs of (E) *SRK*, (F) *FER1*, (G) *MLPK*, and (H) *ARC1*. Note: Dots represent individual data points. One way ANOVA, *P < 0.05, n = 9, ns= not significant

**Supplementary Figure S10.** Optimization of Nitro Blue Tetrazolium (NBT) assay.

(A) Representative images of stigma stained with NBT at unpollinated or 15, 30, and 60 MAP during SI response. Note: Scale bars: 300 μm

(B) Graph representing the reverse normalized intensity of ROS for various time points after the self-incompatibility response. Note: Higher intensity indicates more NBT staining and ROS levels One-way ANOVA, *p<0.05, n=15

**Supplementary Figure S11.** Gel image for the *SRK*, *FER1*, *MLPK*, and *ARC1* treated with ODNs - Mock, Sense, and Anti-Sense. Note: *BrActin 7* (NCBI accession: KU851921) was used as an internal control. Control images are reused for illustrative purposes.

**Supplementary Figure S12.** Full scan of gel images for the *SRK*, *FER1*, *MLPK*, and *ARC1* treated with ODNs - Mock, Sense, and Anti-Sense. Note: *BrActin 7* (NCBI accession: KU851921) was used as an internal control, M- Mock, S- Sense, AS- Anti-Sense. Band sizes are provided in the figure.

**Supplementary Tables**

**Supplementary Table S1.** List of primers used for cloning in this study.

| **S.No.** | **Gene** | **Primers** | **Template Size** | **NCBI Accession** |
| --- | --- | --- | --- | --- |
| 1 | *SRK* | FP: ATGAAAGGTGTACGAAACATC | 2580 bp | OR887605 |
|  |  | RP: TTACCGGGCATCGATGACTGA |  |  |
| 2 | *FER1* | FP: ATGAAGATAACTGAGG | 2616 bp | PX355005 |
|  |  | RP: CTAACGTCCCTTAGGA |  |  |
| 3 | *MLPK* | FP: ATGGGGATTTGCTTGAGTGC | 1257 bp | PV420907 |
|  |  | RP: TCAGACAAACAGAGGCGAAG |  |  |
| 4 | *ARC1* | FP: ATGGCCACTGATTCAGCAATG | 1983 bp | PX058862 |
|  |  | RP: TTATCTCTGTGTTTTCTGGTC |  |  |

**Supplementary Table S2.** PCR conditions for amplification of genes used in this study.

| **Gene** | **Initial denaturation** | **Denaturation** | **Annealing** | **Extension** | **Final extension** |
| --- | --- | --- | --- | --- | --- |
| No. of cycles | 1x | 35 Cycles | | | 1x |
| *SRK* | 98ºC; 2 min | 98ºC; 30 sec | 61ºC; 30 sec | 72 ºC; 1 min and 15 secs | 72 ºC; 10 min |
| *FER1* |  |  | 49ºC; 30 sec | 72 ºC; 1 min and 30 secs |  |
| *MLPK* |  |  | 63ºC; 30 sec | 72 ºC; 45 secs |  |
| *ARC1* |  |  | 59ºC; 30 sec | 72 ºC;1 min |  |

**Supplementary Table S3.** List of the *SRK* gene sequences retrieved from the NCBI database for phylogenetic analysis.

| **S.No.** | **Accession number** | **Gene Description** |
| --- | --- | --- |
| 1 | OR887605 | *Brassica rapa* cultivar toria S receptor kinase |
| 2 | AB219163.1 | *Brassica rapa* BrSRK-54f S receptor kinase |
| 3 | AB032474.1 | *Brassica oleracea* SRK60 S60 S-locus receptor kinase |
| 4 | AB298890.1 | *Brassica oleracea* SRK-4 mRNA S-locus receptor kinase |
| 5 | AB054061.1 | *Brassica rapa* SRK22 mRNA S locus receptor kinase |
| 6 | AB270777.1 | *Brassica napus* BnSRK-3 pseudogene S receptor kinase |
| 7 | D38564.2 | *Brassica campestris* SRK12 receptor protein kinase |
| 8 | D38563.1 | *Brassica campestris* SRK8 receptor protein kinase |
| 9 | EU180597.1 | *Brassica oleracea* var. acephala SRK13-b receptor kinase |
| 10 | AB298885.1 | *Brassica rapa* SRK-55 S-locus receptor kinase |
| 11 | AB298887.1 | *Brassica rapa* SRK-61 S-locus receptor kinase |
| 12 | AB270775.1 | *Brassica rapa* BrSRK-21 S receptor kinase |
| 13 | M97667.1 | *Brassica napus* ssp. oleifera serine/threonine kinase receptor |
| 14 | AB032473.1 | *Brassica oleracea* SRK18 S-locus receptor kinase |
| 15 | M76647.1 | *Brassica oleracea* SKR6 receptor protein kinase |
| 16 | AB270776.1 | *Brassica napus* BnSRK-2 pseudogene S receptor kinase |
| 17 | AB298875.1 | *Brassica rapa* SRK-25 S-locus receptor kinase |
| 18 | AB270767.1 | *Brassica napus* BnSRK-1 S receptor kinase |
| 19 | AB013720.1 | *Brassica oleracea* SRK23Bol S receptor kinase |
| 20 | AB298891.1 | *Brassica oleracea* SRK-14 S-locus receptor kinase |
| 21 | AB298884.1 | *Brassica rapa* SRK-53 S-locus receptor kinase |
| 22 | LC556298.1 | *Brassica rapa* BrSRK-9 S-locus receptor kinase |
| 23 | AB298902.1 | *Brassica oleracea* SRK-61 S-locus receptor kinase |
| 24 | U00443.1 | *Brassica napus* cultivar T2 S-receptor kinase |
| 25 | AB298901.1 | *Brassica oleracea* SRK-52 S-locus receptor kinase |
| 26 | AB298886.1 | *Brassica rapa* SRK-56 S-locus receptor kinase |
| 27 | AB012106.1 | *Brassica rapa* SRK45 S-locus receptor kinase |
| 28 | AB298905.1 | *Brassica oleracea* SRK-68 S-locus receptor kinase |
| 29 | AB052756.1 | *Arabidopsis lyrata* SRKb S-locus receptor kinase |

**Supplementary Table S4.** List of the *FER1* gene sequences retrieved from the NCBI database for phylogenetic analysis.

| **S.No.** | **Accession number** | **Gene Description** |
| --- | --- | --- |
| 1 | PX355005.1 | *Brassica rapa* cultivar toria FER1 |
| 2 | EF681131.1 | *Brassica oleracea* FER1 |
| 3 | XM_048743008.1 | *Brassica napus* FER1 |
| 4 | EF681137.1 | *Arabidopsis thaliana* FER1 |

**Supplementary Table S5.** List of the *MLPK* gene sequences retrieved from the NCBI database for phylogenetic analysis.

| **S.No.** | **Accession number** | **Gene Description** |
| --- | --- | --- |
| 1 | PV420907.1 | *Brassica rapa* M locus protein kinase |
| 2 | XM_013888729.3 | *Brassica napus* probable serine/threonine-protein kinase (predicted) |
| 3 | KC576522.1 | *Brassica rapa* subsp. chinensis M locus protein kinase |
| 4 | XM_013780614.1 | *Brassica oleracea* var. oleracea protein kinase chloroplastic (predicted) |
| 5 | NM_001036363.2 | *Arabidopsis thaliana* protein kinase 1B PK1B |

**Supplementary Table S6.** List of the *ARC1* gene sequences retrieved from the NCBI database for phylogenetic analysis.

| **S.No.** | **Accession number** | **Gene Description** |
| --- | --- | --- |
| 1 | PX058862.1 | *Brassica rapa* cultivar toria armadillo repeat containing 1 |
| 2 | PX058861.1 | *Brassica rapa* cultivar yellow sarson armadillo repeat containing 1 |
| 3 | KC576518.1 | *Brassica rapa* subsp. chinensis armadillo repeat containing 1 |
| 4 | AF024625.1 | *Brassica napus* arm repeat containing 1 |
| 5 | EU344909.1 | *Brassica oleracea* var. acephala arm repeat containing protein 1 |

**Supplementary Table S7.** List of conserved domains and their location in various proteins under study.

| **Protein** | **Name of domains** | **Accession** | **Description** | **Internal** | **E-value** |
| --- | --- | --- | --- | --- | --- |
| SRK | STKc_IRAK | [cd14066](https://www.ncbi.nlm.nih.gov/Structure/cdd/cddsrv.cgi?ascbin=8&maxaln=10&seltype=2&uid=cd14066) | Catalytic domain of the Serine/Threonine kinases, | 537-808 | 6.54 × 10^-79^ |
|  | B_lectin | [pfam01453](https://www.ncbi.nlm.nih.gov/Structure/cdd/cddsrv.cgi?ascbin=8&maxaln=10&seltype=2&uid=pfam01453) | D-mannose binding lectin | 83-189 | 2.94 × 10^-47^ |
|  | S_locus_glycop | [pfam00954](https://www.ncbi.nlm.nih.gov/Structure/cdd/cddsrv.cgi?ascbin=8&maxaln=10&seltype=2&uid=pfam00954) | S-locus glycoprotein domain | 223-330 | 1.39 × 10^-40^ |
|  | PAN_2 | [pfam08276](https://www.ncbi.nlm.nih.gov/Structure/cdd/cddsrv.cgi?ascbin=8&maxaln=10&seltype=2&uid=pfam08276) | PAN-like domain | 354-418 | 3.20 × 10^-29^ |
|  | DUF3403 | [pfam11883](https://www.ncbi.nlm.nih.gov/Structure/cdd/cddsrv.cgi?ascbin=8&maxaln=10&seltype=2&uid=pfam11883) | Domain of unknown function (DUF3403) | 810-859 | 5.75 × 10^-10^ |
|  | DUF3660 | [pfam12398](https://www.ncbi.nlm.nih.gov/Structure/cdd/cddsrv.cgi?ascbin=8&maxaln=10&seltype=2&uid=pfam12398) | Receptor serine/threonine kinase | 483-520 | 1.98 × 10^-7^ |
| FER1 | STKc_IRAK | [cd14066](https://www.ncbi.nlm.nih.gov/Structure/cdd/cddsrv.cgi?ascbin=8&maxaln=10&seltype=2&uid=cd14066) | Catalytic domain of the Serine/Threonine kinases, Interleukin-1 Receptor Associated Kinases and related STKs | 541-807 | 1.21 × 10^-88^ |
|  | PK_Tyr_Ser-Thr | [pfam07714](https://www.ncbi.nlm.nih.gov/Structure/cdd/cddsrv.cgi?ascbin=8&maxaln=10&seltype=2&uid=pfam07714) | Protein tyrosine and serine/threonine kinase | 539-735 | 2.09 × 10^-49^ |
|  | STYKc | [smart00221](https://www.ncbi.nlm.nih.gov/Structure/cdd/cddsrv.cgi?ascbin=8&maxaln=10&seltype=2&uid=smart00221) | Protein kinase; unclassified specificity | 539-735 | 5.32 × 10^-49^ |
|  | SPS1 | [COG0515](https://www.ncbi.nlm.nih.gov/Structure/cdd/cddsrv.cgi?ascbin=8&maxaln=10&seltype=2&uid=COG0515) | Serine/threonine protein kinase Signal transduction mechanisms | 539-735 | 6.49 × 10^-46^ |
|  | Malectin_like | [pfam12819](https://www.ncbi.nlm.nih.gov/Structure/cdd/cddsrv.cgi?ascbin=8&maxaln=10&seltype=2&uid=pfam12819) | Malectin-like domain | 38-408 | 1.81 × 10^-40^ |
| MLPK | STKc_IRAK | [cd14066](https://www.ncbi.nlm.nih.gov/Structure/cdd/cddsrv.cgi?ascbin=8&maxaln=10&seltype=2&uid=cd14066) | Catalytic domain of the Serine/Threonine kinases, Interleukin-1 Receptor Associated Kinases and related STKs | 80-359 | 1.08 × 10^-96^ |
|  | TyrKc | [smart00219](https://www.ncbi.nlm.nih.gov/Structure/cdd/cddsrv.cgi?ascbin=8&maxaln=10&seltype=2&uid=smart00219) | Tyrosine kinase, catalytic domain | 77-356 | 7.89 × 10^-54^ |
|  | PK_Tyr_Ser-Thr | [pfam07714](https://www.ncbi.nlm.nih.gov/Structure/cdd/cddsrv.cgi?ascbin=8&maxaln=10&seltype=2&uid=pfam07714) | Protein tyrosine and serine/threonine kinase | 79-356 | 2.57 × 10^-53^ |
|  | SPS1 | [COG0515](https://www.ncbi.nlm.nih.gov/Structure/cdd/cddsrv.cgi?ascbin=8&maxaln=10&seltype=2&uid=COG0515) | Serine/threonine protein kinase [Signal transduction mechanisms] | 73-416 | 2.51 × 10^-49^ |
|  | PLN00113 | [PLN00113](https://www.ncbi.nlm.nih.gov/Structure/cdd/cddsrv.cgi?ascbin=8&maxaln=10&seltype=2&uid=PLN00113) | leucine-rich repeat receptor-like protein kinase | 50-357 | 4.93 × 10^-24^ |
|  | knB_PASTA_kin | [NF033483](https://www.ncbi.nlm.nih.gov/Structure/cdd/cddsrv.cgi?ascbin=8&maxaln=10&seltype=2&uid=NF033483) | Stk1 family PASTA domain-containing Ser/Thr kinase | 178-283 | 2.59 × 10^-11^ |
| ARC1 | RING-Ubox_PUB | [cd16664](https://www.ncbi.nlm.nih.gov/Structure/cdd/cddsrv.cgi?ascbin=8&maxaln=10&seltype=2&uid=cd16664) | U-box domain, a modified RING finger, found in *Arabidopsis* plant U-box proteins (AtPUB) and similar proteins | 281-332 | 5.00 × 10^-24^ |
|  | U-box | [smart00504](https://www.ncbi.nlm.nih.gov/Structure/cdd/cddsrv.cgi?ascbin=8&maxaln=10&seltype=2&uid=smart00504) | Modified RING finger domain | 283-347 | 9.36 × 10^-24^ |
|  | U-box | [pfam04564](https://www.ncbi.nlm.nih.gov/Structure/cdd/cddsrv.cgi?ascbin=8&maxaln=10&seltype=2&uid=pfam04564) | U-box domain | 281-341 | 3.24 × 10^-14^ |
|  | PLN03200 | [PLN03200](https://www.ncbi.nlm.nih.gov/Structure/cdd/cddsrv.cgi?ascbin=8&maxaln=10&seltype=2&uid=PLN03200) | Cellulose synthase-interactive protein | 374-624 | 9.75 × 10^-8^ |
|  | Arm | [pfam00514](https://www.ncbi.nlm.nih.gov/Structure/cdd/cddsrv.cgi?ascbin=8&maxaln=10&seltype=2&uid=pfam00514) | Armadillo/beta-catenin-like repeat | 406-443 | 2.04 × 10^-4^ |

**Supplementary Table S8.** List of oligonucleotides used in this study.

| **S.No.** | **Gene** | **Sense/Antisense** | **Sequence** |
| --- | --- | --- | --- |
| 1 | *SRK* | Sense | T*A*G*CCCTCTCTCAAATG*C*C*G |
|  |  | Antisense | C*G*G*CATTTGAGAGAGGG*C*T*A |
| 2 | *FER1* | Sense | A*T*G*AAGATAACTGAGGG*A*C*A |
|  |  | Antisense | T*G*T*CCCTCAGTTATCTT*C*A*T |
| 3 | *MLPK* | Sense | A*T*G*GGAAGCAAAGGCTC*T*T*C |
|  |  | Antisense | G*A*A*GAGCCTTTGCTTCC*C*A*T |
| 4 | *ARC1* | Sense | G*A*C*GTCCCTCTCCTCCA*A*A*C |
|  |  | Antisense | G*T*T*TGGAGGAGAGGGAC*G*T*C |

Note- Asterisks (*) indicate phosphorothioate modifications at the three terminal bases on both the 5’ and 3’ ends.

**Supplementary Table S9.** List of primers used to perform RT-PCR in this study.

| **S.No.** | **Gene** | **Primers** | **Amplicon size (bp)** |
| --- | --- | --- | --- |
| 1 | *SRK* | FP: CAGACGTGGACCCTGAACTC | 209 |
|  |  | RP: CGCGTCCTCCTTATACACCC |  |
| 2 | *FER1* | FP: ACCTACATACATTGCTCCTGTTGA | 187 |
|  |  | RP: TGTAAACACACGCTGGTTAATCTT |  |
| 3 | *MLPK* | FP: GGTTGGCTAAAGACGGTCCA | 223 |
|  |  | RP: GGTTTTGCCCAATCCACCAG |  |
| 4 | *ARC1* | FP: AGTCAAGCTTGGATCCCACG | 219 |
|  |  | RP: CGTCGTCGTTTAGGCAGAGA |  |
| 5 | *BrActin 7* | FP: TGGTTCGACCATGTTCCCTG | 213 |
|  |  | RP: CTGTGGACGATGGATGGACC |  |

**Supplementary Figures**

**Supplementary Figure S1**

**
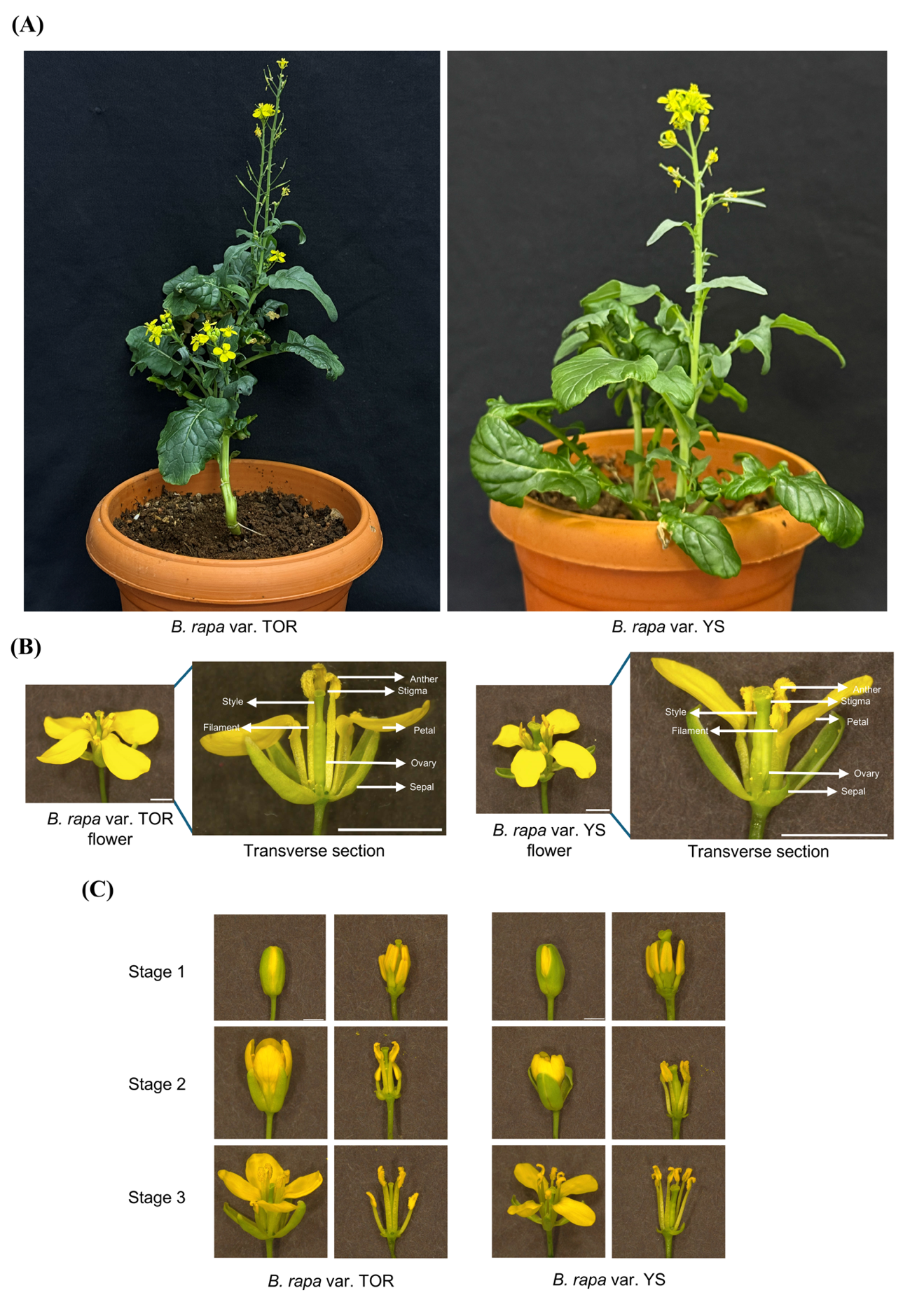
**

**Supplementary Figure S2**

**
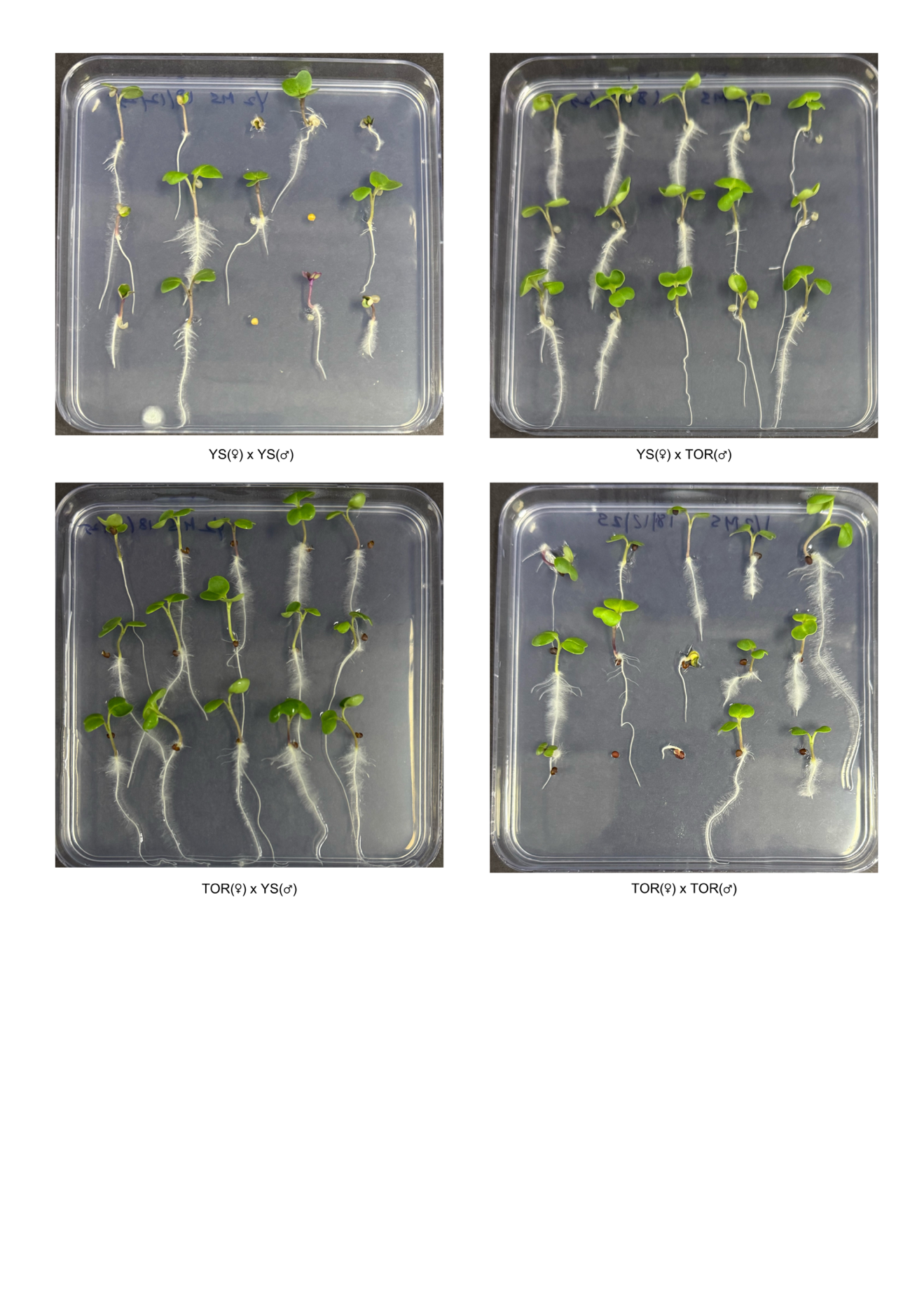
**

**Supplementary Figure S3**

**
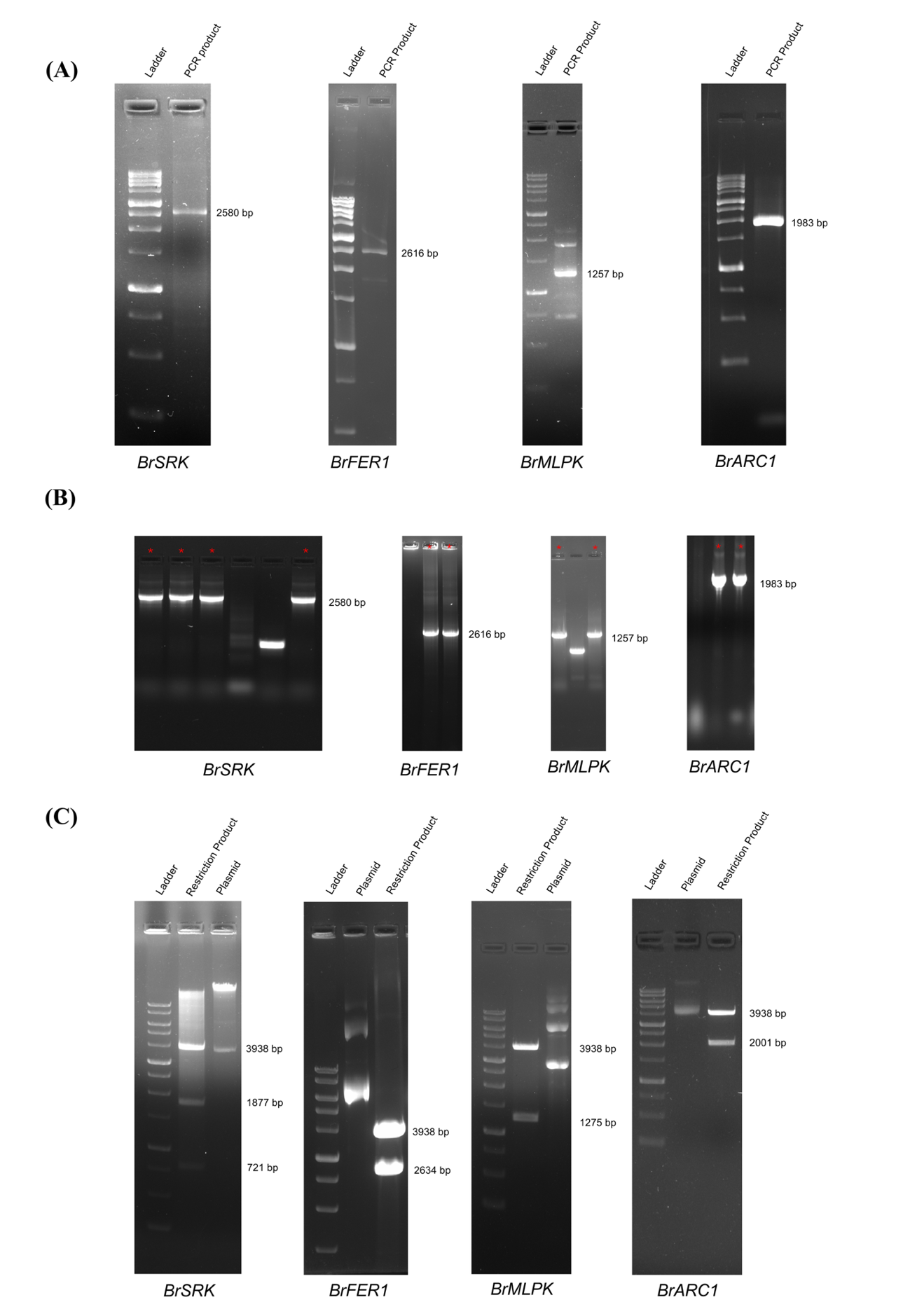
**

**Supplementary Figure S4**

**
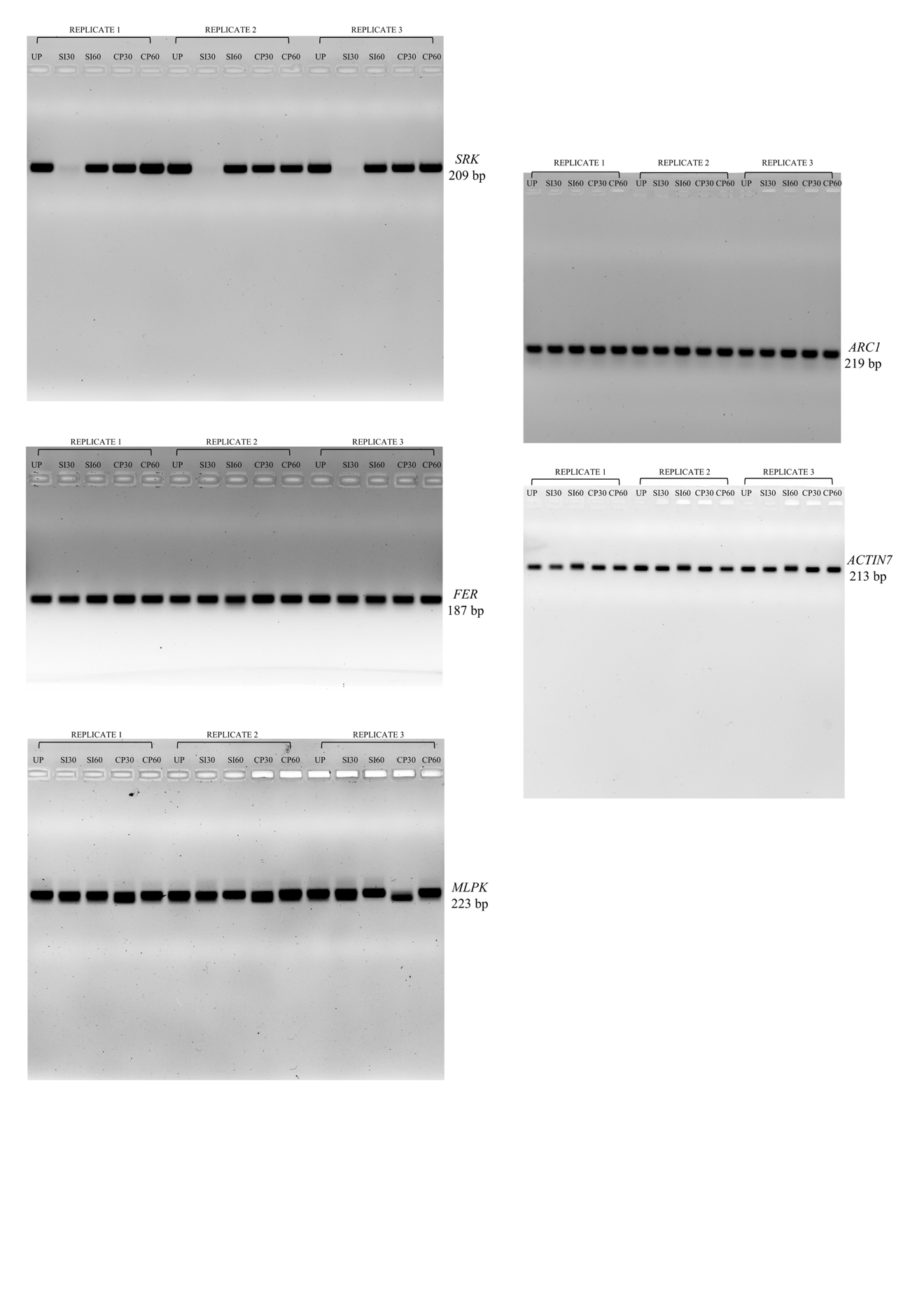
**

**Supplementary Figure S5**

**
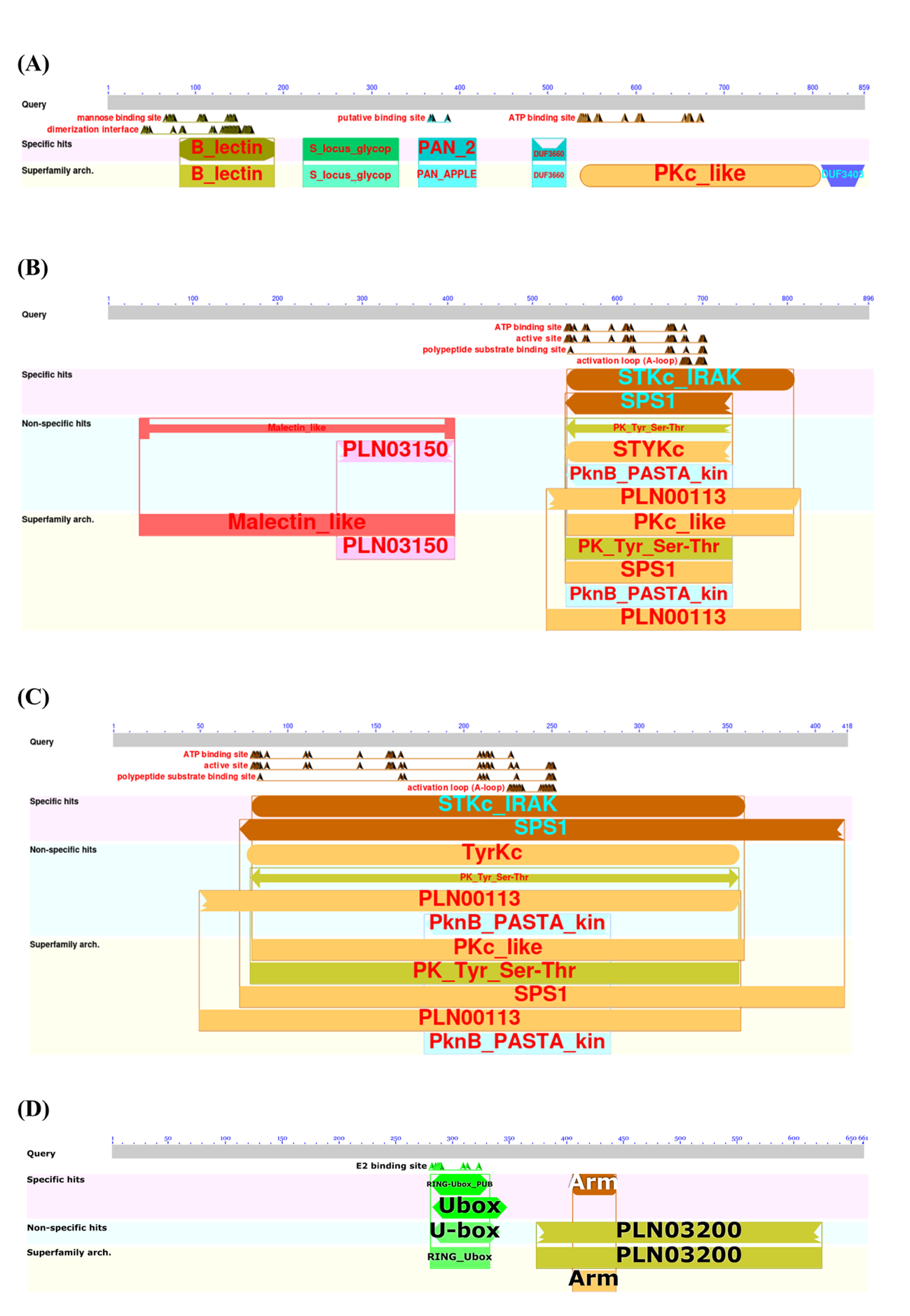
**

**Supplementary Figure S6**

**
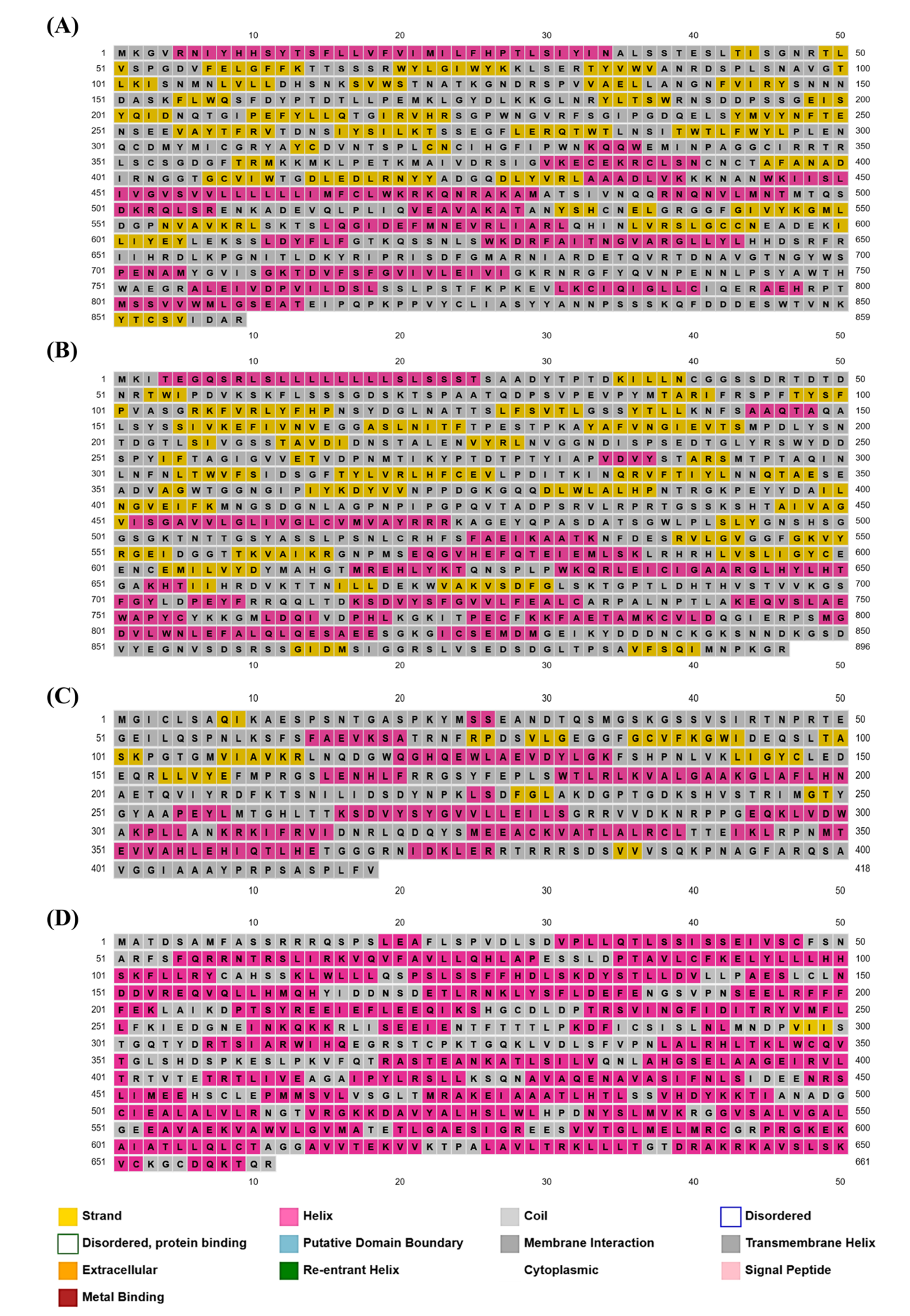
**

**Supplementary Figure S7**

**
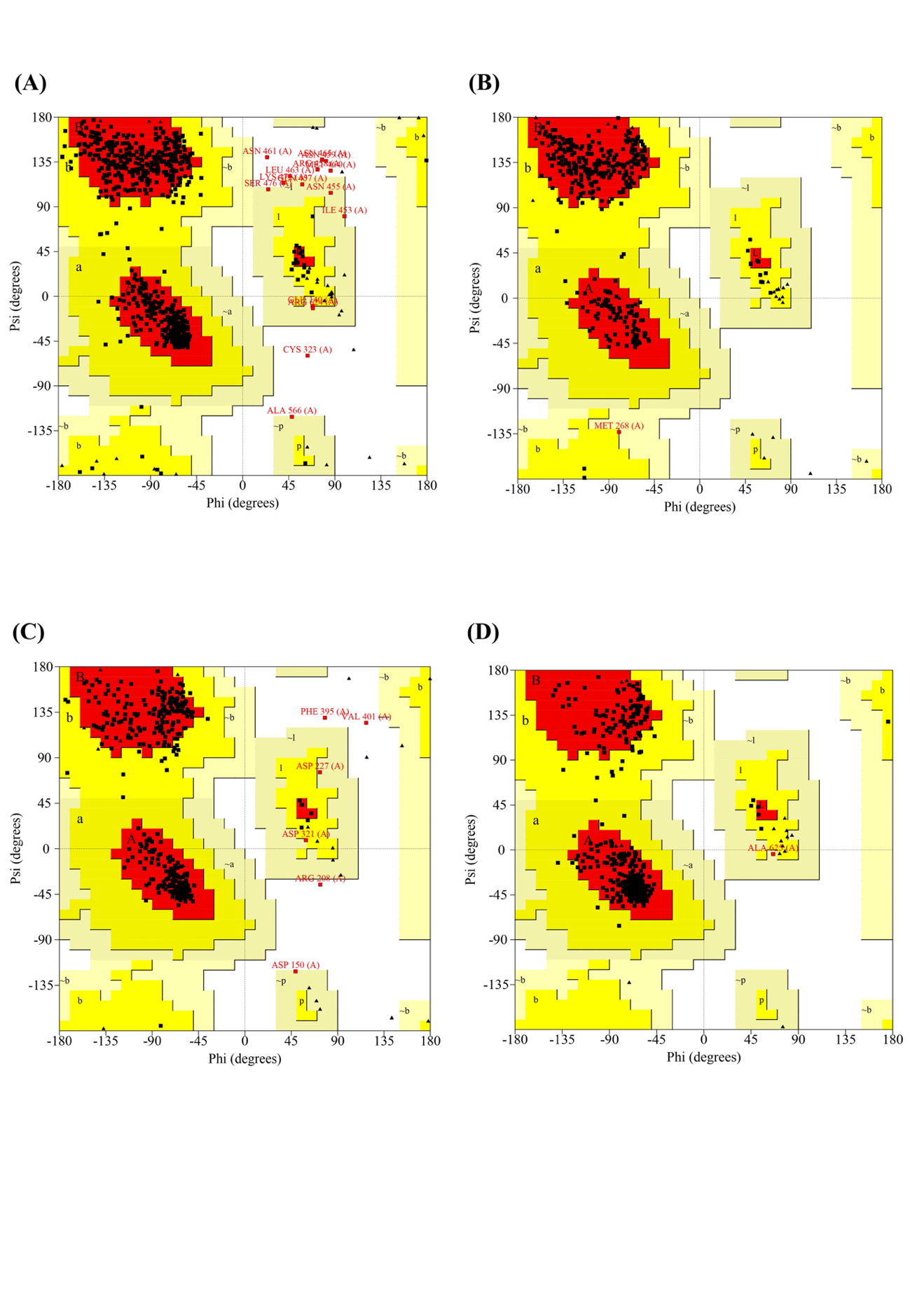
**

**Supplementary Figure S8**

**
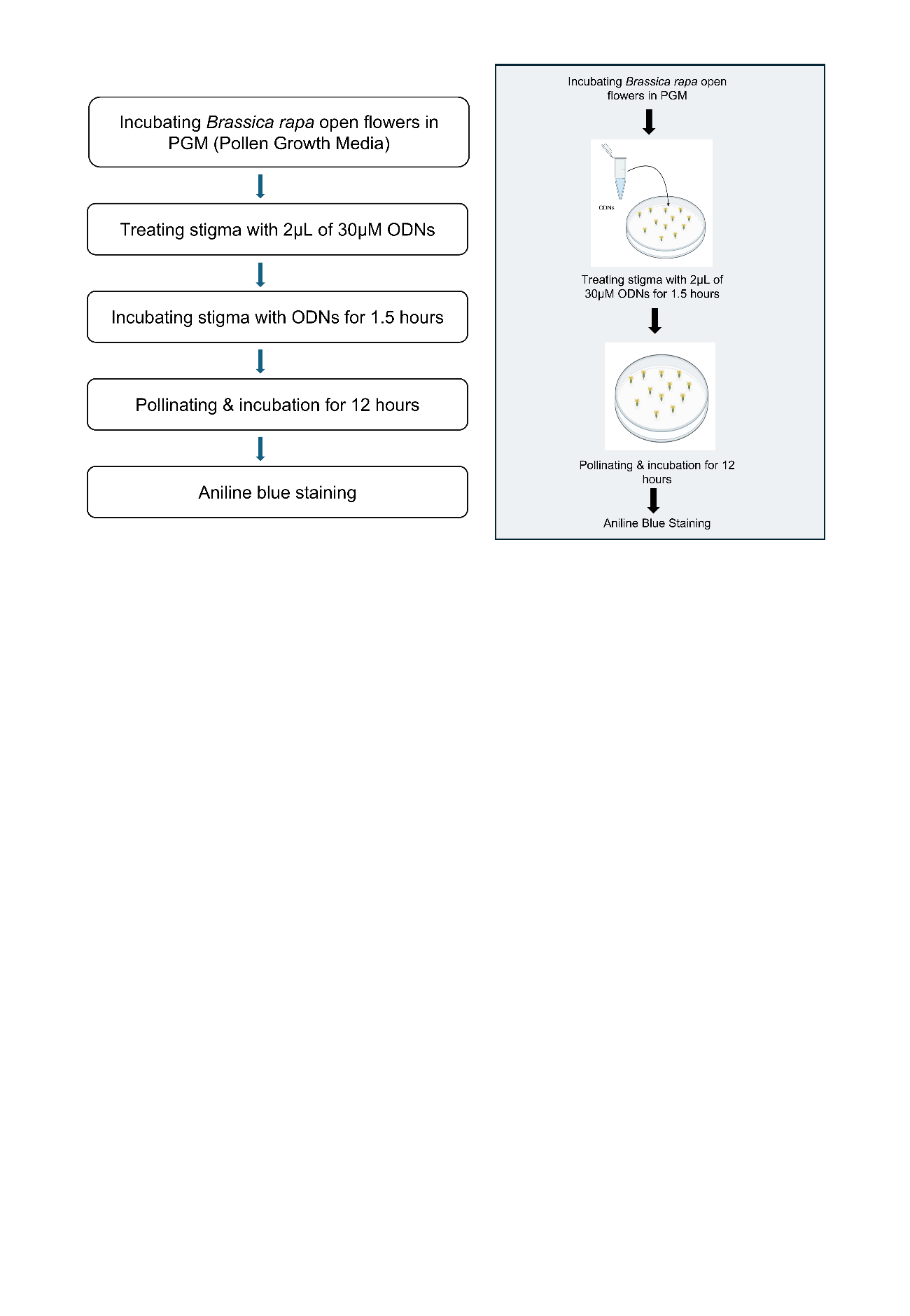
**

**Supplementary Figure S9**

**
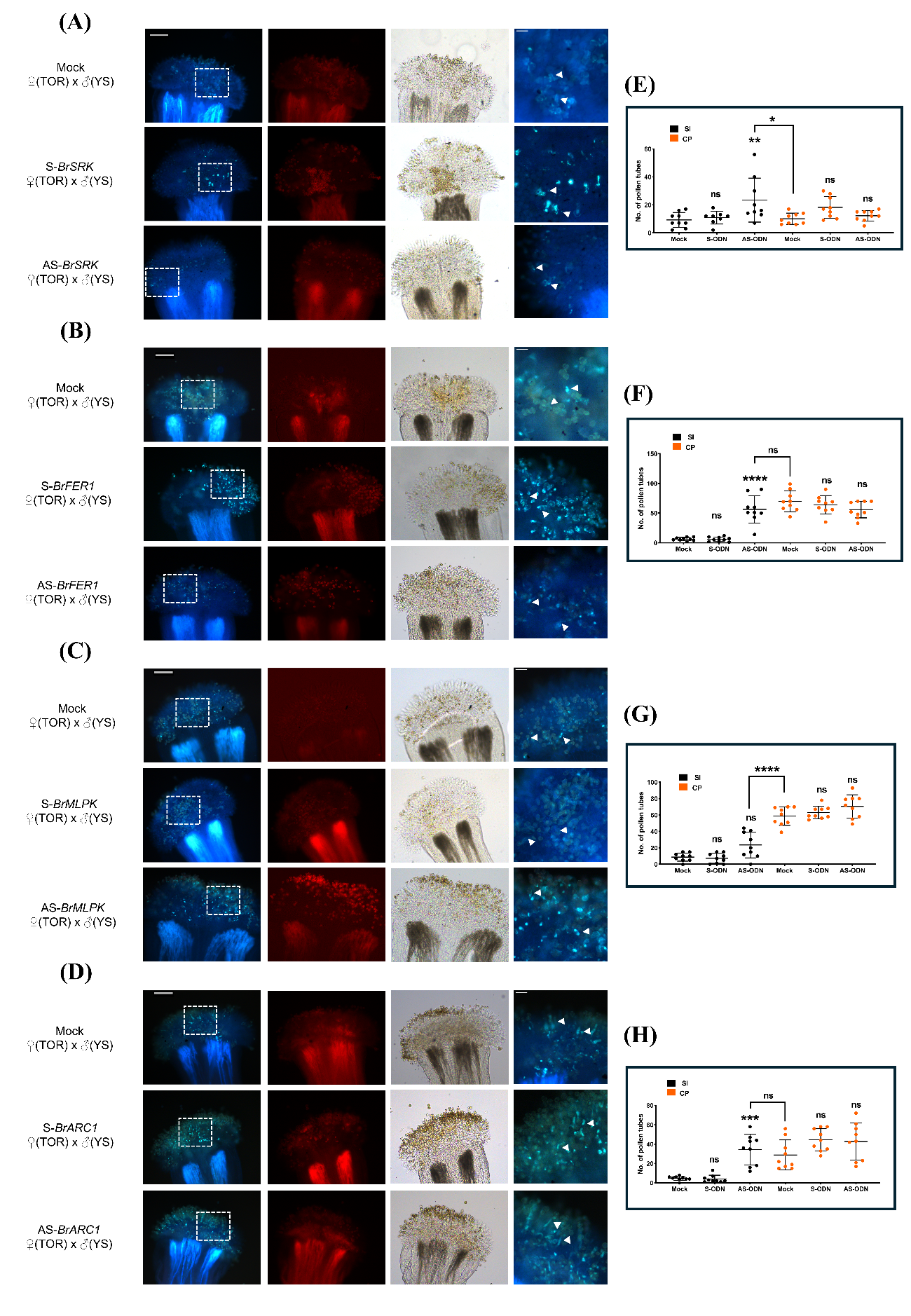
**

**Supplementary Figure S10**

**
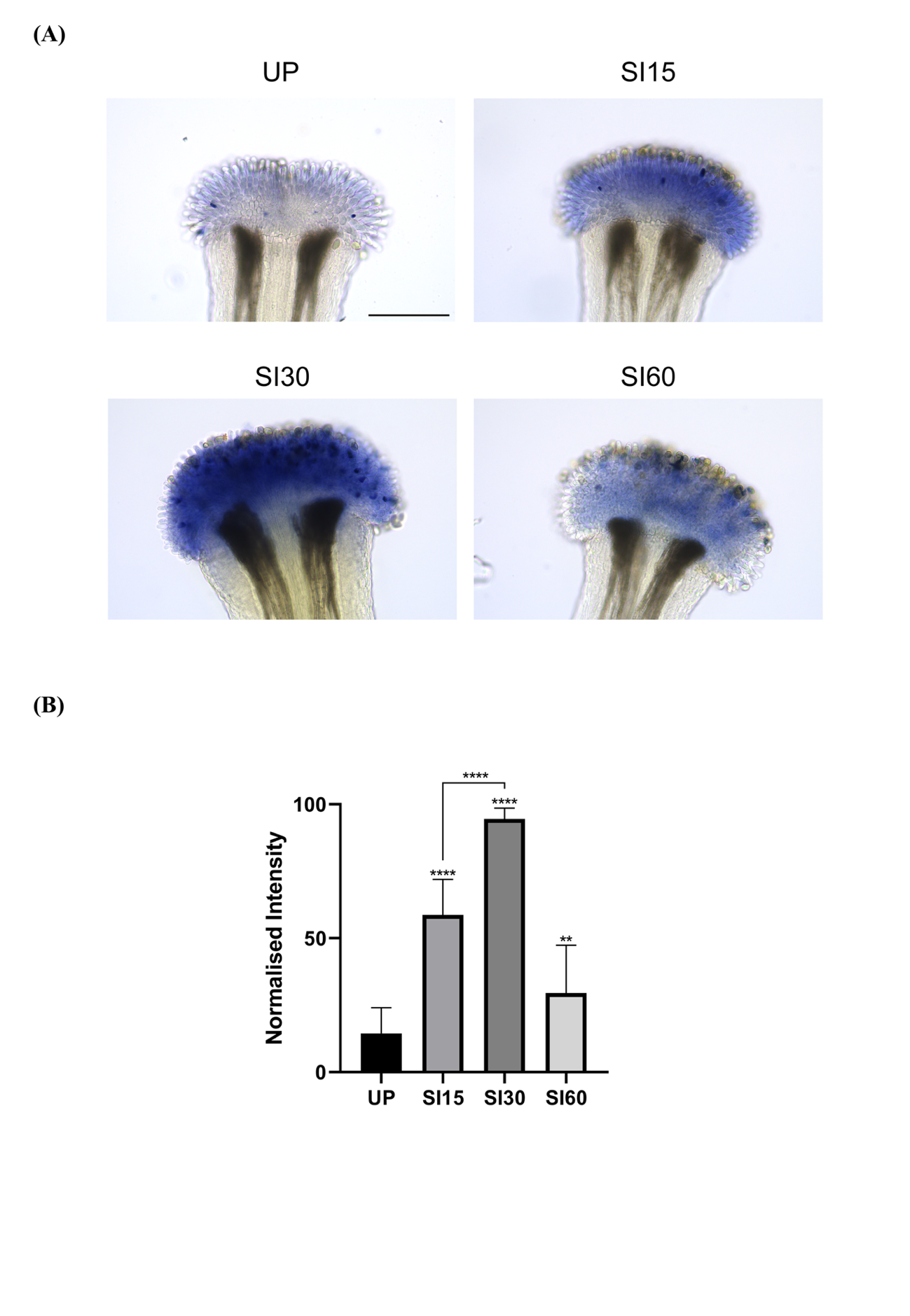
**

**Supplementary Figure S11**

**
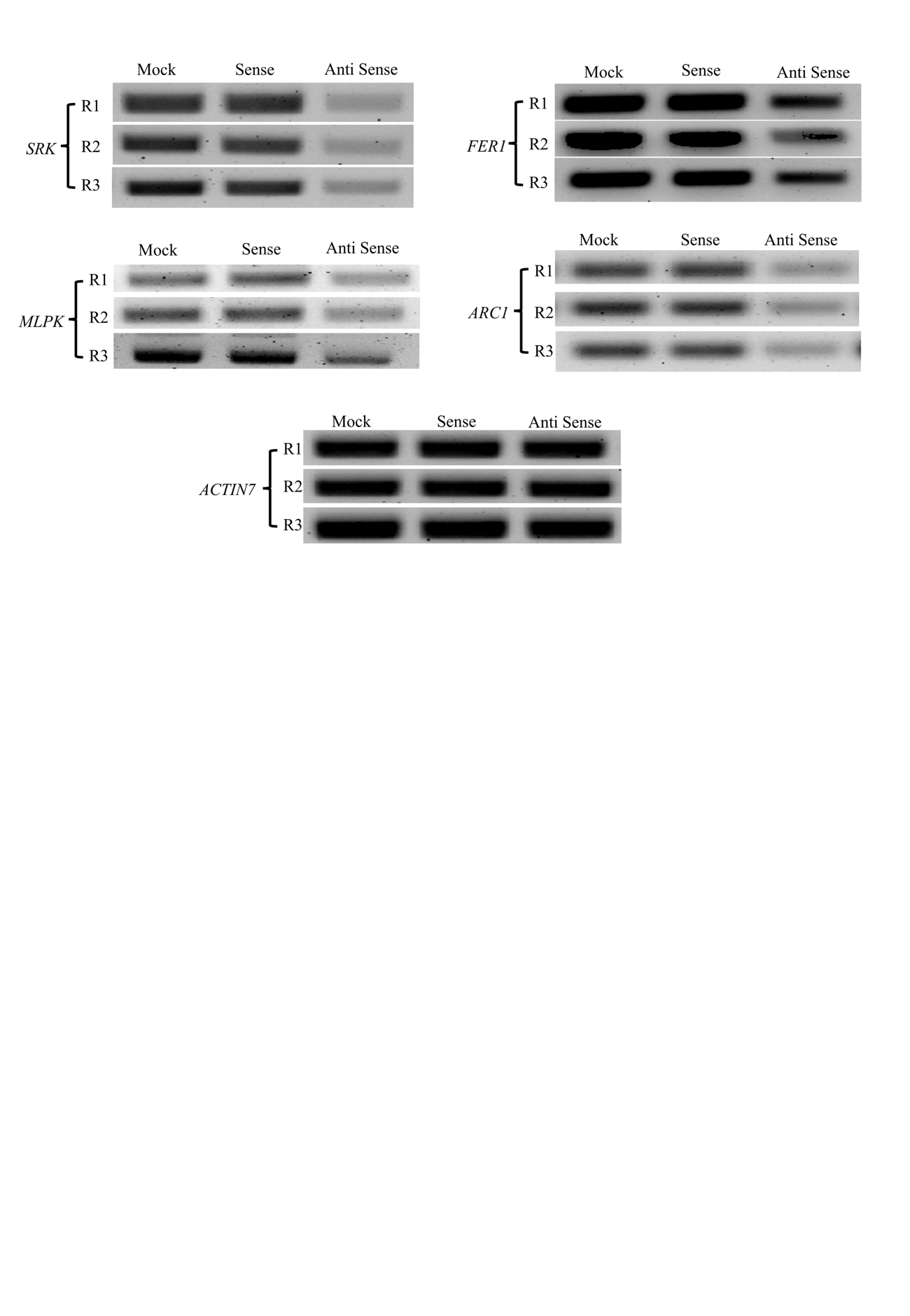
**

**Supplementary Figure S12**

**
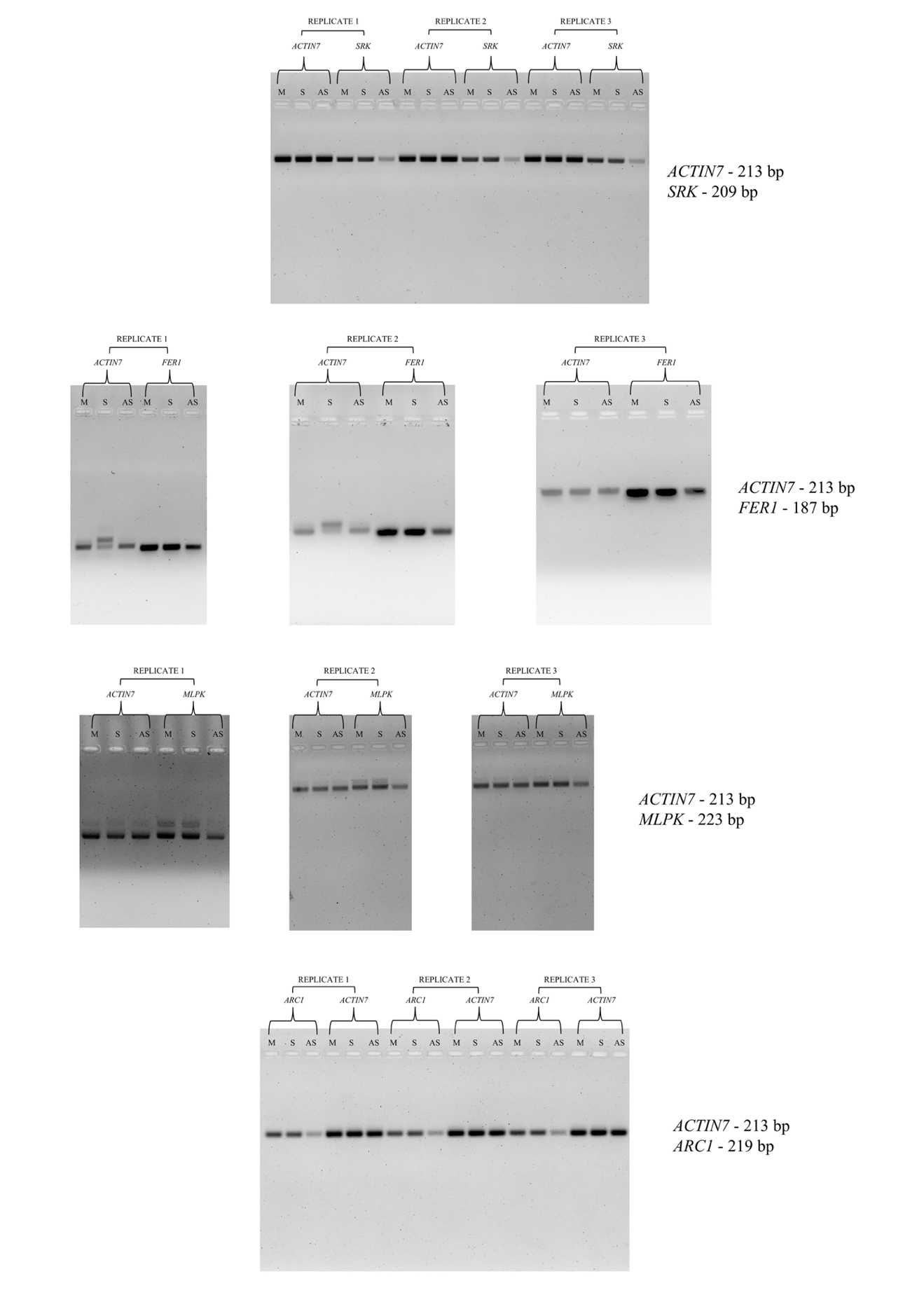
**
