# Supplementary material for: Characterization of self-incompatibility genes in Brassica rapa var. toria and yellow sarson
Source: Front Plant Sci. 2026 Jul 2;17:1857745. doi: 10.3389/fpls.2026.1857745 (PMC13372785; doi:10.3389/fpls.2026.1857745)

Supplemental Material - 3

Multiple sequence alignment of gene sequences of *SRK* selected for phylogenetic study

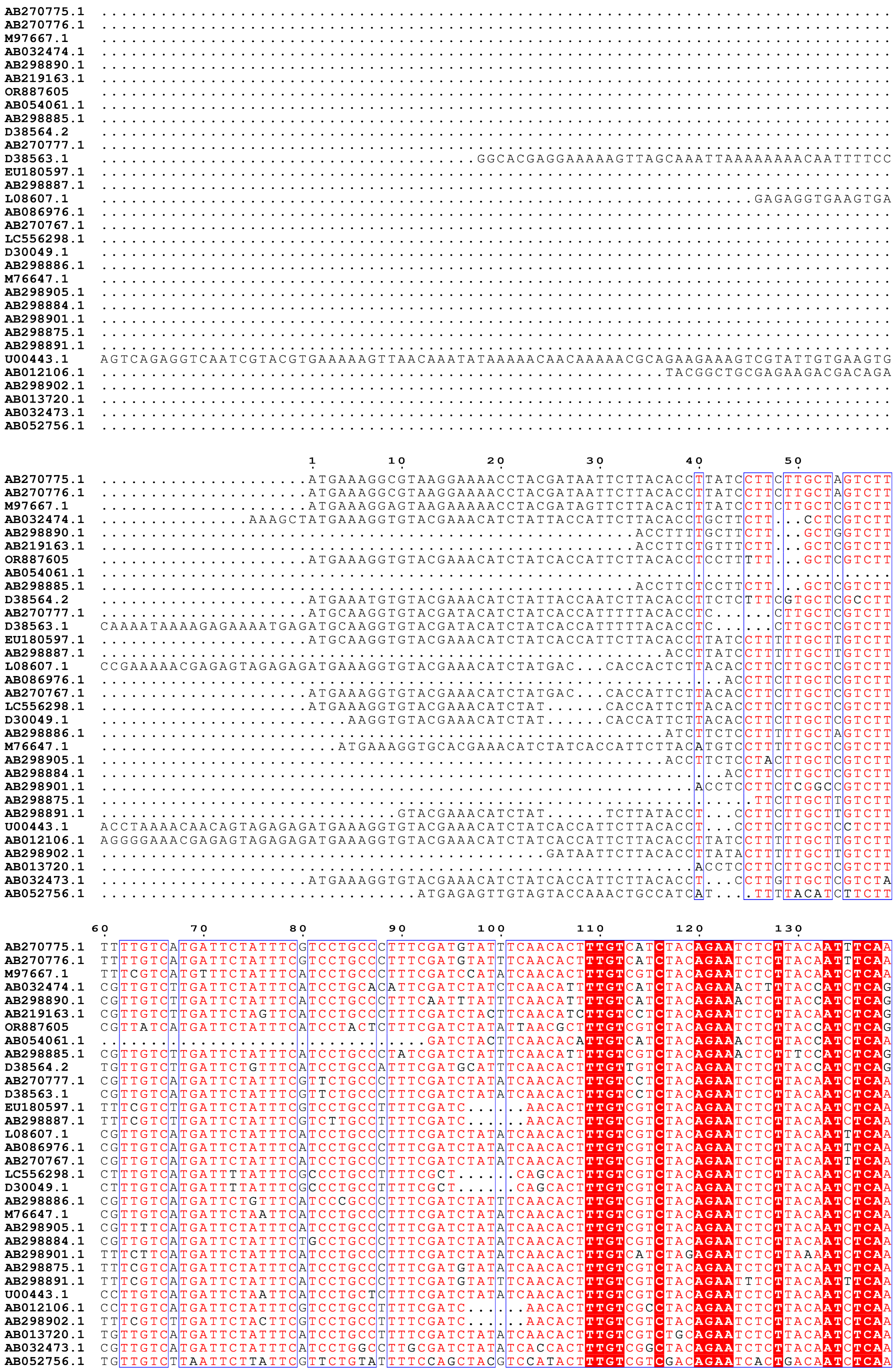

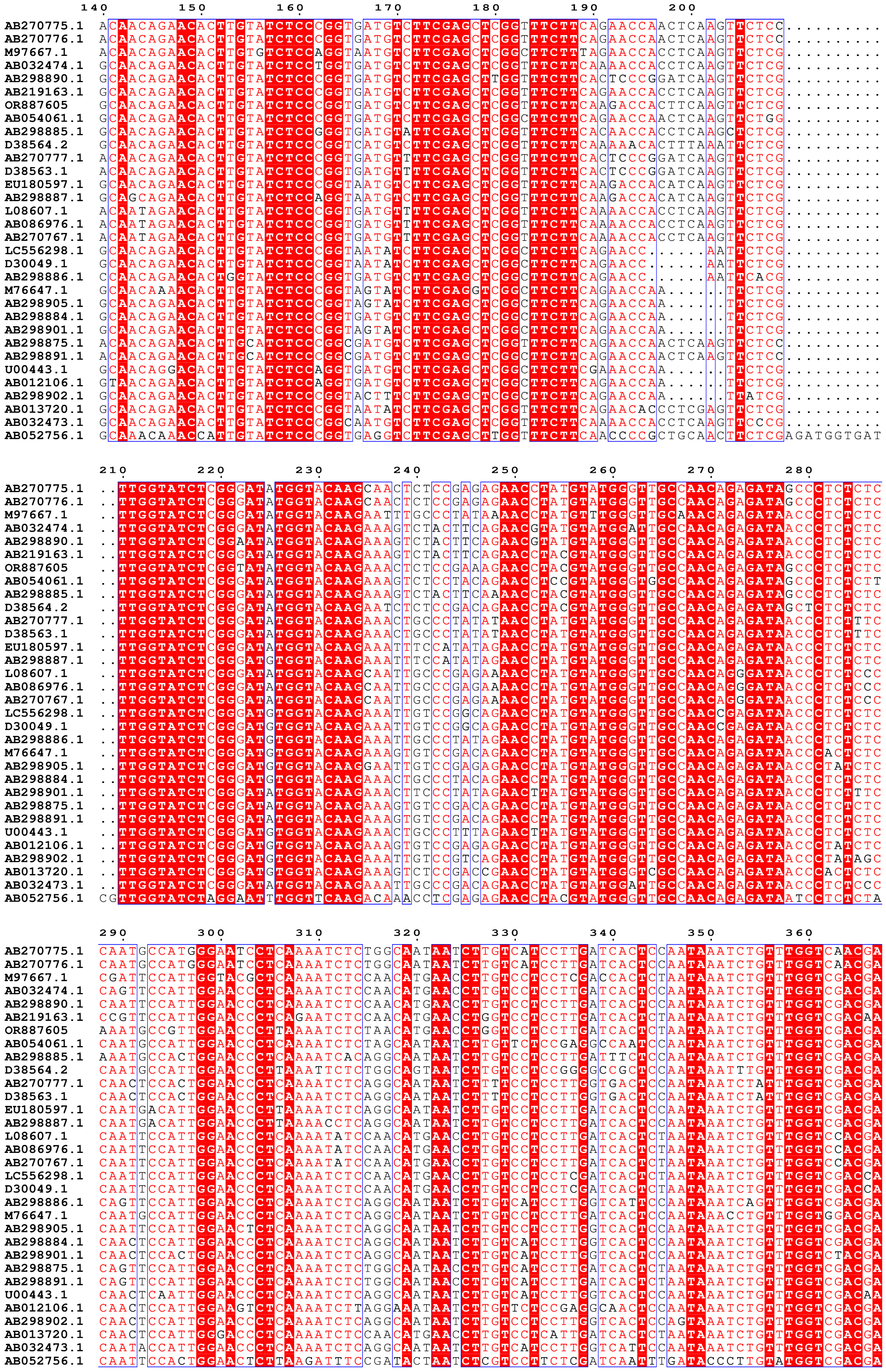

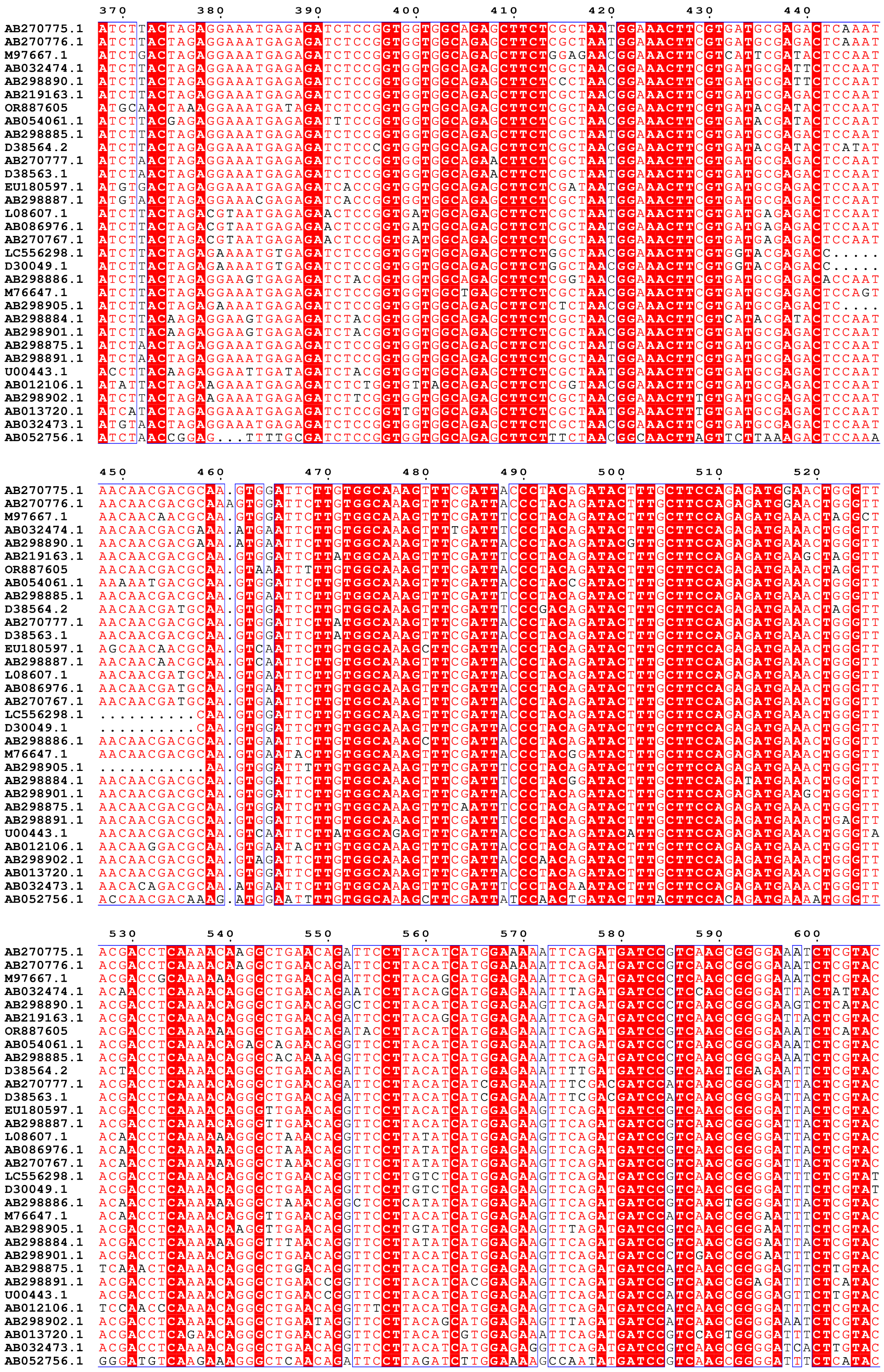

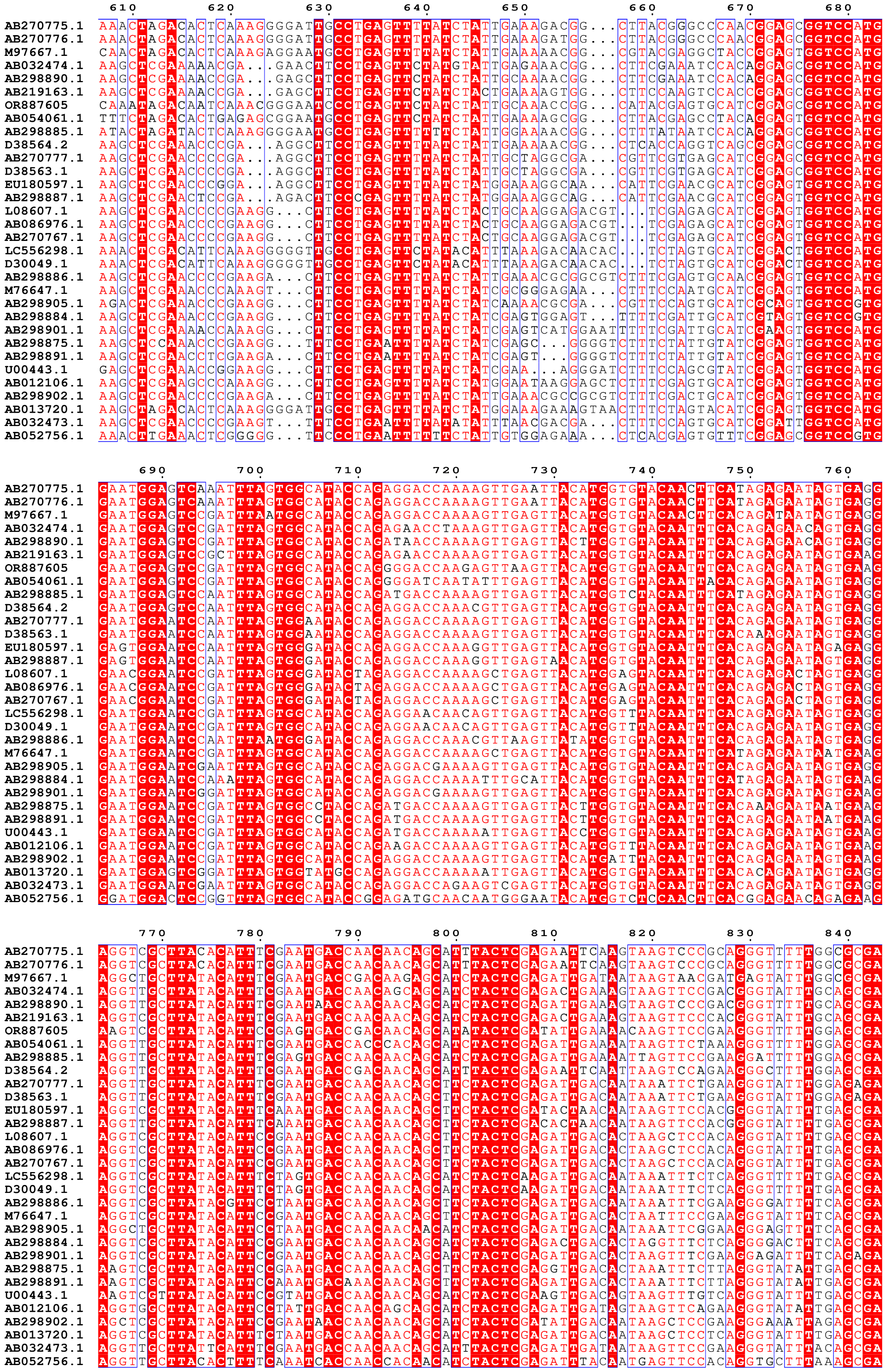

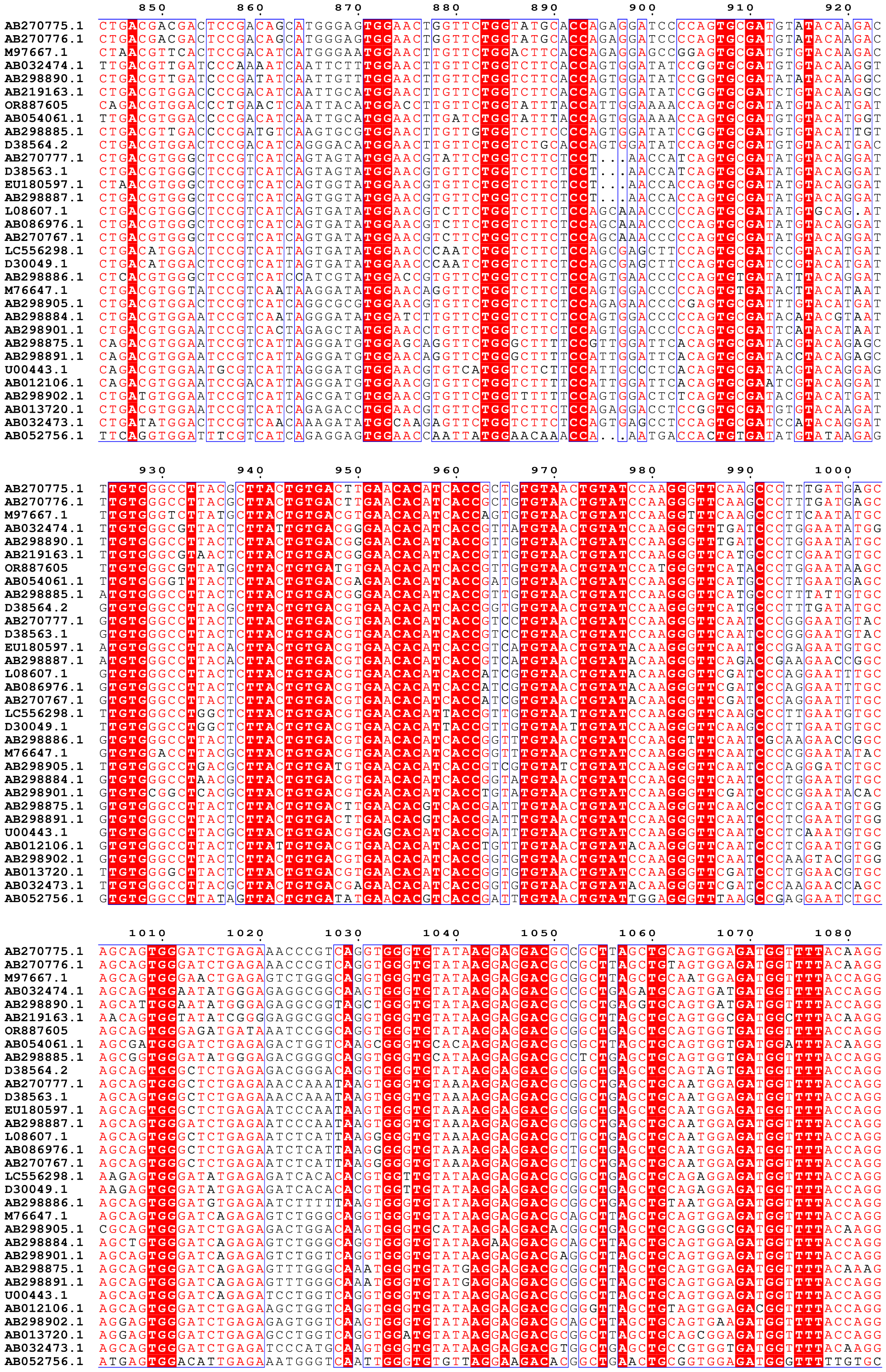

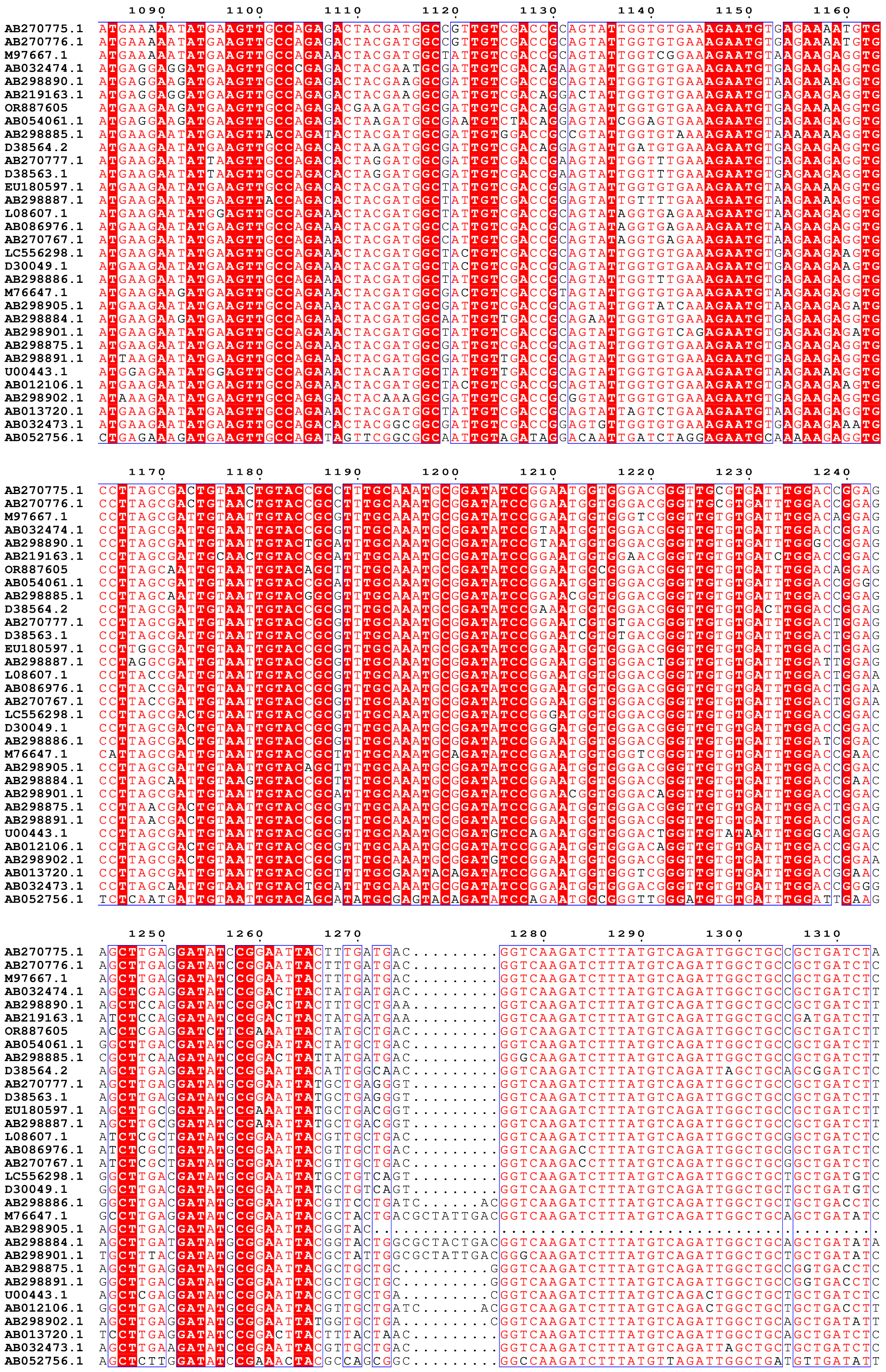

```
                  1320      1330      1340      1350      1360          1370
AB270775.1  GT...TAAAAAGAGAAACGCAAATGGTAAAACCATAACGTTGATTGTTGGAGTTGGTC...TTCTGT......TTAT...
AB270776.1  GT...TAAAAAGAGAAACGCAAATGGTAAAACCATAACGTTGATTGTTGGAGTTGGTC...TTCTGT......TTAT...
M97667.1    GT...TAAAAAGAGAAACGCGAATGGGAAAACCATAGCGTTGATTGTTGGAGTTTGTG...TTCTGC......TTCT...
AB032474.1  GT...TAAAAAGAGAAACGCGAATTGGAAAATCATAAGTTTGATTGTTGGAGTTACTGTTGTTCTGCTTCTGCTTCT...
AB298890.1  GT...TAAAAAGAGAAACGCGAATTGGAAAATCATAAGTTTGATTGTTGGAGTTAGTGTTGTTCTGCTTCTGCTTCT...
AB219163.1  GT...TAAAAAGAAAAACGCGAATTGGAAAATCATAAGTTTGATTGTTGGAGTTAGTGTTGTTCTGCTTCTGCTTCT...
OR887605    GT...TAAAAAGAAAAACGCGAATTGGAAAATCATAAGTTTGATTGTTGGAGTTAGTGTTGTTCTGCTTCTGCTTCT...
AB054061.1  GT...TAAAAAGAGAGACGCGAATTGGAAAATCATAAGTTTGATTGTTGGAGTTAGTGTTGTTCTGC......TTCT...
AB298885.1  GT...TCAAAAGAGAAACGCGAAAGGGAAAATCATAACTTTGATTGTTGGAGTTAGTG...TTCTGC......TTCT...
D38564.2    GT...TAAGAAGAGAAAAGCGAACGGTAAAATCATAAGTTTGATAGTCGGAGTTAGTG...TTCTGC......TTCT...
AB270777.1  GT...TAAAAAGAGCAACGCGAATTGGAAAATCATAAGTTTGATTGTTGGAGTTAGTGTTGTTCTGCTTCTGCTTCTGCT
D38563.1    GT...TAAAAAGAGAAACGGGAATTGGAAAATCATAAGTTTGATTGTTGGAGTTAGTGTTGTTCTGCTTCTGCTTCTGCT
EU180597.1  GT...TAAAAAGAGAAACGCGAATTGGAAAATCATAAGTTTGATTGTTGGAGTTAGTGTTGTTCTGCTTCTGCTTCT...
AB298887.1  GT...TAAAAAGAGAAACGGGAATTGGAAAATCATAAGTTTGATTGTTGGAGTTAGTGTTGTTCTGCTTCTGCTTCTGCT
L08607.1    GT...TAAGAAGAGTAACGCGAATGGGAAAATCATAAGTTTGATTGTTGGAGTTAGTG...TTCTGC......TTCT...
AB086976.1  GT...TAAGAAGAGTAACGCGAATGGGAAAATCATAAGTTTGATTGTTGGAGTTAGTG...TTCTGC......TTCT...
AB270767.1  GT...TAAGAAGAGTAACGCGAATGGGAAAATCATAAGTTTGATTGTTGGAGTTAGTG...TTCTGC......TTCT...
LC556298.1  GT...TGAGAAGAGAACCGCGAATGGGAAAATCGTAAGTTTGATTGTTGGAGTCTGTG...TTCTGC......TTCT...
D30049.1    GT...TGAGAAGAGAACCGCGAATGGGAAAATCGTAAGTTTGATTGTTGGAGTCTGTG...TTCTGC......TTCT...
AB298886.1  GT...TAAGAAGAGAAACGTGAATGTGAAAATCATAAGTTTGATTGTTGGAGTTAGTG...TACTGC......TTCT...
M76647.1    GC...TAAGAAGAGAAACGCGAGTGGGAAAATTATAAGTTTGACTGTTGGTGTTAGTG...TTCTGC......TTCT...
AB298905.1  ........GAGGAGAAACGCGAATGGGAAAATCATAAGTTTGATTATTGGAGTTAGTG...TTCTGC......TTCT...
AB298884.1  GC...TAAGAAGAGAAACGCGAATGGGAAAATCATAAGTGTGACTGTTGCTGTTAGTA...TTCTGC......TTCT...
AB298901.1  GC...TAAGAAGAGAAACGCGAATGGGAAAATCATAAGTTTGACTGTTGGTGTTAGTG...TTCTGC......TTCT...
AB298875.1  GT...TACGAAGAGAAACGCGAATTGGAAAATCATAAGTTTGGCTGTTGGAGTTAGTG...TTCTGC......TTCT...
AB298891.1  GT...TACGAAGAGAGACGCAAATTGGAAAATCATAAGTTTGACTGTTGGAGTTAGTG...TTCTGC......TTCT...
U00443.1    GT...TAAGAGGAGAAACGCGAATGGGCAAATCATAAGTTTGACTGTTGGAGTTAGTG...TTCTGC......TTCT...
AB012106.1  GT...TAAGAAGAGAAACGCCGATGGGAAAATCATAAGTTCGACTGTTGCAGTTAGTG...TTCTGC......TTCT...
AB298902.1  ATCGATAAGAAGGGAAACGTCAATGGGAAAATCATAAGTTTGACAGTTGGAGTTAGTG...TTCTGC......TTCT...
AB013720.1  GT...TAAAAAGAGAAACGCGAATGGGAAAATCATAAGTTTGATTGTTGGAGTTAGTG...GTCTGC......TCCT...
AB032473.1  GT...TAAGAAGAGAAACTCGAATGGGAAAATCATAGGTTTGATTGTTGGAGTTAGTG...TTCTGC......TTCT...
AB052756.1  GGA...GATGAAAGAAACATAAGAGGAAAAATCATCGGTTTAGCTGTTGGAGCTAGCGTTATCCTTT......TTCTGAG

                    1380      1390      1400      1410      1420      1430      1440
AB270775.1  .........TATGATCGTGTTCTGCCTCTGGAAAAGAAAACAAAAGCGAGGAAAAGAAATTGCAACATCTATTG......
AB270776.1  .........TATGATCGTGTTCTGCCTCTGGAAAAGAAAACAAAAGCGAGGAAAAGAAATTGCAACATCTATTG......
M97667.1    .........TATGATCATGTTCTGCCTCTGGAAAAGGAAACAAAAGCGAGCAAAAACAACTGCAACATCTATTG......
AB032474.1  .........TCTGATCATGTTCTGCCTTTGGAAAAGGAAACAAAATCGAGCAAAAGCAATGGCAACATCTATTG......
AB298890.1  .........TCTGATCATGTTCTGCCTTTGGAAAAGGAAACAAAATCGAGCAAAAGCAATGGCAACATCTATTG......
AB219163.1  .........TCTGATCGGGTTCTGCCTTTGGAAAAGGAAACAAAATCGAGCAAAAGCAATGGCAACATCTATTG......
OR887605    .........TCTGATCATGTTCTGCCTTTGGAAAAGGAAACAAAATCGAGCAAAAGCAATGGCAACATCTATTG......
AB054061.1  .........TATGATCATGTTCTGCCTTTGGAAAAAGAAACAAAATCGAGCAAAAGCAATGGCATCATCTATTG......
AB298885.1  .........TATTATCATGTTCTGCCTCTGGAAAAGGAAACAAAAGCGAGTAAAAGCAATGTCAGCATCTATTG......
D38564.2    .........TCTGATCATGTTCTGCCTCTGGAAAAGGAAAAAAAATCGAGCAAAAGCAAGTGCAACATCTATTG......
AB270777.1  .............ATCATGTTCTGCCTTTGGAAAAGGAAACAAAATCGAGCAAAAGCAATGGCAACATCTATTG......
D38563.1    TCT......TCTGATCATGTTCTGCCTTTGGAAAAGGAAACAAAATCGAGCAAAAGCAATGGCAACATCTATTG......
EU180597.1  .........TCTGATCATGTTCTGCCTTTGGAAAAGGAAACAAAATCGAGCAAAAGCAATGGCAACATCTATTG......
AB298887.1  TCTGCTTCTTCTGATCATGTTCTGCCTTTGGAAAAGGAAACAAAATCGAGCAAAAGCAATGGCAACATCTATTG......
L08607.1    .........TCTGATCATGTTTTGCCTCTGGAAAAGGAAACAAAATCGAGAAAAATCAAGTGCAGCATCTATTG......
AB086976.1  .........TCTGATCATGTTTTGCCTCTGGAAAAGGAAACAAAATCGAGAAAAATCAAGTGCAGCATCTATTG......
AB270767.1  .........TCTGATCATGTTTTGCCTCTGGAAAAGGAAACAAAATCGAGAAAAATCAAGTGCAGCATCTATTG......
LC556298.1  .........TCTTATATTTTTCTGCCTCTGGAAAAGGAAACAAAGGCGAGCAAAAGCAATGGCAACATCTATTG......
D30049.1    .........TCTTATATTTTTCTGCCTCTGGAAAAGGAAACAAAGGCGAGCAAAAGCAATGGCAACATCTATTG......
AB298886.1  .........TCTGATCATGTTTTGTCTCTGGAAAAGGAAACAAAATCGAGCAAAAGCAAGTGCAGCATCAATTG......
M76647.1    .........GCTGATCATGTTCTGCCTCTGGAAAAGAAAACAAAAGCGAGCAAAAGCAAGTGCAATATCCATTG......
AB298905.1  .........TCTGATTCTGTTCTGGCTCTGGAAAAGGAAACAAAAGCGAGCAAAAGCAAGTGCAGTATCTATTGAAACAG
AB298884.1  .........TCTGATCATGTTCTGCCTCTGGAAAAGAAAACAAAAGCGAACAAAATCAAGTTCAACATCTATTG......
AB298901.1  .........TCTGGTCATGTTCTGCCTCTGGAAAATAAAACAAAAGCGAGCAAAAGCAAGTGCAACATCTATTG......
AB298875.1  .........TCTGATCATTTTCTGCGTCTGGAAAAGAAAACAAAAGCAAGCAAAAGCAAAAGCAACATCTATTG......
AB298891.1  .........TCTGATCATGTTCTGCCTCTGGAAAAGAAAACAAAAACAAGCAAAAGCAA......CATCTATTG......
U00443.1    .........TCTAATCATGTTCTGCCTCTGGAAAAGAAAACAAAAGCGAGCAAACGCAAATGCAACATCTATCG......
AB012106.1  .........TCTGATCATGTTCTGCCTCTGGAAAAGAAAACAGAAGCGAGCAAAAGCAAGTGCAACATCTATTG......
AB298902.1  .........TCTGATCATCTTCTGCCTCTGGAAAAGAAAGCATAAACGAGCAGAAGCAAGTGCAACATCTATTG......
AB013720.1  .........TCTGATCATGTTCTGCATCTGGAAAACGAAACAAAAGCGAGTAAAAGGAAGTGCAATATCTATTG......
AB032473.1  .........TCTAATAATTTCCTGCCTCTGGAAAAGGAGACAAAAGCGAGCAAAAGCAAGTGCAACATCTATTG......
AB052756.1  .........TTCCATCATGTTCTGCGTTTGGAGAAGGAAACAGAAGCTACTAAGAGCAACTGAAGCACCTATTG......

                1450      1460      1470         1480      1490      1500      1510
AB270775.1  TAAATCGACAGAGAAACCACGATGTGCTTATAA...ACGGGATGATACTATCAAGCAAGAGACAGTTGCCTA...GAGAG
AB270776.1  TAAATCGACAGAAAAACCACGATGTGCTTATAA...ACGGGATGATACTATCAAGCAAGAGACAGTTGCCTA...GAGAG
M97667.1    TAAATCGACAGAGAAACCAAGATTTGCTAATGA...ACGGGATGATACTATCAAGCAAGAGACAGTTGCCTA...TAGAG
AB032474.1  TCAATCAACAGAGAAACCAAAATGTACTTATGATGAACGGGATGACACAATCAAACAAGAGACAGTTGTCAA...GAGAG
AB298890.1  TCAATCAACAGAGAAACCAAAATGTACTTATGA...ACGGGATGACACAATCAAACAAGAGACAGTTGTCAA...GAGAG
AB219163.1  TCAATCAACAGAGAAACCAAAATGTACTTATGA...ACACGATGACACAATCAGACAAGAGACAGTTGTCTA...GAGAG
OR887605    TCAATCAACAGAGAAACCAAAATGTACTTATGA...ACACGATGACACAATCAGACAAGAGACAGTTGTCTA...GAGAG
AB054061.1  TAAATCACCAGAGAAACCAAAATGTACTTATGA...ACACGATGACACAATCAAACAAGAGACAGTTGTCTA...GAGAG
AB298885.1  TCAATGGACAGAGAAACCAAAATGTAATTATGA...ACGGGATGACACAATCAAGCAAGACACAGTTGTCTATTAGAGAG
D38564.2    ACAATCAACAGAGAAACCAAAATGTACTTATGA...ACGGGATGACACAATCAAACAAGAGACAGTTGTCTA...GAGAG
AB270777.1  TCAATCAACAGAGAAACCAAAATGTACTTATGA...ACACGATGACACAATCAAACAAGAGACAGTTGTCTA...GAGAG
D38563.1    TCAATCAACAGAGAAACCAAAATGTACTTATGA...ACACGATGACACAATCAAACAAGAGACAGTTGTCTA...GAGAG
EU180597.1  TCAATCAACAGAGAAACCAAAATGTACTTATGA...ACGGGATGACACAATCAAACAAGAGACAGTTGTCAA...GAGAG
AB298887.1  TCAATCAACAGAGAAACCAAAATGTACTTATGA...ACACGATGACACAATCAAACAAGAGACAGTTGTCTA...GAGAG
L08607.1    CAAATCGACAGAGAAACCAAAATTTGCCTATGA...ACGGGATAGTACGATCAAGCAAGAGACAGTTGTCTG...GAGAG
AB086976.1  CAAATCGACAGAGAAACCAAAATTTGCCTATGA...ACGGGATAGTACTATCAAGCAAGAGACAGTTGTCTG...GAGAG
AB270767.1  CAAATCGACAGAGAAACCAAAATTTGCCTATGA...ACGGGATAGTACTATCAAGCAAGAGACAGTTGTCTG...GAGAG
LC556298.1  TCCATCGACAGAGAAAACAAATTTTGCTTATGA...ACGGGATGACACTATCAAACAATAGACAGTTGTCTA...GAGAG
D30049.1    TCCATCGACAGAGAAAACAAATTTTGCTTATGA...ACGGGATGACACTATCAAACAATAGACAGTTGTCTA...GAGAG
AB298886.1  CTAATCGACAGAGAAACCAAAATTTGCCTATGA...AGAAGATGGTACTATCAAGCAAGAGACAGTTGTCTG...GAGAG
M76647.1    CAAATACACAGAGAAACCAAAACTTGCCTATGA...ACGAGATGGTACTATCAAGCAAGAGAGAGTTTTCTG...GAGAG
AB298905.1  CAAATCGACAAAGAAACCAAAATTTGCCTATGA...ACGGGATAGTACTATCAAGCAAGAGACAGTTGTCTG...GAGAG
AB298884.1  CAAATCGACAAAGAAACCAAAATTTGCCAATGA...ACGGGATGGTACTATCAAGCAAGCAAGAGTTTTCTG...GAGAG
AB298901.1  CAAATCGACAGAGAAATCAAAACTTGCTTATGA...ACGGGATGGTACTATCAAGCAAGAGAGAGTTTTCTG...GAGAG
AB298875.1  CAAATCGACAGAGAAACCAAAACTTGCCTATGA...ACGGGATGGTACTATCAACCAAGAGAGAGTTTCCTG...GAGAG
AB298891.1  AAAATCGACAGAGAAACCAAAACTTGCCTATGA...ATGGGATGGTACTATCAACCAAGAGAGAGTTTCCTG...GAGAG
U00443.1    CAAATCGACAGAGAAACCAAAACTTGCCTATGA...ACGGGATGGTACTATCAAGCAAGAGAGAGTTTCTTG...AGGAG
AB012106.1  CAAATCGACAGAGAAACCAAAACTTGTCTATGA...ACGGGATGGTACTATTAAGCAAGAGAGAGTTTTCTG...TAAAG
AB298902.1  CAAATCGACAGGGAAACCAAACCTTGCCTATGA...ACGGGATGGTACTATCAAGCAAGAAAGAGTTTTCTG...GAAAG
AB013720.1  CAAATCGAGAGAGAAGCCAAAATTTGCCGATGA...CCGGGATGGTACTATCAAGCAAGACACAGTTGTCCG...GAGTG
AB032473.1  CAAATCGACAGAGAAACCAAAATATGCCTATGA...ACGGGATGGTGCTATCAAGCAAGAGACAGTTGTCTG...GAGAG
AB052756.1  TGTATCCAACAATAAACCAAGGTTTGCTAATGA...ATCGCCTGGAAATATCAAGCGGGAGACACCTATCTG...AAGAT
```

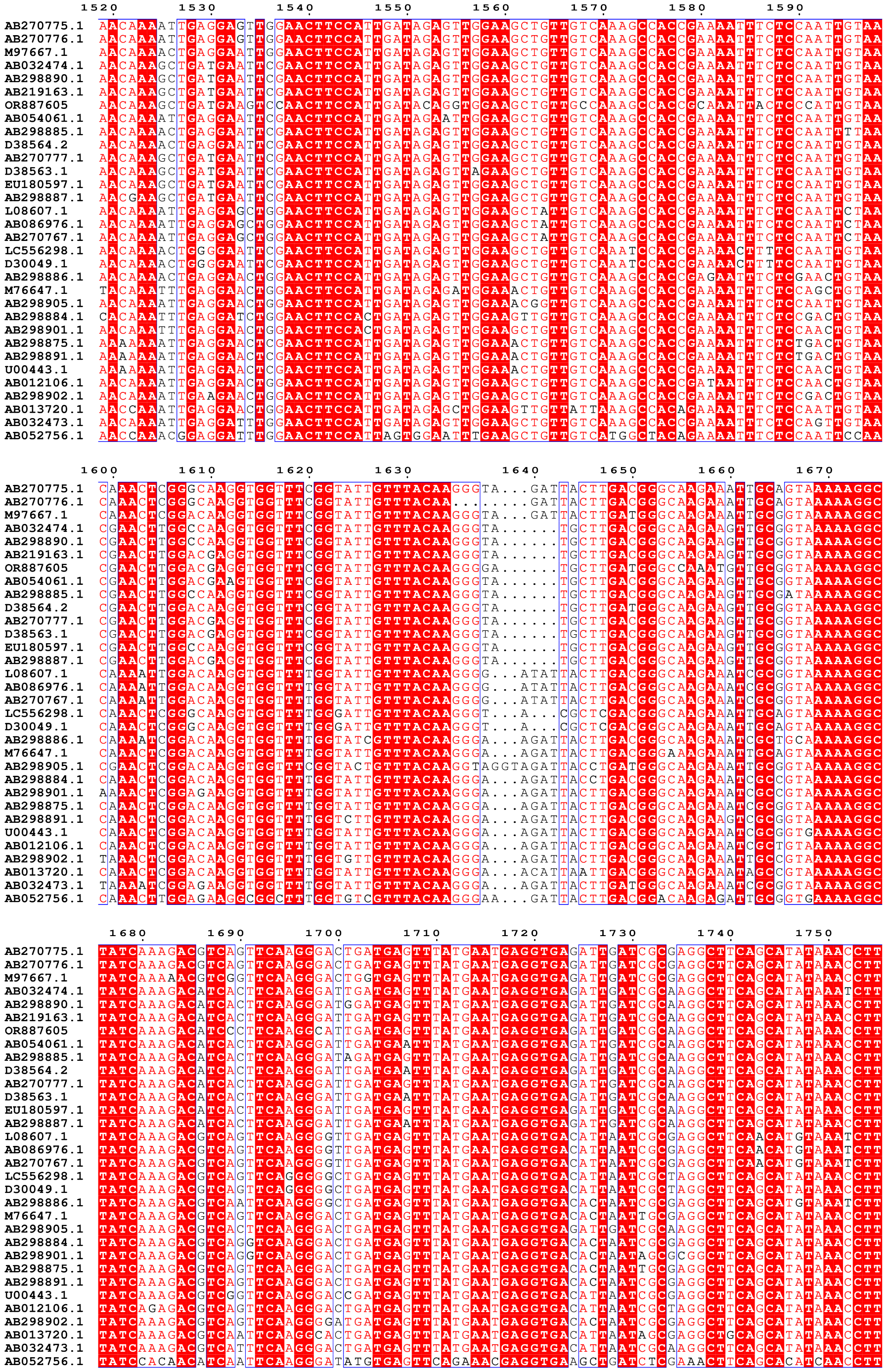

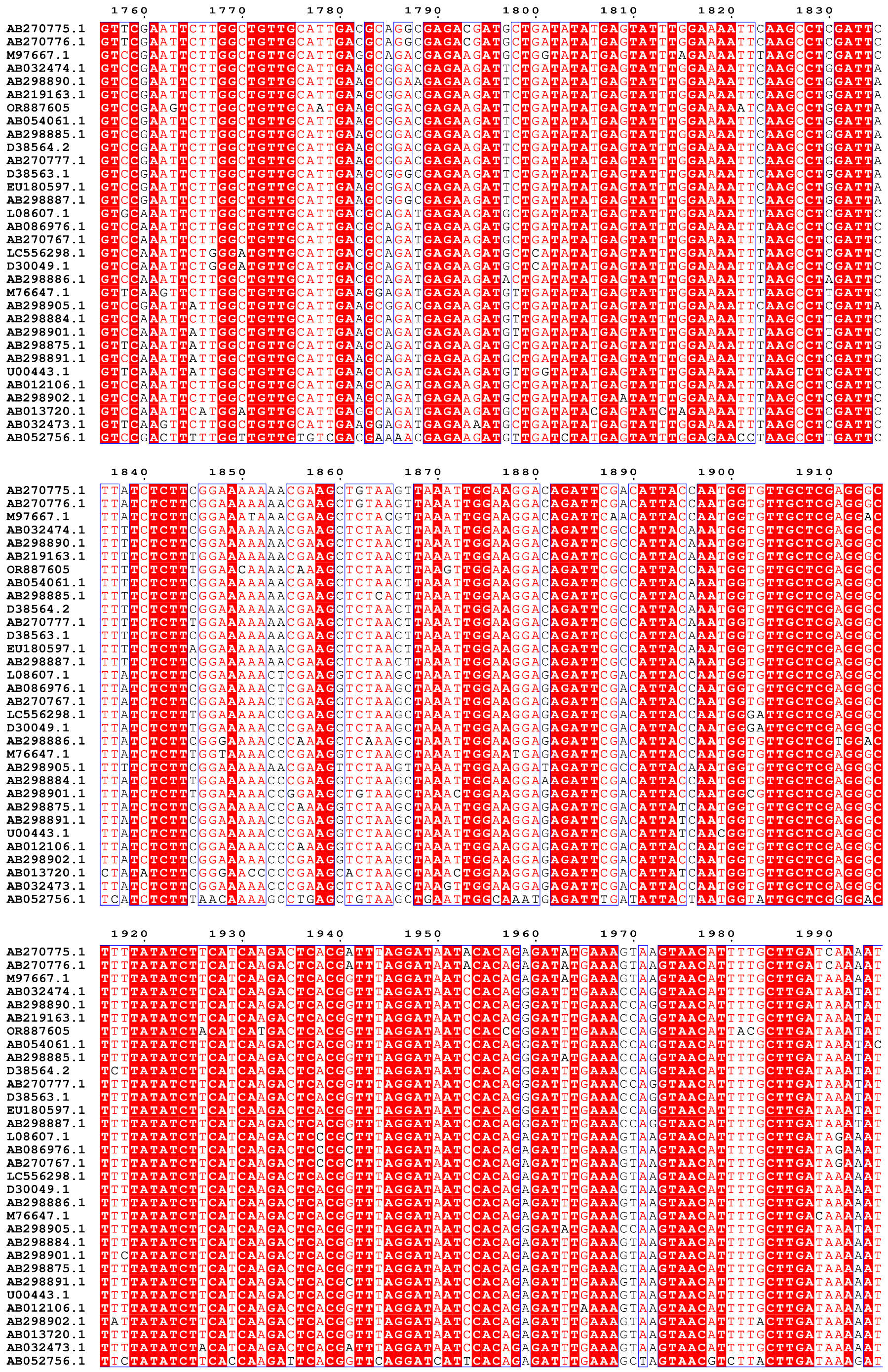

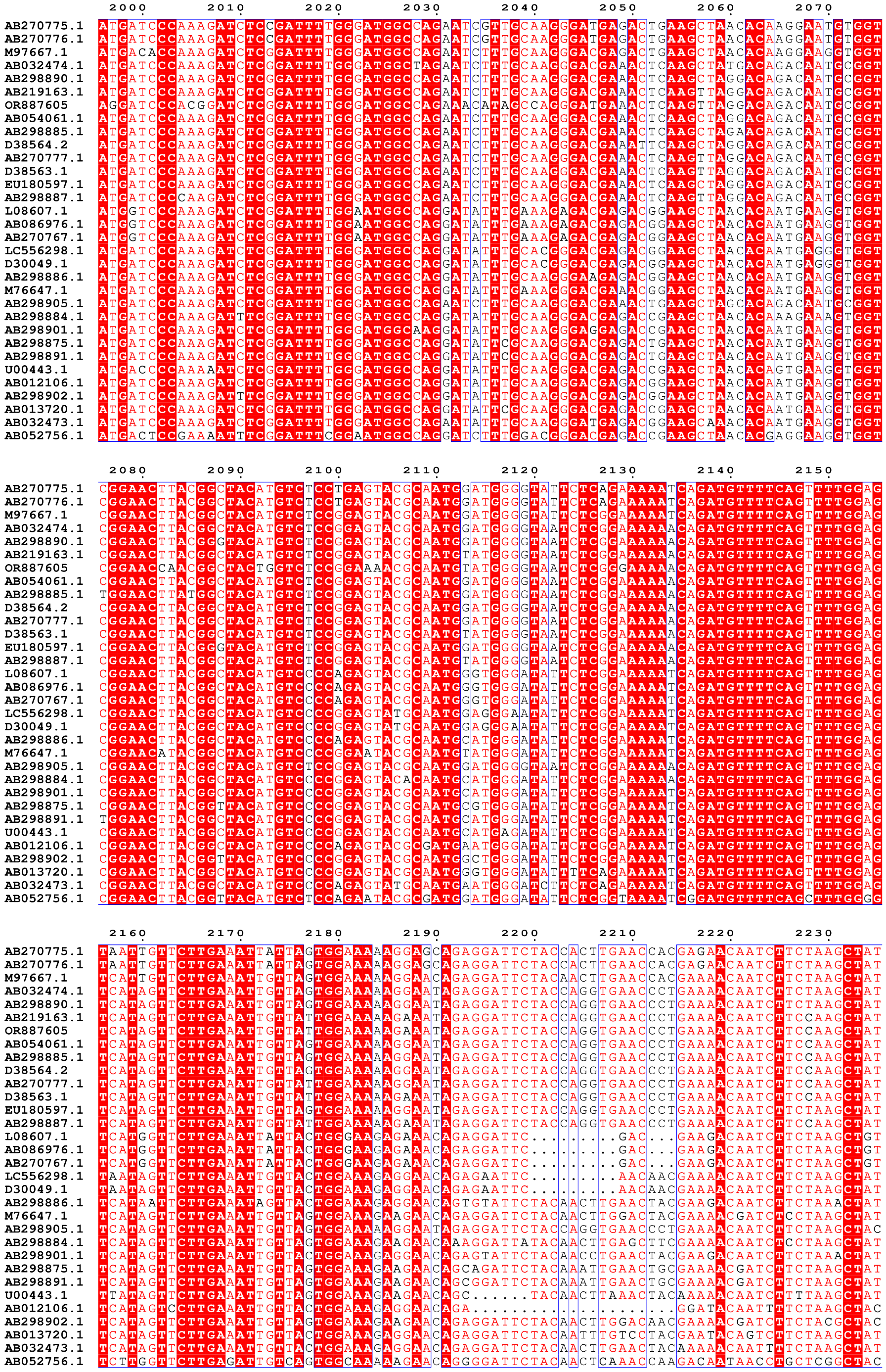
2000 2010 2020 2030 2040 2050 2060 2070
2080 2090 2100 2110 2120 2130 2140 2150
2160 2170 2180 2190 2200 2210 2220 2230
AB270775.1
AB270776.1
M97667.1
AB032474.1
AB298890.1
AB219163.1
OR887605
AB054061.1
AB298885.1
D38564.2
AB270777.1
D38563.1
EU180597.1
AB298887.1
L08607.1
AB086976.1
AB270767.1
LC556298.1
D30049.1
AB298886.1
M76647.1
AB298905.1
AB298884.1
AB298901.1
AB298875.1
AB298891.1
U00443.1
AB012106.1
AB298902.1
AB013720.1
AB032473.1
AB052756.1

```
                 2240      2250      2260      2270      2280      2290      2300      2310
AB270775.1  GTATGGAGTCATTGGACGGAGGGAAGAGCGCTAGAAATCGTAGATCCAGTCATTGTAGATTCATTGTCATCACTGGCAGC
AB270776.1  GTATGGAGTCATTGGACGGAGGGAAGAGCGCTAGAAATCGTAGATCCAGTCATTGTAGATTCATTGTCATCACTGGCAGC
M97667.1    GTATGGAGTCACTGGACGGAGGGAAGAGCGCTAGAAATTGTTGATCCAGTCATCGTAGATTCATTGTCATCATTACCAGC
AB032474.1  GCATGGAGTCATTGGGCGGAGGGAAGAGCGCTAGAAATCGTAGATCCAGTCATCGTAGATTCATTGTCATCTCTGCCATC
AB298890.1  GCATGGAGTCATTGGGCGGAGGGAAGAGCGCTAGAAATCGTAGATCCAGTCATCGTAGATCCATTGGCATCTCTGCCATC
AB219163.1  GCATGGACTCATTGGGCGGAGGGAAGAGCGCTAGAAATCGTAGATCCAGTCATCTTAGATTCATTGTCATCTCTGCCATC
OR887605    GCATGGACTCATTGGGCGGAGGGAAGAGCGCTAGAAATCGTAGATCCAGTCATCTTAGATTCATTGTCATCTCTGCCATC
AB054061.1  GCATGGACTCATTGGGCGGAGGGAAGAGCGCTAGAAATCGTAGATCCAGTCATCGTAGATTCATTGTCATCTCTGCCATC
AB298885.1  GCATGGACTCATTGGGCGGAGGGAAGAGCGCTAGAAATCGTAGATCCAGTCATCGTAGATTCATTGTCATCTCTGCCATC
D38564.2    GTATGGACTCATTGGGCGGAGGGAAGAGCGCTAGAAATCGTAGATCCAGTCATCTTAGATTCATTGTCATCTCTGCCATC
AB270777.1  GCATGGACTCATTGGGCGGAGGGAAGAGCGCTAGAAATCGTAGATCCAGTCATCGTAGATTCATTGTCATCTCTGCCATC
D38563.1    GCATGGACTCATTGGGCGGAGGGAAGAGCGCTAGAAATCGTAGATCCAGTCATCTTAGATTCATTGTCATCTCTGCCATC
EU180597.1  GCATGGAGTCATTGGGCGGAGGGAAGAGCGCTAGAAATCGTAGATCCAGTCATCGTAGATCCATTGGCATCTCTGCCATC
AB298887.1  GCATGGACTCATTGGGCGGAGGGAAGAGCGCTAGAAATCGTAGATCCAGTCATCTTAGATTCATTGTCATCTCTGCCATC
L08607.1    GCATGGAGAAATTGGAAGGAAGGAAGAGCGCTAGAAATAGTAGATCCAGTCATCGTAAATTCATTTTCACCACTGTCATC
AB086976.1  GCATGGAGAAATTGGAAGGAAGGAAGAGCGCTAGAAATAGTAGATCCAGTCATCGTAAATTCATTTTCACCACTGTCATC
AB270767.1  GCATGGAGAAATTGGAAGGAAGGAAGAGCGCTAGAAATAGTAGATCCAGTCATCGTAAATTCATTTTCACCACTGTCATC
LC556298.1  GCATGGAGTAATTGGAAGGAAGGAAGAGCGCTAGAAATCGTAGATCCAGATATCGTAGATTCACTTTCACCACTGTCATC
D30049.1    GCATGGAGTAATTGGAAGGAAGGAAGAGCGCTAGAAATCGTAGATCCAGATATCGTAGATTCACTTTCACCACTGTCATC
AB298886.1  GCATGGAGTTATTGGAAGGAAGGAAGAGCGCTAGAAATCGTAGATCCAGATATCGTAGATTCATTGTCACCTTTGTCATC
M76647.1    GTATGGAGTCGTTGGAAGGAAGGAAGAGCGCTAGAAATCGTAGATCCCGTCATCGTAGATTCACTGTCATCACAGCCATC
AB298905.1  GCATGGAGTCATTGGGCGGAGGGAAGAGCGCTAGAAATCGTAGATCCAGTCATCGTAGATTTGTTGTCATCTCTGCCATC
AB298884.1  GTATGGAGTCAGTGGAAGGAAGGAAGAGCACTAGAAATCGTAGATCCAGTCATCGTAGATTCTTTGTCATCACTGCCATC
AB298901.1  GCATGGAATAATTGGAAGGAAGGAAGAGCGTTAGAAATCGTAGATCCAGATATCGTAGATTCATTTTCACCATTGTCACC
AB298875.1  GCATGGAGTCACTGGAAGGAAGGAAGAGCGCTAGAAATCGTAGATCCAGTAATCGTAGATTCATTGCCATCACTGCCATT
AB298891.1  GCATGGAGTCACTGGAAGGAAGGAAGAGCGCTAGAAATCATAGATCCAGTAATCGTAGATTCATCTCCATCACTGCCATT
U00443.1    GCTTGGAGTCAGTGGGAGGAAGGAAGAGCACTAGAAATCATAGATCCAGTAATCGTAGATTCAT........TGCCATC
AB012106.1  GCATGGAGTCACTGGAAGGAAGGAAGAACACTAGAACTCGTAGATCCAGTCATCGTAGATTCATC......ACTGCCATC
AB298902.1  GCATGGAGTCACTGGAAGGAAGGAAGAGCGCTAGAAATCGTAGATCCTGTCATCGTAGATTCATCGTCATCACTGCCATC
AB013720.1  GCATGGAGTAACTGGAAGGAAGGAAGGGCACTAGAAATTGTAGATTCAGTACTCGTAGATTCATTGTCACCACTGTCATC
AB032473.1  GCATGGAGTAATTGGAAGGAAGGAAGAGCGCTAGAAATCGTAGATCCAGTCATTGTAGATTCATTGTCACCACTGTCATC
AB052756.1  GCGTGGAGAAACTGGAAGGAAGGAAAAGGCCTAGAAATCCTAGATCCATTCATCGTAGATTCATCATCATCT...CCTTC

                 2320      2330      2340      2350      2360      2370      2380
AB270775.1  AACATTTCAACCAAAAGAAGTTCTAAAATGCATACAAATTGGTCTCTTGTGTGTTCAAGAACGTGCGGAGCAT.......
AB270776.1  AACATTTCAACCAAAAGAAGTTCTAAAATGCATACAAATTGGTCTCTTGTGTGTTCAAGAACGTGCGGAGCATCAGAGCC
M97667.1    AACCTTTCAACCAAAAGAAGTTCTAAAATGCATACAAATTGGTCTCTTGTGTGTTCAAGAACGTGCAGAGCAT.......
AB032474.1  AACATTTCAACCAAAAGAAGTCCTAAAATGCATACAAATTGGTCTATTGTGTATTCAAGAACGTGCGGAGCAC.......
AB298890.1  AACATTTCAACCAAAAGAAGTCCTAAAATGCATACAAATTGGTCTATTGTGTATTCAAGAACGTGCGGAGCAC.......
AB219163.1  AACATTTAAACCAAAAGAAGTCCTAAAATGCATACAAATTGGTCTATTGTGTATTCAAGAACGTGCGGAGCAC.......
OR887605    AACATTTAAACCAAAAGAAGTCCTAAAATGCATACAAATTGGTCTATTGTGTATTCAAGAACGTGCGGAGCAC.......
AB054061.1  AACATTTAAACCAAAAGAAGTCCTAAAATGCATACAAATTGGTCTATTGTGTATTCAAGAACGTGCGGAGCAC.......
AB298885.1  AACATTTAAACCAAAAGAAGTCCTAAAATGCATACAAATTGGTCTATTGTGTATTCAAGAACGTGCGGAGCAC.......
D38564.2    AACATTTAAACCAAAAGAAGTCCTAAAATGCATACAAATTGGTCTATTGTGTATTCAAGAACGTGCGGAGCAC.......
AB270777.1  AACATTTAAACCAAAAGAAGTCCTAAAATGCATACAAATTGGTCTATTATGTATTCAAGAACGTGCGGAGCAC.......
D38563.1    AACATTTAAACCAAAAGAAGTCCTAAAATGCATACAAATTGGTCTATTGTGTATTCAAGAACGTGCGGAGCAC.......
EU180597.1  AACATTTCAACCAAAAGAAGTCCTAAAATGCATACAAATTGGTCTATTGTGTATTCAAGAACGTGCGGAGCAC.......
AB298887.1  AACATTTAAACCAAAAGAAGTCCTAAAATGCATACAAATTGGTCTATTGTGTATTCAAGAACGTGCGGAGCAC.......
L08607.1    ACCATTTCAACTACAAGAAGTCCTAAAATGCATACAAATTGGTCTCTTGTGTGTTCAAGAACTTGCAGAGAAC.......
AB086976.1  ACCATTTCAACTACAAGAAGTCCTAAAATGCATACAAATTGGTCTCTTGTGTGTTCAAGAACTTGCAGAGAAC.......
AB270767.1  ACCATTTCAACTACAAGAAGTCCTAAAATGCATACAAATTGGTCTCTTGTGTGTTCAAGAACTTGCAGAGAAC.......
LC556298.1  AACATTTCAACCACAAGAAGTCCTAAAATGCATACAAATTGGTCTCTTGTGTGTTCAAGAACTTGCAGAACAC.......
D30049.1    AACATTTCAACCACAAGAAGTCCTAAAATGCATACAAATTGGTCTCTTGTGTGTTCAAGAACTTGCAGAACAC.......
AB298886.1  AACATTACAACCACAAGAAGTCCTAAAATGCATACAAATTGGTCTCTTGTGTGTTCAAGACCTTGCAGAGCAC.......
M76647.1    AATATTTCAACCACAAGAAGTCCTAAAATGTATTCAAATTGGTCTCTTGTGTGTTCAAGAACTTGCAGAGCAC.......
AB298905.1  AACATTTCAACGAAAAGAAGTCCTAAAATGCATACAAATTGGTCTATTGTGTATTCAAGAACGTGCGGAGCAC.......
AB298884.1  AACATTTCAACCACAAGAAGTCCTAAAATGCATCCAAATTGGTCTCTTGTGTGTTCAAGAACATGCAGAGCAT.......
AB298901.1  AACAATTCAACCACAAGAAGTCCTAAAATGCATAAAAATTGGTCTCTTGTGTGTTCAAGAACTTGCAGAGCAC.......
AB298875.1  AACATCTCAACCACAAGAAGTCCTAAAATGCATCCAAATTGGTCTCTTGTGTGTTCAAGAACGTGCGGAGCAC.......
AB298891.1  AACATCTCAACCACAAGAAGTCCTAAAATGCATCCAAATTGGTCTCTTGTGTGTTCAAGAACGTGCGGAGCAC.......
U00443.1    AACATTTCAACCACAAGAAGTCCTAAAATGCATCCAAATTGGTCTCCTGTGTGTTCAAGAACTTGCAGAGCAC.......
AB012106.1  AACATTTCAACCAGAAGAAGTCCTAAAGTGCATACAAATTGGTCTCTTGTGTGTTCAAGAACTTGCAGAGCAC.......
AB298902.1  AACATTTCAACCACAAGAAGTCCTAAAATGCATACAAATTGGTCTCTTGTGTGTTCAAGAATTTGCAGAGCAC.......
AB013720.1  AACATTTCAACCACAAGAAGTCCTAAAATGCATACAGATTGGTCTCTTGTGTGTCCAGGAACTTGCAGAGCAC.......
AB032473.1  AACATTTCAACCACAAGAAGTCCTAAAATGCATACAAATTGGTCTCTTGTGTGTTCAAGAACTTGCAGAGCAC.......
AB052756.1  AGCGTTTCGACCACATGAAGTCCTAAGATGCATACAAATTGGTCTCTTGTGTGTTCAAGAACGTGCCGAGGAT.......

AB270775.1  ................................................................................
AB270776.1  GGACCTGCAAAAATTGGGGCCCCATTCAAATTTTTTTTTTTACAATCAAATAATTACATTTAAACAAAACAAATCATACA
M97667.1    ................................................................................
AB032474.1  ................................................................................
AB298890.1  ................................................................................
AB219163.1  ................................................................................
OR887605    ................................................................................
AB054061.1  ................................................................................
AB298885.1  ................................................................................
D38564.2    ................................................................................
AB270777.1  ................................................................................
D38563.1    ................................................................................
EU180597.1  ................................................................................
AB298887.1  ................................................................................
L08607.1    ................................................................................
AB086976.1  ................................................................................
AB270767.1  ................................................................................
LC556298.1  ................................................................................
D30049.1    ................................................................................
AB298886.1  ................................................................................
M76647.1    ................................................................................
AB298905.1  ................................................................................
AB298884.1  ................................................................................
AB298901.1  ................................................................................
AB298875.1  ................................................................................
AB298891.1  ................................................................................
U00443.1    ................................................................................
AB012106.1  ................................................................................
AB298902.1  ................................................................................
AB013720.1  ................................................................................
AB032473.1  ................................................................................
AB052756.1  ................................................................................
```

```
AB270775.1  ................................................................................
AB270776.1  TATCTTATATAATTTCACACTATAATATAACTCATTTAATCTTTTTAACAATTATATGTAAATATATAAATTCAAAAGTT
M97667.1    ................................................................................
AB032474.1  ................................................................................
AB298890.1  ................................................................................
AB219163.1  ................................................................................
OR887605    ................................................................................
AB054061.1  ................................................................................
AB298885.1  ................................................................................
D38564.2    ................................................................................
AB270777.1  ................................................................................
D38563.1    ................................................................................
EU180597.1  ................................................................................
AB298887.1  ................................................................................
L08607.1    ................................................................................
AB086976.1  ................................................................................
AB270767.1  ................................................................................
LC556298.1  ................................................................................
D30049.1    ................................................................................
AB298886.1  ................................................................................
M76647.1    ................................................................................
AB298905.1  ................................................................................
AB298884.1  ................................................................................
AB298901.1  ................................................................................
AB298875.1  ................................................................................
AB298891.1  ................................................................................
U00443.1    ................................................................................
AB012106.1  ................................................................................
AB298902.1  ................................................................................
AB013720.1  ................................................................................
AB032473.1  ................................................................................
AB052756.1  ................................................................................

AB270775.1  ................................................................................
AB270776.1  ATGTTTTGATGTGTATTATATCAAAAACTTAAAAAAAAAACTAATATATAGAATTTTTTTAAAAATAGGGGCCCTTAAA
M97667.1    ................................................................................
AB032474.1  ................................................................................
AB298890.1  ................................................................................
AB219163.1  ................................................................................
OR887605    ................................................................................
AB054061.1  ................................................................................
AB298885.1  ................................................................................
D38564.2    ................................................................................
AB270777.1  ................................................................................
D38563.1    ................................................................................
EU180597.1  ................................................................................
AB298887.1  ................................................................................
L08607.1    ................................................................................
AB086976.1  ................................................................................
AB270767.1  ................................................................................
LC556298.1  ................................................................................
D30049.1    ................................................................................
AB298886.1  ................................................................................
M76647.1    ................................................................................
AB298905.1  ................................................................................
AB298884.1  ................................................................................
AB298901.1  ................................................................................
AB298875.1  ................................................................................
AB298891.1  ................................................................................
U00443.1    ................................................................................
AB012106.1  ................................................................................
AB298902.1  ................................................................................
AB013720.1  ................................................................................
AB032473.1  ................................................................................
AB052756.1  ................................................................................

                                                                       2390      2400
AB270775.1  .............................................................AGACCAACGATGTCGTCAG
AB270776.1  ATGTTGGGGCCCCATTCAAATGTTTCATAGAAATGGGCTCAGGCCCGGCTCTGCGGAGCATAGACCAACGATGTCGTCAG
M97667.1    .............................................................AGACCAACGATGTCGTCCG
AB032474.1  .............................................................AGACCAACGATGTCGTCGG
AB298890.1  .............................................................AGACCAACGATGTCGTCGG
AB219163.1  .............................................................AGACCAACGATGTCGTCGG
OR887605    .............................................................AGACCAACGATGTCGTCGG
AB054061.1  .............................................................AGACCAACGATGTCGTCGG
AB298885.1  .............................................................AGACCAACGATGTCGTCGG
D38564.2    .............................................................AGACCAACGATGTCTTCGG
AB270777.1  .............................................................AGACCAACGATGTCGTCGG
D38563.1    .............................................................AGACCAACGATGTCGTCGG
EU180597.1  .............................................................AGACCAACGATGTCGTCGG
AB298887.1  .............................................................AGACCAACGATGTCGTCGG
L08607.1    .............................................................AGACCAACCATGTCGTCTG
AB086976.1  .............................................................AGACCAACCATGTCGTCTG
AB270767.1  .............................................................AGACCAACCATGTCGTCTG
LC556298.1  .............................................................AGACCAACAATGTCGTCTG
D30049.1    .............................................................AGACCAACAATGTCGTCTG
AB298886.1  .............................................................AGACCAACGATGTCGTCTG
M76647.1    .............................................................AGACCAGCGATGTCGTCTG
AB298905.1  .............................................................AGACCAACGATGTCGTCGG
AB298884.1  .............................................................AGACCAATGATGTCGTCTG
AB298901.1  .............................................................AGACCAACAATGTCGTCAG
AB298875.1  .............................................................AGACCAACGATGGCGTCGG
AB298891.1  .............................................................AGACCAACGATGTCGTCGG
U00443.1    .............................................................AGACCAACGATGTCGTCTG
AB012106.1  .............................................................AGGCCAACAATGTCGTCTG
AB298902.1  .............................................................AGGCCAACAATCTCGTCTG
AB013720.1  .............................................................AGACCAACGATGTCGTCTG
AB032473.1  .............................................................AGACCAACGATGTCGACTG
AB052756.1  .............................................................AGACCAGTGATGTCGTCTG
```

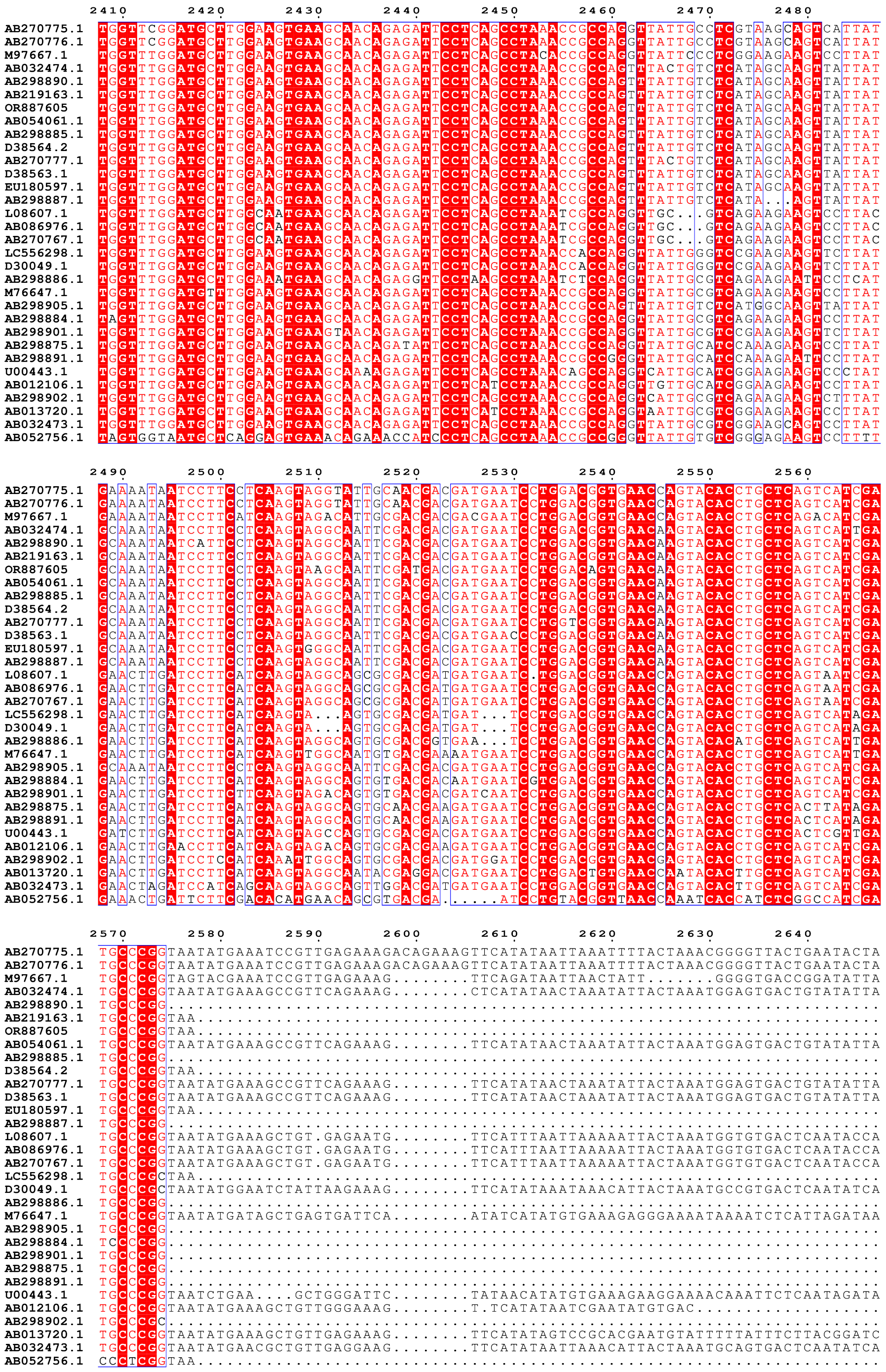

```
           2650      2660      2670      2680      2690      2700      2710      2720
AB270775.1  TGTGTGAAAGAAT.TAATATTTCAATAGATAAATTTCTTGTTATTTTGGGAAAAAGAATTCTTATTTTCATAACCAATTC
AB270776.1  TGTGTGAAAGAAT.TAATATTTCAATAGATAAATTTCTTGTTATTTTGGGAAAAAGAATTCTTATTTTCATAACCAATTC
M97667.1    TAAGTGAAAGAAAATAAAATTTCAATAGTTAAGT...TTGTTATTTGATAACCAAATCTTGTTATTTCCTGGTGGTGTTG
AB032474.1  TAAGTGATCGAAGGACAATAAATTCTCAGTAGATAAGTTTGTTATATTGATAACCAATTCATGTTATTTTCTGGTGAAGT
AB298890.1  ................................................................................
AB219163.1  ................................................................................
OR887605    ................................................................................
AB054061.1  TAAGTGATCGAAGGAAAATAAATTCTCAGCAGATAAGTTTGTTATATTGATAACCAATT.............GGTGAAGT
AB298885.1  ................................................................................
D38564.2    ................................................................................
AB270777.1  TAAGTGAA........................................................................
D38563.1    TAAGTGATCGAAGGAAAATAAATTCTCAGTAGATAAGTTTGTTATATTGATAACCAATTCTTGTTATTTTCTGGTGAAGT
EU180597.1  ................................................................................
AB298887.1  ................................................................................
L08607.1    TATGTGAAAGAAGTAAAATAAATTCTCAATAGAAA.............................................
AB086976.1  CATGTGAAAGAA....................................................................
AB270767.1  TATGTGAAAGAAGTAAAATAAATTCTCAATAGATAAAGTATGTTATTTTGATAACAAATTCTTGCGTCTTTTTTCTGGCG
LC556298.1  ................................................................................
D30049.1    CATGTGAAAGAAGGAAAATAAATTCTTAATAGTTAA.GTATGTTATTTTGATAACAAATTCTTGTTTTTTCTAGCTGTAT
AB298886.1  ................................................................................
M76647.1    GTAGGTTATTTCGATAACCACTTCTTGTTATTTTCTGGCGGTGTTGTCATTATCCCCTTTATATTAAAAAGAAGCATTTG
AB298905.1  ................................................................................
AB298884.1  ................................................................................
AB298901.1  ................................................................................
AB298875.1  ................................................................................
AB298891.1  ................................................................................
U00443.1    AGTATGTTATTTTGATGACCTATTTTTGTTATTTTCTTGCGGTGTTGTCATTATTCAAAATCTATAATAACACATTATTG
AB012106.1  ................................................................................
AB298902.1  ................................................................................
AB013720.1  AAAATTTTAATGCATGATAAAATTGTTATATACTATTTAAAAATTGATGATATATATTTTATACTGA.............
AB032473.1  TATGTGAAAGAAGGAAATAAATTCTCAAAATATAAGTATGTTATTTTGTAAC............................
AB052756.1  ................................................................................

              2730      2740      2750      2760      2770      2780      2790
AB270775.1  TTGTTATTTTCTGGTGGTGTTCATATTCAAAGTACCATATTAAATGATTCCGGTTCCCCTATTA................
AB270776.1  TTGTTATTTTCTGGTGGTGTTCATATTCAAAGTACCA...........................................
M97667.1    TCATATTCGTTTTTCTGAATGAATGTTAAAGTTATTATTC........................................
AB032474.1  TGTCATTATTCATAGTACAATAATACATGCTGGAGCGCCTTGTCGGCACAAATGCGTGTTAGCTTTTTGTTTTGTGTGAT
AB298890.1  ................................................................................
AB219163.1  ................................................................................
OR887605    ................................................................................
AB054061.1  TGTCATTATTCATAGTACAATAATACATGCTGGAGCGTCTTGTCGGCACAAAAAAA........................
AB298885.1  ................................................................................
D38564.2    ................................................................................
AB270777.1  ................................................................................
D38563.1    TGTCATTATTCAAAGTACAATAATAAATGCTGGAGCGTCTTGTCGGC.................................
EU180597.1  ................................................................................
AB298887.1  ................................................................................
L08607.1    ................................................................................
AB086976.1  ................................................................................
AB270767.1  GTTCAAAGTTTTTTTTTTTCCTCGATGATATTAAACCAAATTGAAAGGACTACAAGGTCGGATACGTTTTTAGGACACCA
LC556298.1  ................................................................................
D30049.1    CTATCTTATTAAAATATAATTACACTTAT...................................................
AB298886.1  ................................................................................
M76647.1    TATTAAATCCCCTTGCCTCAAGAGATATTCACAAGAATACTATTGTGACGTGACAGCCTCACTATCGTTTAAACATTACA
AB298905.1  ................................................................................
AB298884.1  ................................................................................
AB298901.1  ................................................................................
AB298875.1  ................................................................................
AB298891.1  ................................................................................
U00443.1    C...............................................................................
AB012106.1  ................................................................................
AB298902.1  ................................................................................
AB013720.1  ................................................................................
AB032473.1  ................................................................................
AB052756.1  ................................................................................

AB270775.1  .....................................................
AB270776.1  .....................................................
M97667.1    .....................................................
AB032474.1  ACAGATAATTGTGAGATATTCAAACTGCAACCGCTCATTTTTTAAGTTGTTTG
AB298890.1  .....................................................
AB219163.1  .....................................................
OR887605    .....................................................
AB054061.1  .....................................................
AB298885.1  .....................................................
D38564.2    .....................................................
AB270777.1  .....................................................
D38563.1    .....................................................
EU180597.1  .....................................................
AB298887.1  .....................................................
L08607.1    .....................................................
AB086976.1  .....................................................
AB270767.1  AAACCAGCTTTCAAACAGATGAAACTGGAGACATAAGCTCCTA..........
LC556298.1  .....................................................
D30049.1    .....................................................
AB298886.1  .....................................................
M76647.1    ATGCTGACGTGTGGCTTGTAAATAGCTTCTCAGACC.................
AB298905.1  .....................................................
AB298884.1  .....................................................
AB298901.1  .....................................................
AB298875.1  .....................................................
AB298891.1  .....................................................
U00443.1    .....................................................
AB012106.1  .....................................................
AB298902.1  .....................................................
AB013720.1  .....................................................
AB032473.1  .....................................................
AB052756.1  .....................................................
```

Multiple sequence alignment of gene sequences of *FER1* selected for phylogenetic study

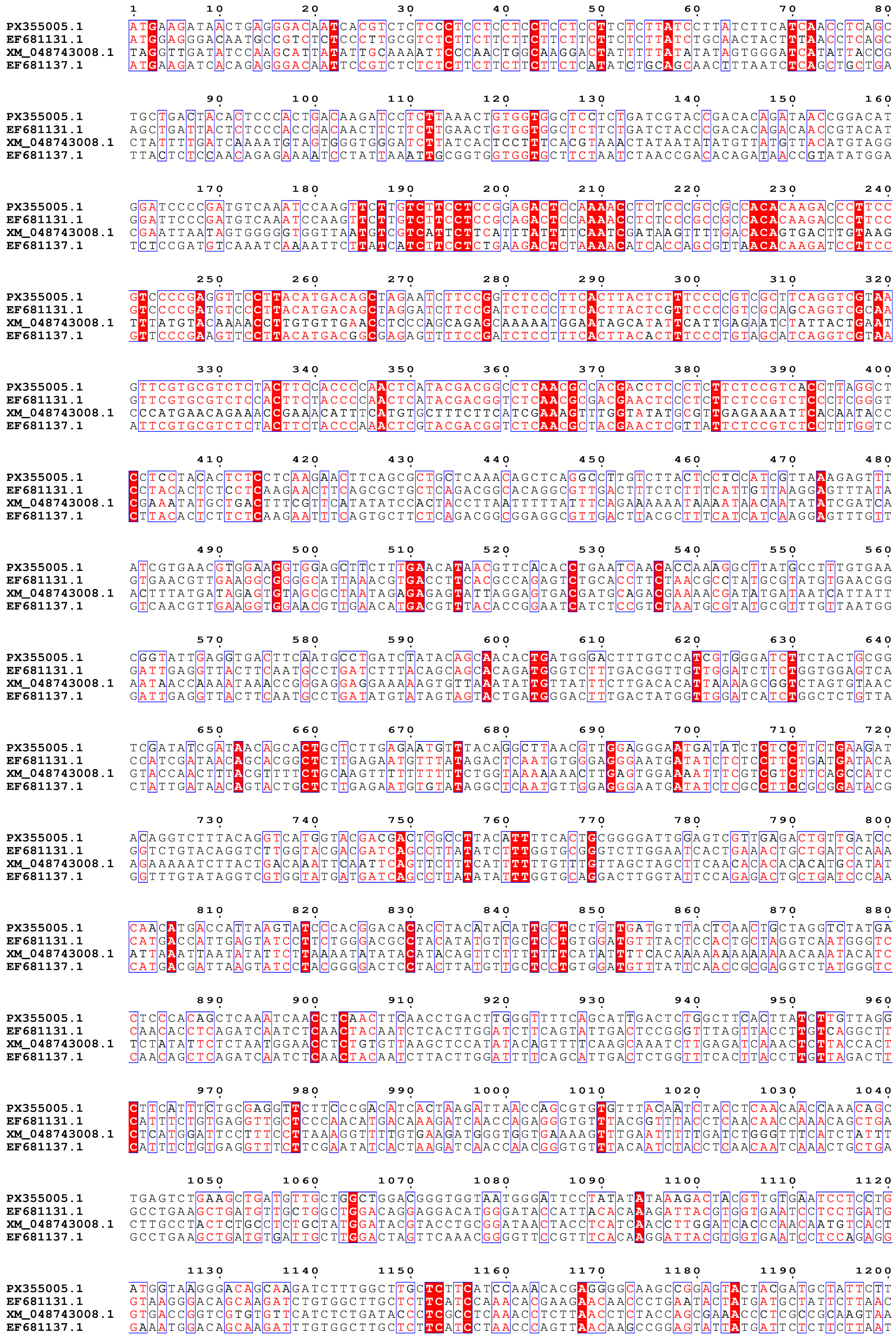

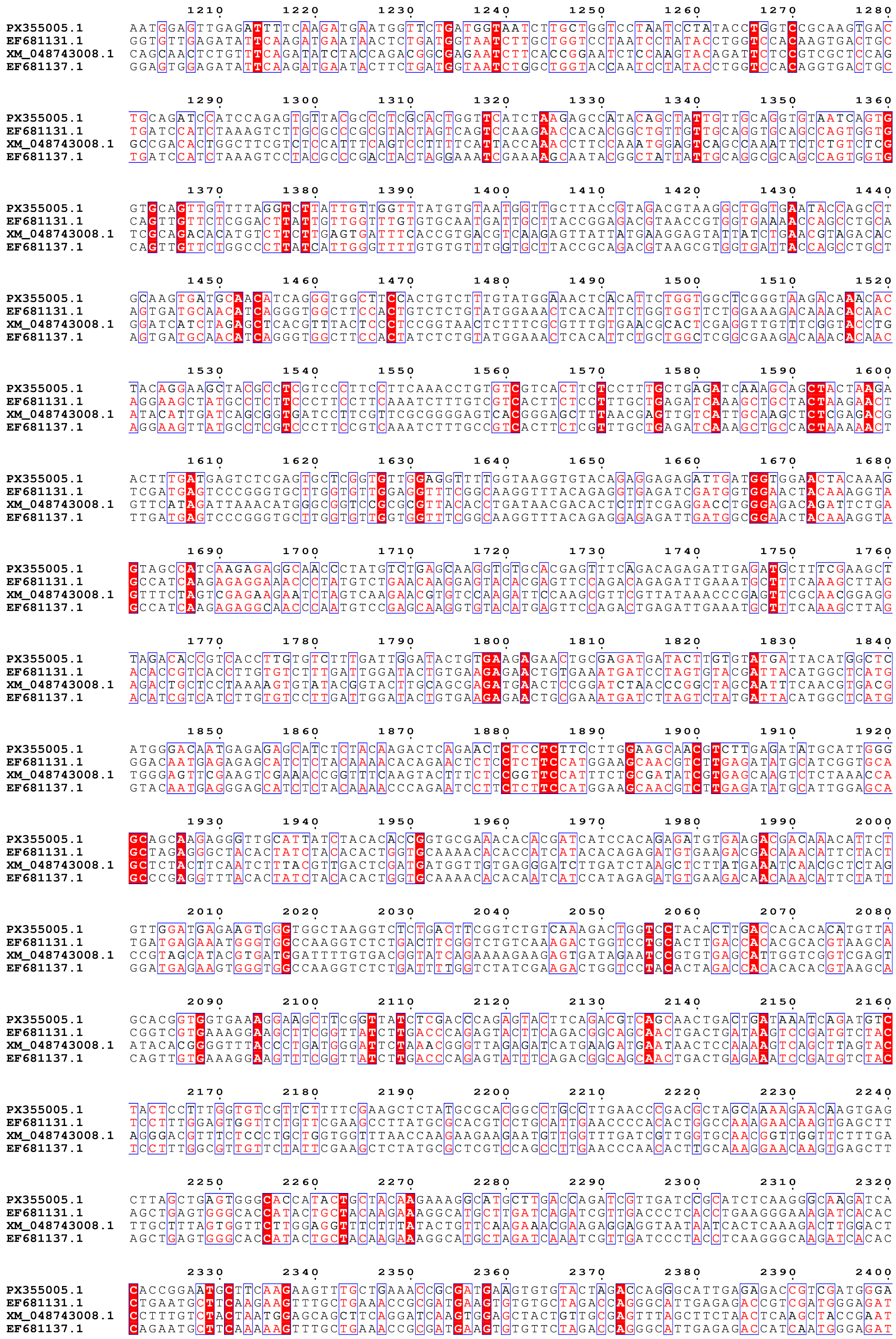
PX355005.1
EF681131.1
XM_048743008.1
EF681137.1

```
                          2410      2420      2430      2440      2450      2460      2470      2480
PX355005.1       GATGTTCTCTGGAACTTAGAGTTCGCGTTGCAGCTTCAGGAAAGCGCTGAGGAGAGCGGGAAAGGGATATGCAGTGAGAT
EF681131.1       GTCCTGTGGAACCTGGAGTTCGCGTTGCAGCTTCAGGAAAGCGCAGAGGAGAGCGGGAAAGGAATATGCAGTGAGATGGA
XM_048743008.1   CCCTCTTGCAGCGGTTAAAGAGGCGACGAACAGCTTCGACGAGAACCGTGCTATTGGAGTGGGAGGTTTTGGTAGAGTGT
EF681137.1       GTTCTGTGGAACTTAGAATTTGCGTTGCAGCTCCAGGAAAGCGCAGAAGAGAACGGAAAAGGAGTATGCGGTGACATGGA

                          2490      2500      2510      2520      2530      2540      2550      2560
PX355005.1       GGACATGGGTGAGATTAAGTACGATGATGATAACTGTAAAGGGAAGAGCAACAACGACAAGGGCTCTGATGTGTATGAAG
EF681131.1       CATGGATGAGATCAAGTACGATGATGACAACTGTAAAGGGAAGAACAACAGCGAGAAAGGTTGTGATGTGTATGAAGGGA
XM_048743008.1   ACAAAGGAGTGCTTCAAGACGGCACGCAAGTAGCTGTTAAAAGAGGCAACCCAAAGTCTCAGCAAGGCTTTGAGGAGTTC
EF681137.1       CATGGATGAGATTAAGTACGATGATGGAAACTGTAAAGGAAAGAACGACAAGAGTTCTGATGTGTATGAAGGGAATGTGA

                          2570      2580      2590      2600      2610      2620      2630      2640
PX355005.1       GGAATGTTAGTGACTCGAGGAGCAGTGGTATAGACATGAGTATTGGTGGTAGGAGTTTGGTCAGTGAAGATTCAGATGGA
EF681131.1       ACGTGACAGACTCGAGGAGCAGTGGAATAGACATGAGCATAGGTGGTCGGAGTTTGGCAAGTGAAGATTCAGATGGACTC
XM_048743008.1   AAGACGGAGATCGAGATGTTGTCTCAGTTCCGACACCGACATTTGGTGTCTTTGATCGGTTACTGCGACGAGAACAGGGA
EF681137.1       CGGACTCGAGGAGCAGTGGAATAGATATGAGCATCGGTGGTCGGAGTTTGGCCAGCGAAGATTCAGATGGACTCACTCCA

                          2650      2660      2670      2680      2690
PX355005.1       CTCACTCCAAGTGCTGTGTTTTCTCAGATCATGAATCCTAAGGGACGTTAG.............................
EF681131.1       ACTCCTAGTGGTGTGTTTTCTCAGATAATGAATCCCAAGGGACGTTAG................................
XM_048743008.1   GATGATTCTTGTTTACGAGTACATGGAGAACGGAACGTTAAAGAGTCATCTCTACGGCTCTGGTTTACCTACCTTGAGTT
EF681137.1       AGTGCTGTGTTTTCTCAGATCATGAATCCAAAGGGACGTTAG......................................

PX355005.1       ................................................................................
EF681131.1       ................................................................................
XM_048743008.1   GGAAGCAACGTCTCGAGATATGTATTGGATCAGCTAGAGGGTTGCATTATCTCCACACCGGTGACTCTAAATCAGTGATC
EF681137.1       ................................................................................

PX355005.1       ................................................................................
EF681131.1       ................................................................................
XM_048743008.1   CACAGAGATGTGAAGTCTGCAAACATTTTGCTAGACGAGAATCTCATGGCGAAAGTTGCGGACTTTGGACTGTCGAAGAC
EF681137.1       ................................................................................

PX355005.1       ................................................................................
EF681131.1       ................................................................................
XM_048743008.1   CGGACCAGAGATAGACCAGACTCATGTGAGTACTGCTGTGAAAGGAAGCTTCGGTTATCTCGACCCTGAGTACTTTAGAA
EF681137.1       ................................................................................

PX355005.1       ................................................................................
EF681131.1       ................................................................................
XM_048743008.1   GACAGCAGCTCACTGAGAAGTCAGATGTTTACTCGTTCGGAGTCGTTATGTTCGAGGTTCTATGCGCGAGGCCCGTTATA
EF681137.1       ................................................................................

PX355005.1       ................................................................................
EF681131.1       ................................................................................
XM_048743008.1   GACCCGACGCTTCCTAGAGAGATGGTGAATCTTGCGGAATGGGCGATGAAATGGCAGAAGAAAGGACAGCTTGAACATGT
EF681137.1       ................................................................................

PX355005.1       ................................................................................
EF681131.1       ................................................................................
XM_048743008.1   TATTGATCAGTCCTTGCGCGGTGAGATCGTACCTGATTCGCTGAGGAAGTTTGGTGAGACGGGGGAGAAGTGTTTAGCTG
EF681137.1       ................................................................................

PX355005.1       ................................................................................
EF681131.1       ................................................................................
XM_048743008.1   ATTATGGAGTTGATAGGCCGTCGATGGGAGATGTGTTGTGGAATCTTGAGTATGCTTTGCAGCTTCAAGAAGCTGGGGTT
EF681137.1       ................................................................................

PX355005.1       ................................................................................
EF681131.1       ................................................................................
XM_048743008.1   GATTGTGATCAAGAAGATGATAATAGTACCAACATGATCGGTGAGTTGCCTTTACGGTTTAATGATTATAACAACCGTGG
EF681137.1       ................................................................................

PX355005.1       ................................................................................
EF681131.1       ................................................................................
XM_048743008.1   AGACACGAGTGTTAGTGTTGGTGTAGTAACTAGAGAGGGACGGTTTGGAGAAGGAGAAGAAGAAGAGTCGTGTGTTGATG
EF681137.1       ................................................................................

PX355005.1       ................................................................................
EF681131.1       ................................................................................
XM_048743008.1   ATCTTTCAGGTGTTTCCATGAGTAAAGTCTTCTCACAGCTCGTTAAATCTGAAGGACGATAAGAATCTTTGTTCTGCTCA
EF681137.1       ................................................................................

PX355005.1       ................................................................................
EF681131.1       ................................................................................
XM_048743008.1   AACTTTTATTTCTTCTGTACATGATTAACGAGTAGACTGTGATTTAATTAAGTAACCGGTTCGCTTCGGTTTAATCACCT
EF681137.1       ................................................................................
```

Multiple sequence alignment of gene sequences of *MLPK* selected for phylogenetic study

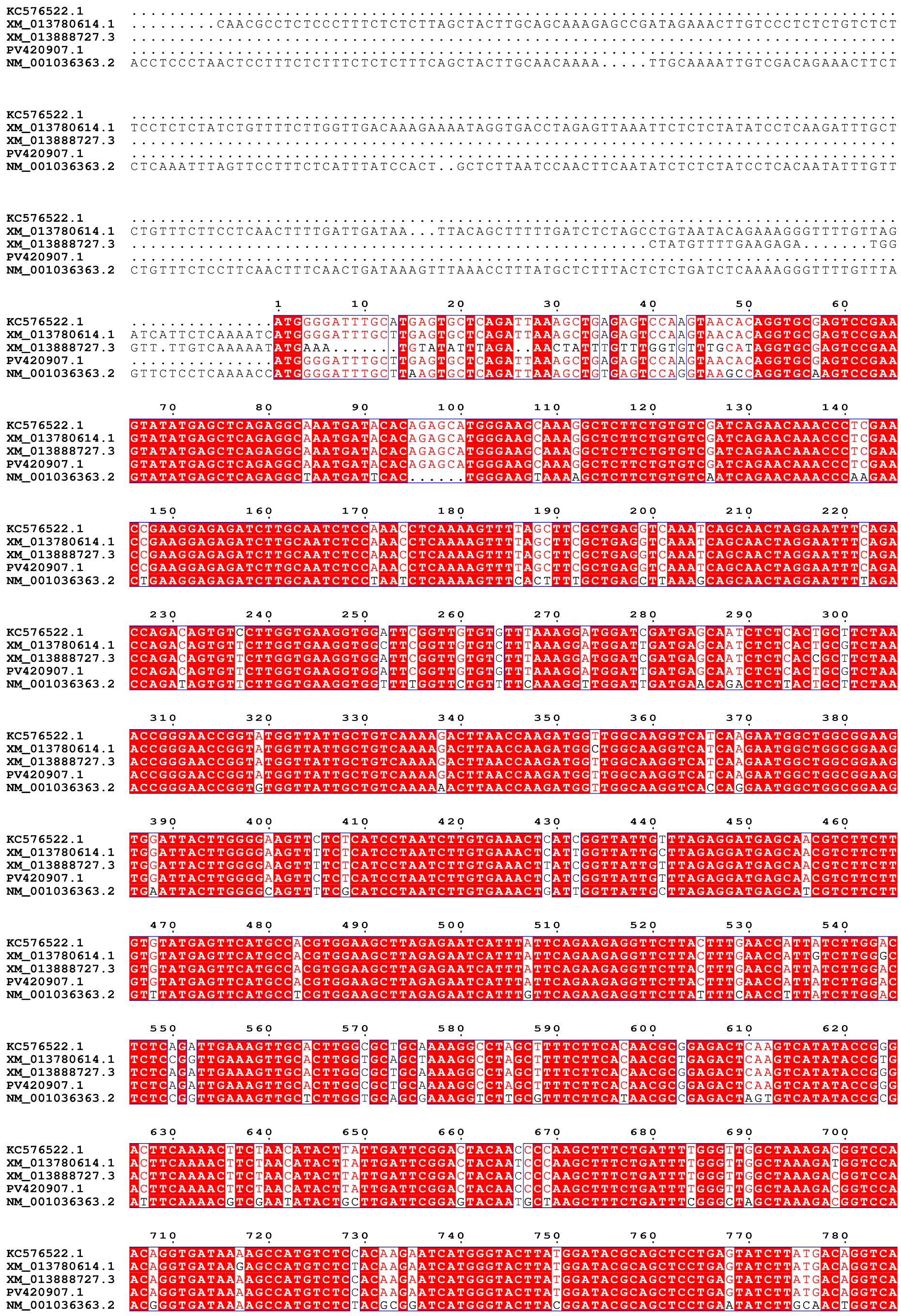

```
               790       800       810       820       830       840       850       860
KC576522.1     TTTAACAACCAAGAGTGATGTCTATAGCTACGGTGTTGTGCTTTTGGAGATACTCTCTGGACGTAGAGTTGTAGACAAGA
XM_013780614.1 TTTAACAACGAAGAGTGATGTCTACAGCTACGGTGTTGTGCTTTTGGAGATACTCTCTGGACGTAGAGTTGTAGATAAGA
XM_013888727.3 TTTAACAACCAAGAGTGATGTCTATAGCTACGGTGTTGTGCTTTTGGAGATACTCTCTGGACGTAGAGTTGTAGACAAGA
PV420907.1     TTTAACAACCAAGAGTGATGTCTATAGCTACGGTGTTGTGCTTTTGGAGATACTCTCTGGACGTAGAGTTGTAGACAAGA
NM_001036363.2 TTTAACAACCAAAAGTGATGTCTATAGCTACGGTGTTGTGCTTTTGGAGGTGTTGTCTGGACGGAGAGCTGTAGACAAGA

               870       880       890       900       910       920       930       940
KC576522.1     ACCGTCCACCGGGAGAGCAAAAACTGGTGGATTGGGCAAAACCGTTGCTTGCAAACAAGAGGAAGATCTTTAGAGTTATC
XM_013780614.1 ACCGTCCACCGGGAGAGCAAAAGCTGGTGGACTGGGCTAAGCCGTTGCTTGCAAACAAGAGGAAGATATTCCGAGTTATC
XM_013888727.3 ACCGTCCACCGGGAGAGCAAAAACTGGTGGATTGGGCAAAACCGTTGCTTGCAAACAAGAGGAAGATCTTTAGAGTTATC
PV420907.1     ACCGTCCACCGGGAGAGCAAAAACTGGTGGATTGGGCAAAACCGTTGCTTGCAAACAAGAGGAAGATCTTTAGAGTTATC
NM_001036363.2 ACCGTCCACCAGGAGAGCAAAAGCTAGTGGAATGGGCAAGACCGTTACTTGCTAACAAAAGGAAGTTATTCCGAGTTATC

               950       960       970       980       990       1000      1010      1020
KC576522.1     GATAACCGTCTACAAGATCAGTACTCAATGGAAGAAGCGTGTAAAGTAGCTACTCTAGCGCTGAGATGCCTGACGACAGA
XM_013780614.1 GATAACCGTCTCCAAGGTCAGTACTCCATGGAAGAAGCGTGTAAAGTAGCTACTCTAGCGCTGAGATGCCTGACGATAGA
XM_013888727.3 GATAACCGTCTACAAGATCAGTACTCAATGGAAGAAGCGTGTAAAGTAGCTACTCTAGCGCTGAGATGCCTGACGACAGA
PV420907.1     GATAACCGTCTACAAGATCAGTACTCAATGGAAGAAGCGTGTAAAGTAGCTACTCTAGCGCTGAGATGCCTGACGACAGA
NM_001036363.2 GATAACCGTCTACAAGATCAATACTCAATGGAAGAAGCTTGTAAAGTAGCTACTCTTGCGCTGAGATGCCTCACATTCGA

               1030      1040      1050      1060      1070      1080      1090      1100
KC576522.1     GATAAAGCTGAGACCAAACATGACTGAGGTTGTTGCTCACCTCGAACACATACAAACTTTGCATGAAACAGGAGGAGGAA
XM_013780614.1 GATAAAGCTGAGACCAAACATGACTGAGGTTGTTGCTCACCTCGAACACATACAAACTTTGCATGAAACAGGAGGAGGAA
XM_013888727.3 GATAAAGCTGAGACCAAACATGACTGAGGTTGTTGCTCACCTCGAACACATACAAACTTTGCATGAAACAGGAGGAGGAA
PV420907.1     GATAAAGCTGAGACCAAACATGACTGAGGTTGTTGCTCACCTCGAACACATACAAACTTTGCATGAAACAGGAGGAGGAA
NM_001036363.2 GATAAAGCTGAGACCAAACATGAACGAGGTTGTTTCTCACCTCGAACACATCCAAACTTTGAATGAAGCAGGAGGA...A

               1110      1120      1130      1140      1150      1160      1170      1180
KC576522.1     GAAACATTGATAAGTTGGAGAGGAGAACGCGTAGGAGAAGTGATAGTGTTGTGGTGAGCCAAAAACCTAATGCTGGTTTC
XM_013780614.1 GAAACATTGATAAGATGGAGAGGAGAACGCGTAGGAGAAGTGATAGTGTTGTGGTGAGCCAAAAATCAAATGCTGGTTTT
XM_013888727.3 GAAACATTGATAAGTTGGAGAGGAGAACGCGTAGGAGAAGTGATAGTGTTGTGGTGAGCCAAAAACCAAATGCTGGTTTT
PV420907.1     GAAACATTGATAAGTTGGAGAGGAGAACGCGTAGGAGAAGTGATAGTGTTGTGGTGAGCCAAAAACCAAATGCTGGTTTT
NM_001036363.2 GAAATATTGATATGGTCCAGAGGAGAATGCGTAGGAGAAGCGATAGTGTTGCTATCAACCAAAAACCAAATGCAGGTTTT

               1190      1200      1210      1220      1230      1240      1250
KC576522.1     GCTAGACAAAGTGCTGTGGGTGGAATAGCAGCTGCGTATCCACGTCCGTCTGCTTCGCCTCTGTTTGTCTAA........
XM_013780614.1 GCTAGACAAAGTGCTGTGGGTGGAATAGCAGCTGCGTATCCACGTCCGTCTGCTTCGCCTCTGTTTGTCTGAT.......
XM_013888727.3 GCTAGACAAAGTGCTGTGGGTGGAATAGCAGCTGCGTATCCACGTCCCTCTGCTTCGCCTCTGTTTGTCTGAT.......
PV420907.1     GCTAGACAAAGTGCTGTGGGTGGAATAGCAGCTGCGTATCCACGTCCCTCTGCTTCGCCTCTGTTTGTCTGA........
NM_001036363.2 GCTCGACAAACTGCTGTAGGCGTCATAGCTACTGCTTATCCACGCCCGTCTGATTCGCCTCTGTTTGTTTGAGTACGAAA

KC576522.1     ................................................................................
XM_013780614.1 .....GATGTTCTGTTCTGTTC...TGTTTAGTTCAG.........ATGTACAGTTTTGCTTCT.GTTCCATGGCTCGT.
XM_013888727.3 .....GATGTTCTGTTTAGTTCAGATGTACAGTTTTGTTTCTGCTTATGTATTGAGAGGATTCA.AGTTCATGGCTCGT.
PV420907.1     ................................................................................
NM_001036363.2 CGATGAATGTTCTCTTTAGTTCGGATGTACAGTTTTGTTTCTGCTTGTGTACCGAAAGGATTCTCAGTTCATGGCTCGTT

KC576522.1     ................................................................................
XM_013780614.1 ..CAGAGATTTGACAATTGATGTTTGTAGTGCTGAGAGTTGACTAACATAAAGTAAAAATGTTG.TCTCATGTCTTCAAC
XM_013888727.3 ..CAGAGATTTGACAATTGATGTTTGTAGTGCGGAGAGTTGACTAACAAAAAGTAAAAATGATGGTCTCATGTCTTCAA.
PV420907.1     ................................................................................
NM_001036363.2 TACACGACTCAGAGATTTGAC...........................................................

KC576522.1     .....................................
XM_013780614.1 TTGAAGCTTTGTTATTTAATAAGAAGATTTACAGTAA
XM_013888727.3 .....................................
PV420907.1     .....................................
NM_001036363.2 .....................................
```

Multiple sequence alignment of gene sequences of *ARC1* selected for phylogenetic study

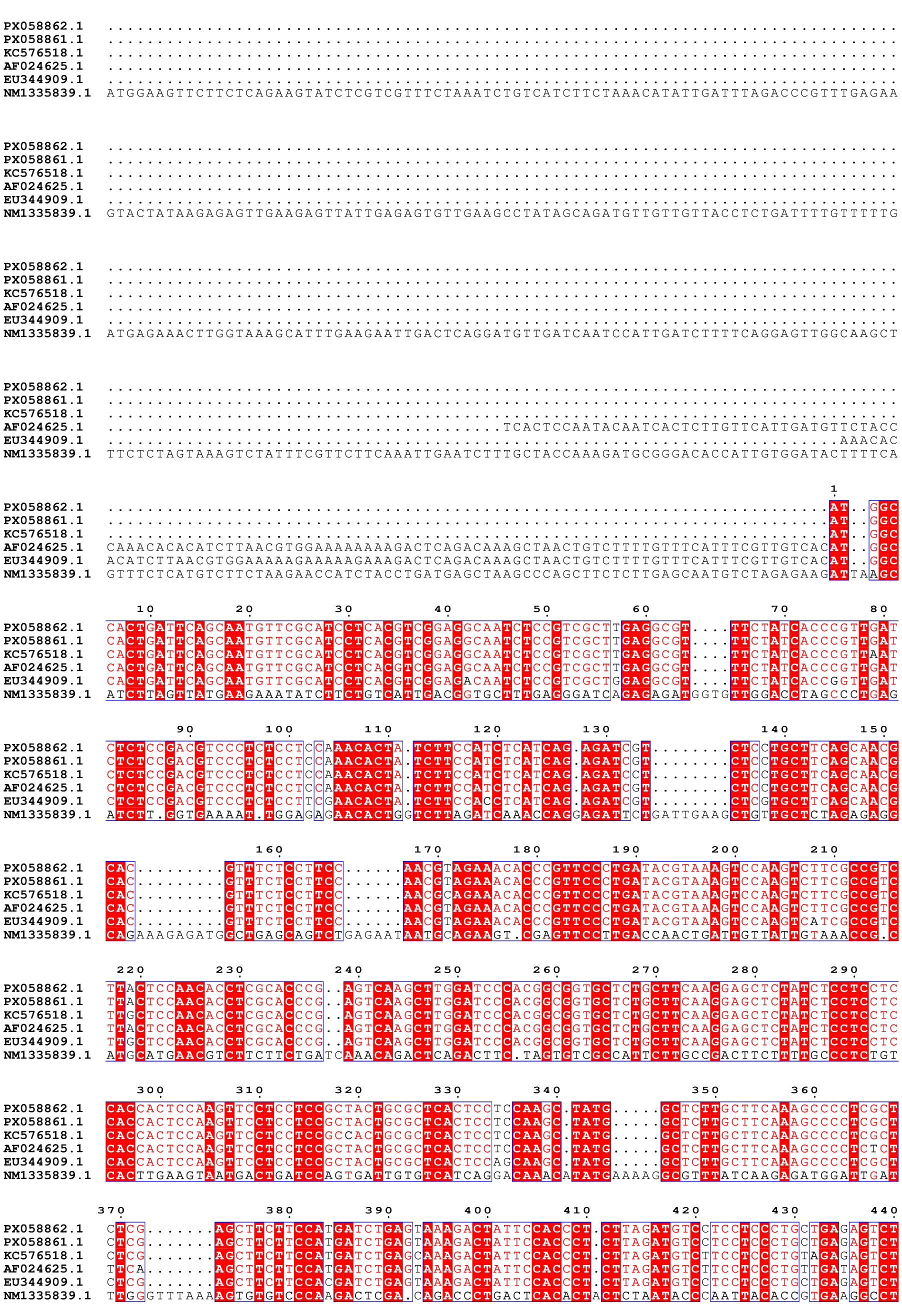

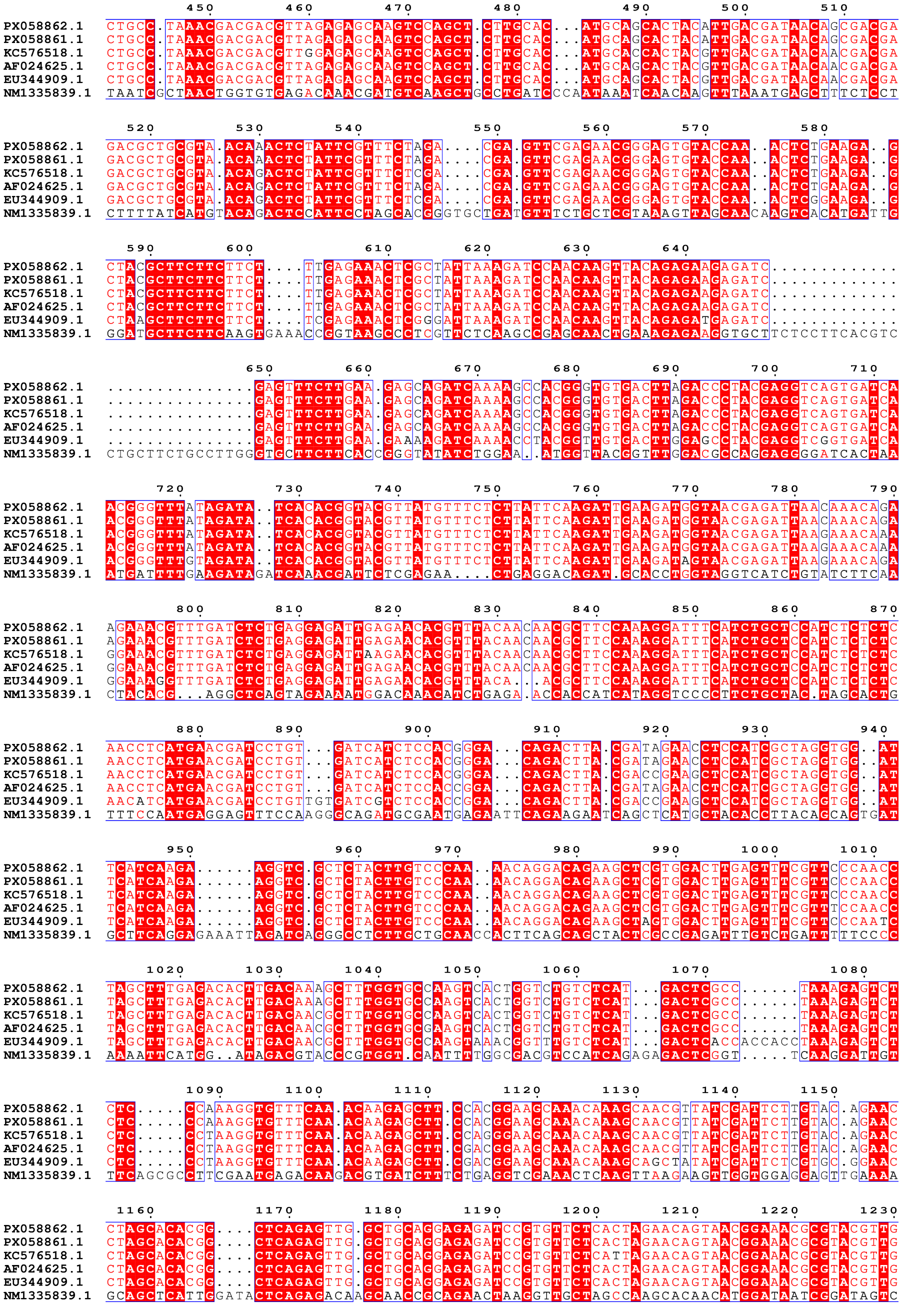
PX058862.1
PX058861.1
KC576518.1
AF024625.1
EU344909.1
NM1335839.1

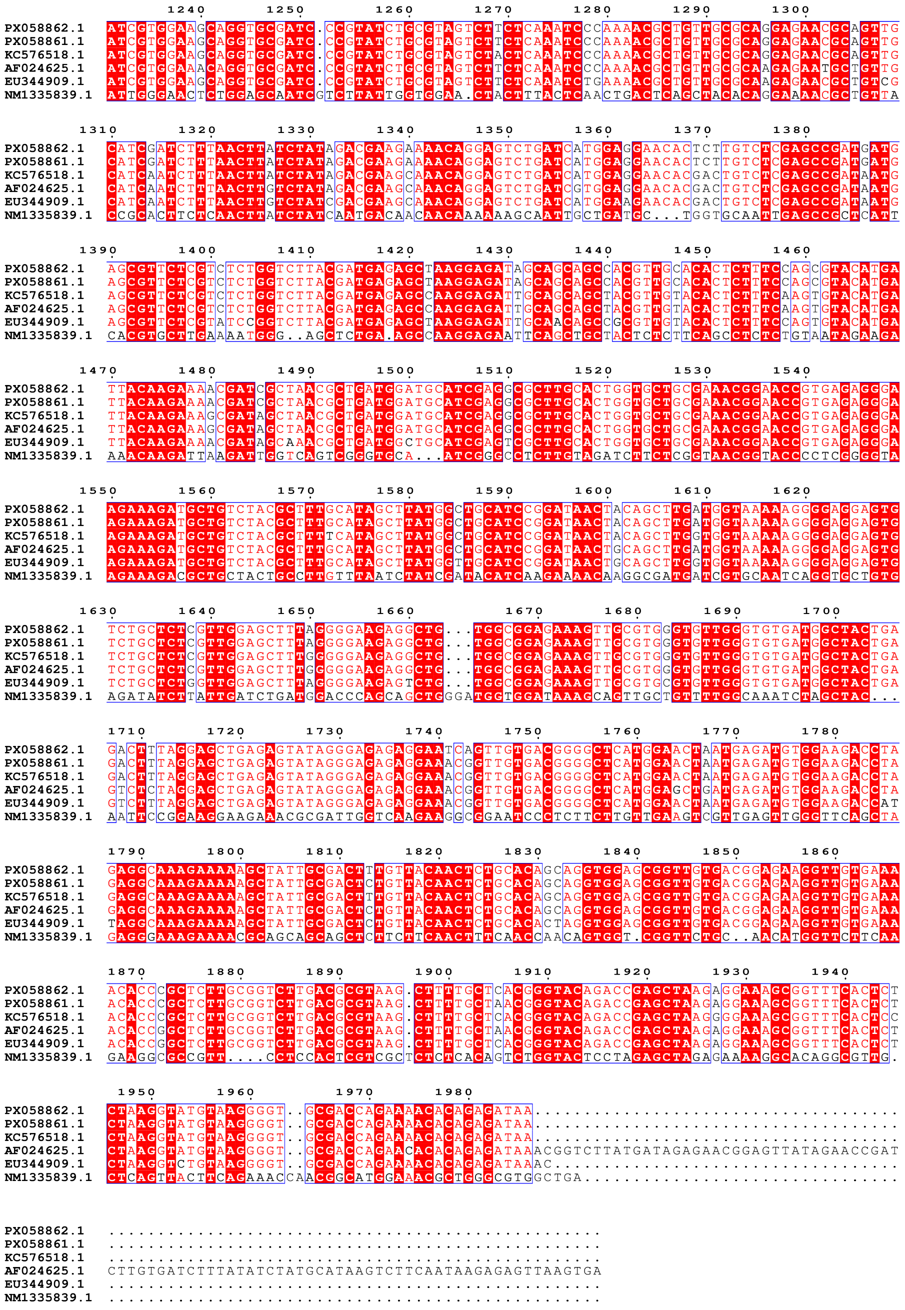

Supplement: Supplementary file 3 [file DataSheet3.pdf]
